# Environmental gradients reveal stress hubs pre-dating plant terrestrialization

**DOI:** 10.1038/s41477-023-01491-0

**Published:** 2023-08-28

**Authors:** Armin Dadras, Janine M. R. Fürst-Jansen, Tatyana Darienko, Denis Krone, Patricia Scholz, Siqi Sun, Cornelia Herrfurth, Tim P. Rieseberg, Iker Irisarri, Rasmus Steinkamp, Maike Hansen, Henrik Buschmann, Oliver Valerius, Gerhard H. Braus, Ute Hoecker, Ivo Feussner, Marek Mutwil, Till Ischebeck, Sophie de Vries, Maike Lorenz, Jan de Vries

**Affiliations:** 1 Institute of Microbiology and Genetics, Department of Applied Bioinformatics, University of Goettingen, Goettingen, Germany; 2 Campus Institute Data Science, University of Goettingen, Goettingen, Germany; 3 Albrecht-von-Haller-Institute for Plant Sciences, Department of Plant Biochemistry, University of Goettingen, Goettingen, Germany; 4 Institute of Plant Biology and Biotechnology, Green Biotechnology, University of Münster, Münster, Germany; 5 Goettingen Center for Molecular Biosciences, Service Unit for Metabolomics and Lipidomics, University of Goettingen, Goettingen, Germany; 6 Section Phylogenomics, Centre for Molecular Biodiversity Research, Leibniz Institute for the Analysis of Biodiversity Change, Museum of Nature, Hamburg, Germany; 7 Institute for Plant Sciences and Cluster of Excellence on Plant Sciences, Biocenter, University of Cologne, Cologne, Germany; 8 Faculty of Applied Computer Sciences and Biosciences, Section Biotechnology and Chemistry, Molecular Biotechnology, University of Applied Sciences Mittweida, Mittweida, Germany; 9 Institute of Microbiology and Genetics and Göttingen Center for Molecular Biosciences and Service Unit LCMS Protein Analytics, Department of Molecular Microbiology and Genetics, University of Goettingen, Goettingen, Germany; 10 Goettingen Center for Molecular Biosciences, Department of Plant Biochemistry, University of Goettingen, Goettingen, Germany; 11 School of Biological Sciences, Nanyang Technological University, Singapore, Singapore; 12 Albrecht-von-Haller-Institute for Plant Sciences, Department of Experimental Phycology and SAG Culture Collection of Algae, University of Goettingen, Goettingen, Germany; 13 Goettingen Center for Molecular Biosciences, Department of Applied Bioinformatics, University of Goettingen, Goettingen, Germany

**Keywords:** Plant evolution, Evolution, Transcriptomics

## Abstract

Plant terrestrialization brought forth the land plants (embryophytes). Embryophytes account for most of the biomass on land and evolved from streptophyte algae in a singular event. Recent advances have unravelled the first full genomes of the closest algal relatives of land plants; among the first such species was *Mesotaenium endlicherianum*. Here we used fine-combed RNA sequencing in tandem with a photophysiological assessment on *Mesotaenium* exposed to a continuous range of temperature and light cues. Our data establish a grid of 42 different conditions, resulting in 128 transcriptomes and ~1.5 Tbp (~9.9 billion reads) of data to study the combinatory effects of stress response using clustering along gradients. *Mesotaenium* shares with land plants major hubs in genetic networks underpinning stress response and acclimation. Our data suggest that lipid droplet formation and plastid and cell wall-derived signals have denominated molecular programmes since more than 600 million years of streptophyte evolution—before plants made their first steps on land.

## Abstract (web summary)

Dadras et al. exposed *Mesotaenium*, an alga of the sister lineage to land plants, to a bifactorial gradient of environmental cues and use comparative analyses to pinpoint conserved circuits in plant genetic response networks.

## Main

Plant terrestrialization changed the face of our planet. It gave rise to land plants (Embryophyta), the major constituents of Earth’s biomass^1^ and founders of the current levels of atmospheric oxygen^2^. Land plants belong to the Streptophyta, a monophyletic group that includes the paraphyletic freshwater and terrestrial streptophyte algae and the monophyletic land plants. Meticulous phylogenomic efforts have established the relationships of land plants to their algal relatives^3–6^. These data brought a surprise: the filamentous and unicellular Zygnematophyceae—and not other morphologically more elaborate algae—are the closest algal relatives of land plants. Now, the first genomes of major orders of Zygnematophyceae (see ref. ^7^) are at hand: *Mesotaenium endlicherianum*^8^, *Spirogloea muscicola*^8^, *Zygnema circumcarinatum*^9^, *Closterium peracerosum–strigosum–littorale*^10^ and *Penium margaritaceum*^11^. Using these, we are beginning to redefine the molecular chassis shared by land plants and their closest algal relatives. Included in this shared chassis will be those genes that facilitated plant terrestrialization. In this Article, we focus on one critical aspect: the molecular toolkit for the response to environmental challenges. For this, we used the unicellular freshwater/subaerial alga *Mesotaenium endlicherianum*.

Land plants use a multi-layered system for the adequate response to environmental cues. This involves sensing, signalling and response, mainly by the production of, for example, protective compounds. Some of the most versatile patterns in land plant genome evolution concern genes for environmental adaptation^12–14^. That said, there is a shared core of key regulatory and response factors that are at the heart of plant physiology. These include phytohormones such as abscisic acid (ABA) found in non-vascular and vascular plants^15,16^, protective compounds resting on specialized metabolic routes such as phenylpropanoid-derived compounds and proteins such as LATE EMBRYOGENESIS ABUNDANT (LEA)^17,18^. Many of the genes integrated into these stress-relevant metabolic routes have homologues in streptophyte algae^19^. Taking angiosperms as reference, such stress-relevant pathways are often patchy. Whether these are also used under the relevant conditions is currently unknown. For example, while Zygnematophyceae have a homologue to the ABA-receptor PYL^8,20^, this homologue works in a different, ABA-independent fashion^21^. Thus, it is important to put the genetic chassis that could act under environmental shifts to the test.

Here we used a fine grid of a bifactorial gradient for two key terrestrial stressors, variation in irradiance and temperature, to probe the genetic network that the closest algal relatives of land plants possess for the responsiveness to abiotic cues. Correlating environmental parameters, physiology and global differential gene expression patterns from 128 transcriptomes (9,892,511,114 reads, 1.5 Tbp of data) across 126 distinct samples covering a temperature range of >20 °C and light range of >500 µmol photons m^−2^ s^−1^, we pinpoint hubs in the circuits that have been shared along more than 600 million years of streptophyte evolution.

## Results

### A physiological grid: co-dependency of eurythermy and euryphoty

**Fig. 1:**
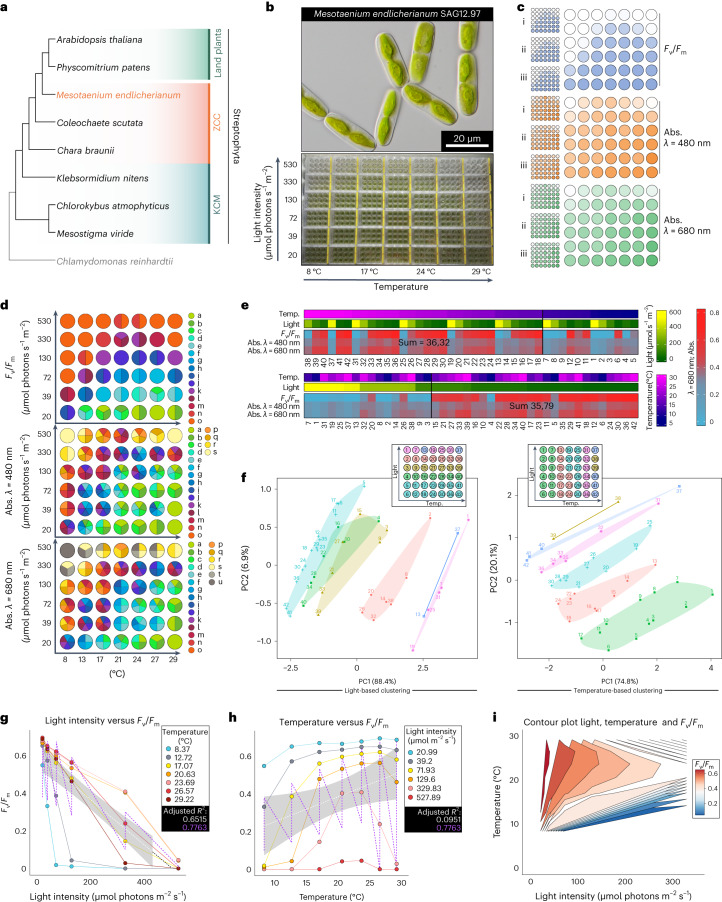
A fine-combed setup for assessing environmental responses in *Mesotaenium*. **a**, Cladogram of Streptophyta, highlighting that *Mesotaenium endlicherianum* SAG 12.97 is a representative of the closest algal relatives of land plants. KCM, the grade of Klebsormidiophyceae, Chlorokybophyceae and Mesostigmatophyceae; ZCC, the grade of Zygnematophyceae, Coleochaetophyceae and Charophyceae. **b**, *M.* *endlicherianum* grown in C-medium in 42 12-well plates on a gradient table that produces a temperature range of 8.6 ± 0.5 °C to 29.2 ± 0.5 °C on the *x* axis and an irradiance gradient of 21.0 ± 2.0 to 527.9 ± 14.0 µmol photons m^−2^ s^−1^ on the *y* axis; for phenotyping per well, at least ten micrographs were taken, all showing similar phenotypes of the cells. **c**, Overview of the measured maximum quantum yield *F*_v_/*F*_m_ as a proxy for gross physiology (blue) and absorption (abs.) at 480 (orange) and 680 nm (green); individual replicates of the biological triplicates are shown on the left and the average values are shown on the right. **d**, Statistical analysis of the physiological values (*F*_v_/*F*_m_, abs. 480 nm, abs. 680 nm). Numbers correspond to environmental conditions on the table. Biological triplicates were grouped into significant groups (a–o, a–s and a–u) with R (version 4.1.3) using a Kruskal–Wallis test coupled with Fisher’s least significance; *P* values were Bonferroni corrected. Significant differences at *P* ≤ 0.001 are shown as letters. **e**, Heat maps displaying averaged physiological values of the 42 conditions sorted either by temperature (temp.) or light. A cut-off was set (black vertical line) on the basis of the distribution of the highest values, which were then summed to determine a positive correlation with temperature or light conditions. **f**, Two PCAs showing the correlation of light conditions (left) or temperature conditions (right) to physiological values (*F*_v_/*F*_m_, abs. 480, 680 nm). Clusters are shown in different colours, which are also visualized in an overview scheme of the gradient table at the top of the plots. **g**,**h**, Unifactorial regression analysis of light intensity (**g**) and temperature (**h**) versus *F*_v_/*F*_m_; note the unifactorial linear regression curves (white) versus the bifactorial (violet). **i**, Contour plot of the bifactorial impact of light and temperature on *F*_v_/*F*_m_ (gradient colour).

We studied the genome-sequenced strain SAG 12.97 of the freshwater alga *Mesotaenium endlicherianum*, a member of the Zygnematophyceae, the closest algal relatives of land plants^8^ (Fig. 1a,b). Natural habitats for *Mesotaenium*, belonging to the order Serritaeniales, are diverse—ranging from plankton to aeroterrestrial^7,8^. We cultivated *Mesotaenium* in a large-scale setup in 1.5 l of C-medium up to a cell density of 0.33 AU at 680 nm and distributed the culture across 504 wells (42 12-well plates, 2.5 ml of culture per well). Well plates were placed on a table with a temperature gradient from 8.6 ± 0.5 °C to 29.2 ± 0.5 °C on the *x* axis; from above, light-emitting diode (LED) lamps created an irradiance gradient from 21.0 ± 2.0 to 527.9 ± 14.0 µmol photons m^−2^ s^−1^ across the *y* axis, thus creating a two-dimensional gradient table (Fig. 1b, Supplementary Table 1 and for light quality, see Extended Data Fig. 4); the conditions were chosen to strike a balance between cell viability and environmental challenge, as determined in a set of pre-experiments (Extended Data Figs. 4–6 and Methods). The 504 cultures were exposed to this gradient setup for 65 h. The physiological status of the algae was assessed by determining the maximum quantum yield of photosystem II (*F*_v_/*F*_m_) using pulse amplitude modulation fluorometry (IMAGING-PAM, Walz) and a microplate reader with absorption at 480, 680 and 750 nm (Fig. 1c, Extended Data Fig. 5 and Supplementary Fig. 1a); the entire procedure was repeated in three successive biological replicates (that is, three runs of the table, 504 *F*_v_/*F*_m_ and 4,536 absorption measurements per replicate).

The algae showed significant differences (*P* ≤ 0.001) in *F*_v_/*F*_m_ values as well as absorption values, both decreasing with rising intensities of irradiance (for *F*_v_/*F*_m_ values at 20.5 ± 1.0 °C: from 0.66 ± 0.02 at a light intensity of 21.14 µmol photons m^−2^ s^−1^ to 0.042 ± 0.04 at a light intensity of 534.7 µmol photons m^−2^ s^−1^) (Fig. 1d, Supplementary Fig. 1 and Supplementary Table 2); despite ample growth at 29.2 ± 0.5 °C and low irradiance, higher temperatures (that is, above 29 °C) were out of the tolerable scope of *Mesotaenium* (Extended Data Fig. 5). We recorded the lowest *F*_v_/*F*_m_ values (down to zero) at conditions of highest irradiance and lowest temperature. Under the ranges tested here, the low temperature had a stronger negative impact on physiology than light. For example, *F*_v_/*F*_m_ values at 8.6 ± 0.5 °C and 133 ± 27 µmol photons m^−2^ s^−1^ are in a different significance group (*P* ≤ 0.001) (group o in Fig. 1d) than *F*_v_/*F*_m_ values at 29.2 ± 0.5 °C at 118 ± 25 µmol photons m^−2^ s^−1^ (purple, group k in Fig. 1d). Values on physiology clustered by light were less broadly distributed than if clustered by temperature (Fig. 1e,f). Even the highest light intensity (527.9 ± 14.0 µmol photons m^−2^ s^−1^) was stressful but tolerable for the physiology of *Mesotaenium* at temperatures between 20.5 ± 0.1 °C (*F*_v_/*F*_m_ = 0.042 ± 0.04) and 25.3 ± 0.1 °C (*F*_v_/*F*_m_ = 0.045 ± 0.04); more extreme temperatures resulted in undetectable *F*_v_/*F*_m_ values. On the basis of the environmental parameters tested herein, eurythermy (broad viable tolerance of temperature) might establish the foundation for euryphoty (broad viable tolerance of light intensities) in *M.* *endlicherianum*. Thus, we used regression analysis to understand the effect and importance of the independent values of light and temperature on the dependent physiological values (Fig. 1g–i, Supplementary Fig. 1b and Supplementary Table 2). We find that physiology was always better explained by a combination of temperature and light than a single parameter alone (for example, for *F*_v_/*F*_m_, *R*^2^ of 0.776 versus 0.652 and 0.095; Fig. 1g–i).

### Fine-combed global gene expression profiles and gene models

**Fig. 2:**
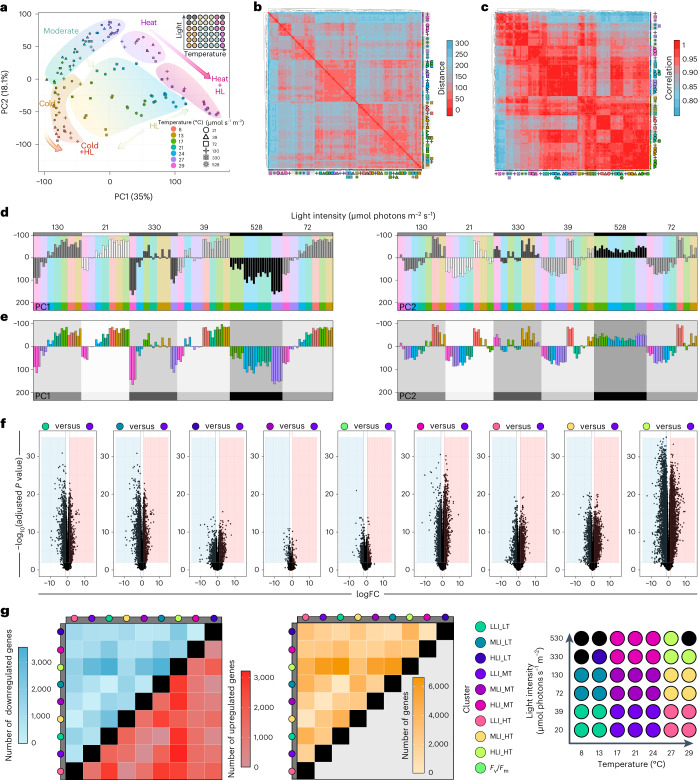
Global profiles of environment-governed gene expression response. **a**, PCA visualizing PC1 and PC2. Backgrounds were drawn to highlight our interpretation of the observed trends; samples are coded by colour (temperature) and symbols (irradiance in µmol photons m^−2^ s^−1^). Samples that did not yield usable RNA are indicated as grey dots in the top-right overview of the experimental setup. **b**, Visualization of Euclidean distances between samples via heat map, from red, zero distance, to blue, furthest distance (a distance of 300). **c**, Heat map of Spearman correlation between samples, from red, maximum correlation (1.0), to blue, least correlation (<0.8). The clusters were calculated via the Euclidean distance. **d**,**e**, PC1 and PC2 scrutinized using a small multiples method of light intensity (**d**) and temperature (**e**). In **d**, shades of grey correspond to different light intensities. In **e**, different colours represent different temperatures and were mapped with the same colours as **a**. To perform differential gene expression analysis, we divided the table into nine sectors (see scheme of the table); additionally, a tenth group was raised based on *F*_v_/*F*_m_ < 0.5. Linear models were fitted for each gene and empirical Bayes statistics computed for DEGs by the limma package. In total, 37 comparisons were made. DEGs were defined as genes with an absolute fold change (FC) ≥2 and Benjamini–Hochberg-adjusted *P* value less than 0.01. **f**, Volcano plots of DEGs for nine selected comparisons based on the sectors and the *F*_v_/*F*_m_ < 0.5 criterion. **g**, Heat maps of numbers of DEGs for all sector-based comparisons (blue, downregulation; red, upregulation; yellow, sum of up- and downregulated genes); grey bars label the first component (treatment) for calculating the contrasts (treatment versus control).

To shed light on the molecular mechanisms that underpin the switch from tolerable conditions to adverse environmental cues in *Mesotaenium*, we applied global gene expression analyses using RNA sequencing (RNA-seq). We pooled all 12 wells per plate and extracted RNA from a total of 126 samples (42 plates, three biological replicates). A total of 114 samples yielded usable RNA that was used to build 128 libraries for sequencing on the Illumina NovaSeq 6000 platform (a minimum of three biological replicates and additional technical replicates; see cartoon of grid in Fig. 2). We generated a total of 1.5 Tbp of 150 bp paired read data at an average depth of 37.7 million reads per sample (~9.9 billion reads in total). Building on this wealth of data, we updated the *Mesotaenium* gene models to V2. V2 has an increased number of protein-coding messenger RNAs, from 11,080 in the original annotation^8^ (V1) to 40,326 protein-coding mRNAs (26,009 high confidence, 14,317 low confidence; including splice variants) in 19,233 genes; we labelled an additional 4,408 mRNAs (in 4,312 genes) as ‘predicted gene’ (Supplementary Table 3). With V2, we bring the number of genes in *Mesotaenium* closer to other Zygnematophyceae with similar genome sizes; V2 has 43 more complete and single copy Benchmarking Universal Single-Copy Orthologs (BUSCO) genes than V1 (+10%; 21 less fragmented, 22 less missing; viridiplantae_odb10; Supplementary Fig. 2). To assess the congruence between biological evidence and V1 and V2, we calculated annotation edit distance metrics (AED; 0 to 1, with 0 being the best). In the cumulative fraction of annotation against AED score, V2 has more mRNAs with AED <0.5. For example, 70% of mRNAs in V1 (7,756 mRNAs) have an AED score <0.5 compared with 60% in V2 (26,840 mRNAs). This is sensible since V2 was built based on the same set of evidence used to calculate AED and it shows higher congruence with them (Supplementary Fig. 3a). Thus, we pseudo-aligned our data onto the new *Mesotaenium* transcriptome V2 (average alignment rate was 87.31%; Supplementary Table 4a).

Cheng et al.^8^ reported that 33.2% of the genome was impacted by transposable elements. We surveyed V2 for protein domains related to transposon biology, retrieving 6,186 entries in 1,748 unique genes (Supplementary Table 4b). Among the 96 that passed the expression threshold, high temperature (29 °C) appeared to have had the strongest effect on transposable element mobilization (Supplementary Fig. 3b).

To understand the gross profile of the gene expression data, we performed a principal component analysis (PCA; Fig. 2a). Independent biological replicates from the same condition clustered in close proximity. High temperature followed by irradiance brought forth clear separation of the data, with PC1 describing 35% and PC2 describing 18.1% of the variance. We evaluated the distance (Fig. 2b) and Spearman correlation (Fig. 2c) using all genes to look for trends among different environmental conditions. The data can be grouped into at least three categories: (1) samples with high light and/or high temperature, (2) a collection of low-temperature (8, 13 and 17 °C) samples, and (3) samples at moderate conditions. Large clusters included low to medium light + medium temperature (‘moderate’ conditions), high light + high temperature, and high light (Fig. 2a). Most distinct was the cluster formed by samples from the high temperature + high light (small multiples; Fig. 2d,e).

### Plastid-related genes stand out in differential gene expression profiles

**Extended Data Fig. 1:**
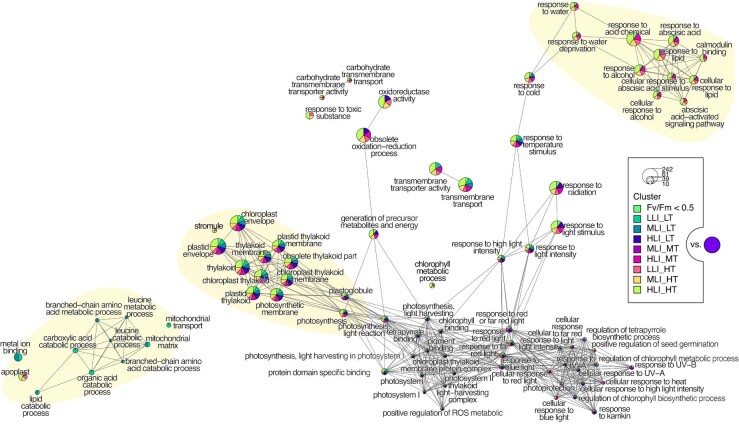
Biological theme comparison summarizing all GO term enrichment analysis with adjusted p value ≤ 0.01 of DEGs against all genes that were expressed and passed the filtering in our analyses as background. The size of each circle is proportional to the count of each GO-term. Only the top 30 enriched terms are shown.

For dissecting the differential gene expression responses, we divided the table into nine sectors and, additionally, a cohort of stressed algae based on *F*_v_/*F*_m_ < 0.5 (Fig. 2f,g). We performed 36 comparisons, among which we focused on nine, which additionally included the *F*_v_/*F*_m_-based comparison. Genes were considered to be differentially expressed between groups at an absolute fold change ≥2 and a Benjamini–Hochberg-corrected *P* ≤ 0.01 (Fig. 2f,g). The intensity of environmental cues governed gross gene expression profiles as increasing disparity between conditions yielded more differentially expressed genes (DEGs), generally following the pattern of the PCA (compare Fig. 2a,g). The most differentially regulated genes (6,578) were pinpointed by comparing low light and low temperature (LLI_LT) versus high light and high temperature (HLI_HT). Enriched Gene Ontology (GO) terms among regulated genes most frequently included plastid biology-associated genes (Extended Data Fig. 1); similar patterns were recovered in 63 unifactorial comparisons where we kept one environmental parameter constant (Extended Data Fig. 7a). To scrutinize our data for specific genes that show a robust and universal response to alterations in the environment, we intersected all 8,157 significantly regulated genes pinpointed by the different comparisons: 3, 30 and 124 genes overlapped among all 9, 8 and 7 comparisons, respectively. These concertedly pinpointed genes were mostly light harvesting genes, corroborating the importance of plastids in the overall cell biology of *Mesotaenium* (Extended Data Fig. 7b). Indeed, the 30 genes found in all comparisons included, for example, reactive oxygen species (ROS)-relevant genes such as *EARLY LIGHT-INDUCIBLE PROTEIN* (*ELIP*) and fatty acid metabolic genes.

**Fig. 3:**
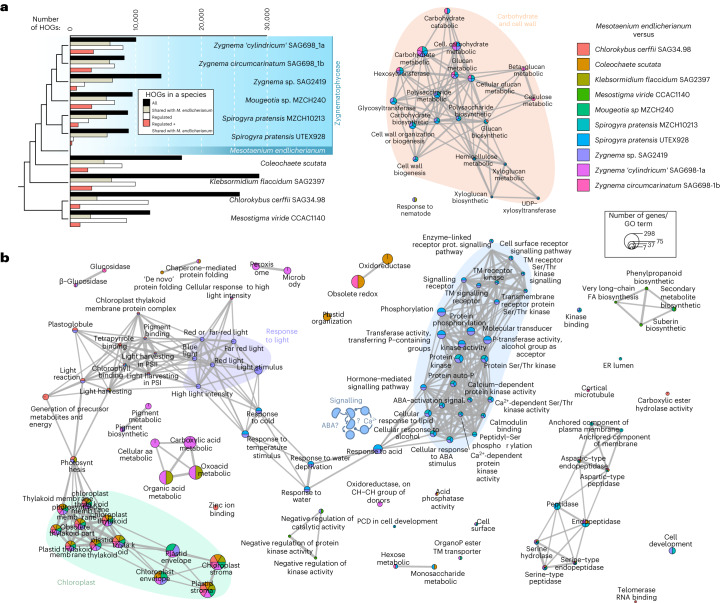
Comparative analyses of global differential gene expression profiles across stress-treated streptophyte algae. Publicly available data on stress transcriptomes from ten different streptophyte algae were downloaded and significant differential gene expression between stress treatment and control per species were calculated. Phylogenetic HOGs were inferred with Orthofinder. **a**, Bar graph of the number of all HOGs detected (black), HOGs shared with *Mesotaenium endlicherianum* (tan), all regulated HOGs in a given species (white) and, of those regulated, which are in the same HOG as significantly regulated genes in *Mesotaenium* (red); the relationship between the streptophyte algae is shown by the cladogram on the left. **b**, GO term-based biological theme comparison of these shared significantly regulated genes in HOGs. Note the recurrent pattern of chloroplast-associated differential gene expression (green), light quality (purple) and the putative integration of calcium signalling with pathways known from phytohormone signalling, including ABA, in land plants (blue, also note the little sketch of a hypothetical model). PCD, programmed cell death.

**Extended Data Fig. 2:**
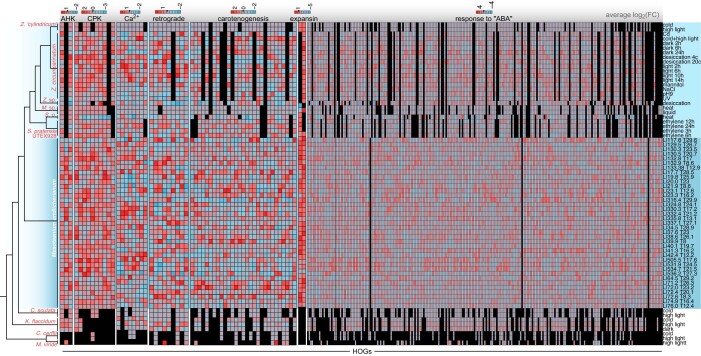
Heat maps of average differential gene expression in log_2_(fold change) per HOG. From the strongest upregulation in red to the strongest downregulation in blue; black means that no HOG was found. The heat maps were sorted by phylogeny (see the cladogram on the left) and treatment (written on the right); light teal highlights the data on Zygnematophyceae, dark teal on *Mesotaenium endlicherianum*.

How do these responses compare across land plants’ close relatives? To answer this, we downloaded major stress transcriptome data from streptophyte algae^9,20,22–25^, inferred significant differential gene expression between stress treatment and control per species, and asked whether regulated genes belong to the same phylogenetic hierarchical orthogroups (HOGs, inferred with Orthofinder^26^). Depending on the phylogenetic distance of the species, we found between 3,107 and 6,458 HOGs shared with *Mesotaenium*, with 46.6–73.0% shared within and 15.8–30.4% outside of the clade Zygnematophyceae (Fig. 3a). Of these shared HOGs, between 4.6% and 59.8% show shared regulation with *Mesotaenium*. The degree of similarity depends on treatment not phylogenetic position. The most common responses across species were related to chloroplasts and photosynthesis (Fig. 3b and Extended Data Fig. 2). However, within Zygnematophyceae, additional signalling processes such as kinase activities and calcium-dependent signalling stood out (Fig. 3b and Extended Data Fig. 2), corroborating (1) their noted importance in Zygnematophyceae^23,24^ and (2) the concept that important steps in the evolution of streptophyte calcium signalling system (Extended Data Fig. 2) occurred before plant terrestrialization^27^.

To understand whether these genes integrate into the context of molecular programmes, we next analysed gene co-expression.

### Gene expression clusters recover ancient genetic programmes

**Fig. 4:**
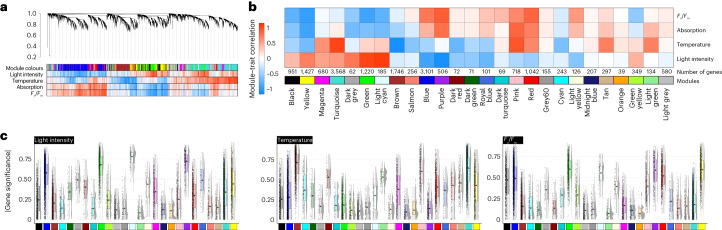
Unsupervised gene expression clusters recover genetic programmes separated by environmental cues. Gene expression clustering into 26 coloured modules was performed using WGCNA; grey is the module of unclustered genes. **a**, Hierarchical cluster tree of 17,095 genes. The heat map below the dendrogram and module colour assignment shows the gene significance measure (from red, positive correlation, to white, no correlation, to blue, negative correlation) for the four different conditions or physiological parameters. **b**, Heat map of the module–trait correlation based on eigengenes (from red, positive correlation, to white, no correlation, to blue, negative correlation); see Supplementary Fig. 7. **c**, Box plots of the mean gene significance across modules (given in the corresponding module colour) towards the parameters light intensity, temperature and *F*_v_/*F*_m_. The box plots display the interquartile range (IQR) of the data, compactly displaying the distribution of a continuous variable. They visualize five summary statistics (the median, two hinges and two whiskers). The upper whiskers extends from the hinges to the largest/smallest value no further than 1.5× IQR from the hinge. Each data point (*n*) is a gene, and the total *n* of genes is the same as shown in **b**. We calculated the gene significance for each gene using the WGCNA package and Pearson method.

**Fig. 5:**
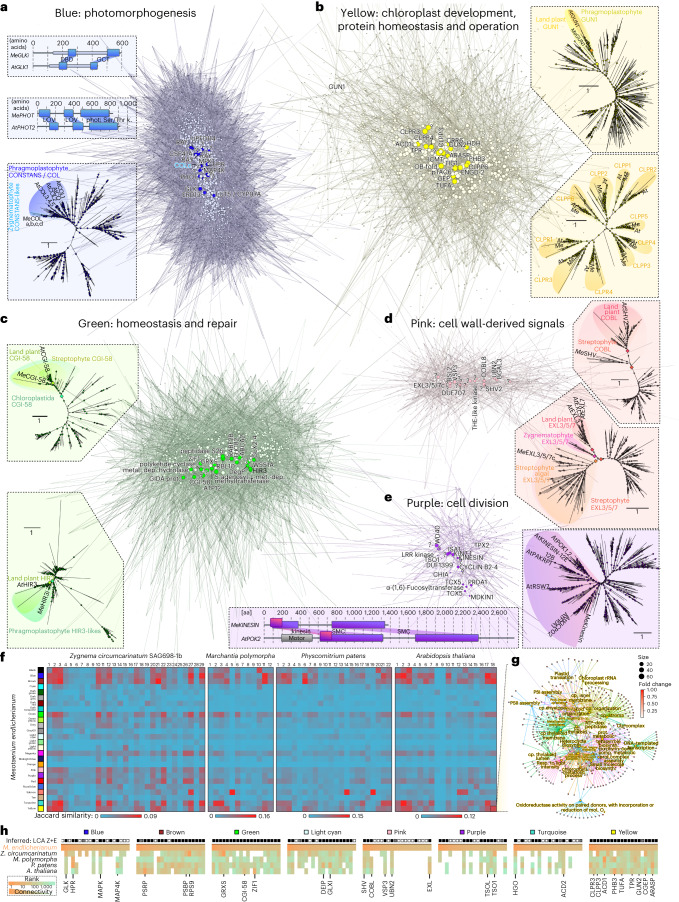
Molecular programmes for environmental responses around recurrent plant hubs. **a**–**e**, Visualization of the co-expression networks clustered by WGCNA into the modules blue (3,101) (**a**), yellow (1,427) (**b**), green (1,220) (**c**), pink (718) (**d**) and purple (506 genes) (**e**). Nodes (circles) represent genes connected by edges whose weight (light to dark colour) is based on a weighted TOM. Brightly coloured nodes represent the 20 most connected genes (hubs) and are annotated based on homology; all other nodes are depicted in the corresponding paler colour. Around the clusters, different protein-coding hub genes are highlighted, giving information such as predicted domain structures or phylogenetic relationships; for fully labelled phylogenies, see Supplementary Fig. 26b. Circles in phylogenies give a scale of the ultrafast bootstrap support values; diamonds indicate high (>90%) support for branches separating highlighted clades. An alignment of GLK homologues can be found in Supplementary Fig. 8. **f**, Using WGCNA, co-expression networks were computed from 212 publicly available RNA-seq datasets from *Z.* *circumcarinatum*, *M.* *polymorpha, P.* *patens* and *A.* *thaliana* exposed to diverse abiotic challenges, yielding between 12 and 29 modules (labelled above the heat map), and orthogroups for all genes in the modules of these different species were determined. The heat map shows the similarity, based on Jaccard indices, between the modules of *Mesotaenium* (same colours as throughout the paper, see Fig. 4b) and the co-expression modules in the three land plants as well as *Zygnema*; red to blue colour gradients indicate high to low Jaccard similarity. **g**, Cnet plot of the enriched GO terms in the module ‘*Arabidopsis* 18’, which has high Jaccard similarity to the *M.* *endlicherianum* module yellow—note the recurrent terms of plastid operation and, especially, the Clp complex. **h**, Heat map of the connectivity ranks across all five species for homologues of hub genes of *Mesotaenium*, from orange (high) to green (low connectivity). Black boxes (top row) indicate if our phylogenies (see data on Zenodo) suggest that the hub genes fall into families that were present in the last common ancestor of Zygnematophyceae and land plants, and hence emerged before plant terrestrialization; white boxes signify the absence of such indication and grey boxes highlight ambiguous relationships.

The environmental gradients triggered changes in the expression of gene cohorts. We wanted to understand their concerted action independent of any prioritization guided by homology to any land plant genes—solely from the molecular programmes that operated in the algae. To do so, we applied a weighted gene co-expression network analysis^28^ (WGCNA) for unsupervised clustering (Fig. 4, Supplementary Figs. 4–7 and 10–13 and Extended Data Fig. 8). To then understand the driving forces behind these changes, we turned to the highly connected genes (nodes) in the network (hubs) (Fig. 5).

**Extended Data Fig. 3:**
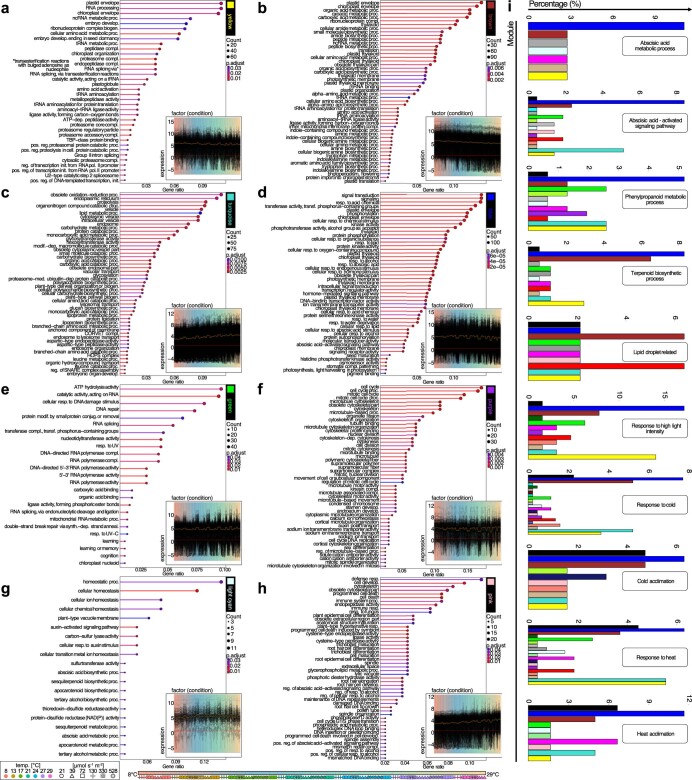
Enriched GO-terms for eight of the 26 modules; each inset shows the gene expression profiles of all genes in a given module. (**a**–**i**) Arabidopsis homologs for key processes were mined based on keywords; they were retrieved from a look-up table of BLASTp hits in a search of *Mesotaenium* V2 against *A. thaliana* representative protein sequences. Bar charts show the percentage of detected *Mesotaenium* homologs across the modules relative to the number of all Arabidopsis IDs assigned to the terms. No BLAST hit was not depicted. Abbreviations: proc. = process; reg. = regulation; biogen. = biogenesis; develop. = development; pos. = positive; neg. = negative; init. = initiation; GEP = Gene expression profile; med. = mediated; dep. = dependent; modif. = modification; conjug. = conjugation; anneal. = annealing; compl. = complex; synth. = synthesis; resp. = response; transf. = transferring.

We clustered the 17,905 genes expressed in our samples (passing the minimum expression threshold) into 26 modules, which we refer to with colours (Fig. 4a). Orange is the smallest module (39 genes), and the largest modules are turquoise, blue and brown with 3,568, 3,101 and 1,746 genes, respectively (Fig. 4b). The samples reflect a range of distinct physiological conditions and resulting data are a combined expression of the different environmental cues and the modulation of the algal physiology. To investigate the biological role of each module, we used their eigengenes as representatives for the modules’ gene expression profiles and correlated their behaviour with the two environmental cues (light intensity and temperature), as well as the physiological parameters absorption and *F*_v_/*F*_m_ (Fig. 4b,c). One of the foremost general patterns in cellular response to stress are ROS. ROS act as signals as well as culprits that, if not quenched, damage biomolecules; GO terms capture ROS biology (Extended Data Fig. 3), especially in module green that positively correlates with light intensity (*r* = 0.88, *P* = 6 × 10^−43^) and negatively with *F*_v_/*F*_m_ (*r* = −0.79, *P* = 6 × 10^−29^) (Extended Data Fig. 3, Supplementary Figs. 4–7 and Supplementary Tables 5 and 6).

The clusters also recover the genetic signatures of thriving algae. Module purple negatively correlates with increasing light (*r* = −0.94, *P* = 3 × 10^−60^) and positively with absorption and *F*_v_/*F*_m_ (*r* = 0.71, *P* = 3 × 10^−21^ and *r* = 0.79, *P* = 4 × 10^−28^). These dense and physiologically healthy cell populations (experiencing no light stress) likely ramped up cell division (Extended Data Fig. 3 and Supplementary Table 6), signified by homologues of cyclin and *TPX2* appearing as hub genes (Fig. 5e). The ninth most connected hub is a kinesin homologous to genes coding for proteins such as PHRAGMOPLAST ORIENTING KINESIN 2, and homologues of the important growth regulators^29^ Tesmin and TSO1 ranked at positions 7, 15 and 17 of the most connected hubs in module purple (Fig. 5e and Supplementary Table 7).

To understand the evolutionary conservation of the genetic programmes in these modules, we processed 212 publicly available RNA-seq datasets from *Zygnema circumcarinatum*^9^, *M.* *polymorpha*, *P.* *patens* and *A.* *thaliana* exposed to diverse abiotic challenges using the same WGCNA pipeline, which yielded between 12 and 29 modules. We determined orthogroups between the modules of these different species and compared the similarity in modules by calculating Jaccard indices (Fig. 5f) and GO-term enrichment in these modules (Fig. 5g and Supplementary Fig. 14b–e). Also here, blue, brown, turquoise and yellow stand out as important and likely conserved environmental response modules (compare Figs. 4b and 5f and Extended Data Fig. 3). We further analysed shared connectivity of hub orthogroups. For the mentioned Tesmin and *TSO1* orthogroups (Fig. 5h), this reveals that they are likely connected regulators of cell division since about 600 million years of streptophyte evolution. To scrutinize this aspect, we inferred the evolutionary history of the 160 hubs using maximum likelihood phylogenetic analyses (Fig. 5h; data on Zenodo). We retrieved 135 phylogenies, 107 of which indicate that the hubs are in gene families that were present in (or before) the last common ancestor of Zygnematophyceae and land plants. Thus, they pre-date plant terrestrialization.

### Conserved hubs: integration of plastid and cell physiology

Chloroplasts act as environmental sensors in land plant cells^30^. In concert with this, many of the modules we identified were associated with plastid biology and/or physiology (Extended Data Fig. 3, Supplementary Figs. 4–7 and Supplementary Table 6). Module brown is enriched in GO terms related to plastids, general transcription and translation, and negatively correlates with temperature (*r* = −0.95, *P* = 7 × 10^−65^; Extended Data Fig. 3 and Supplementary Fig. 5). Among the top 20 hub genes in module brown, 12 are associated with translation and ribosomes (Supplementary Table 7). As expected, this cluster shows conservation in enriched functions of its related modules in the other four streptophytes, including shared high connectivity of hubs (Fig. 5f,h). The module light cyan positively correlates with increasing light (*r* = 0.93, *P* = 1 × 10^−56^; Supplementary Fig. 6) and negatively with *F*_v_/*F*_m_ (*r* = −0.67, *P* = 5 × 10^−18^; Supplementary Fig. 4). It features not only hubs related to ROS homeostasis from the thioredoxin superfamily and other light-induced proteins, but also pigment and apocarotenoid metabolism (Extended Data Fig. 3); these are the source of important signals from the chloroplast that likely have deep evolutionary roots^19^ and are also formed by light-dependent oxidative reactions^31^. The blue module negatively correlates with increasing light (*r* = −0.76, *P* = 10^−25^) and positively with *F*_v_/*F*_m_ (*r* = 0.67, *P* = 2 × 10^−18^). Concomitantly, the blue module has a high number of enriched GO terms, many of which are plastid-related terms, cellular signalling and terms that tie the two together—that is, signalling processes emanating from the plastid (Extended Data Fig. 3 and Supplementary Table 6). Such responses align with similar clusters in other species (Fig. 5f), where the related *Arabidopsis* modules 2 and 10 show terms for light intensity and quality (Supplementary Fig. 14b).

The hubs of many modules, including those in blue, light cyan and yellow mentioned before, reflect an association with plastid-related processes. To highlight a few, the second most connected gene in module blue is a homologue of *GOLDEN2-LIKE 1* (*GLK1*) (Supplementary Fig. 8). GLK1 is a transcription factor (TF) that regulates chloroplast development and the activity of nuclear genes involved in photosynthetic light reaction and chlorophyll biosynthesis^32–34^; indeed, genes in the *GLK* orthogroup are highly connected throughout the modules of land plants, and in the zygnematophyte, *Zygnema* a *GLK* homologue is the eighth most connected gene in its module (Fig. 5h). Blue also features hydroxypyruvate reductase-coding gene, important in photorespiration^35^, as the fourth most connected hub, which appeares in the top-five most connected genes in the bryophytes (Fig. 5h). A CYP450 gene homologous to *LUTEIN DEFICIENT 5* (*LUT5*), is the seventh most connected gene, suggesting the involvement of pigment-related signalling. Module 21 in *P.* *patens* is dominated by ABA signalling (Supplementary Fig. 14d) and it is similar to *Mesotaenium* modules turquoise and blue (Fig. 5f), enriched in homologues of ABA-activated signalling (Extended Data Fig. 3), featuring a highly connected homologue of *ABA-RESPONSIVE ELEMENT-BINDING FACTOR 2* (*ABF2*). Thus, parts of the ABA signalling module consist of ancient wires whose relevance in environmental response pre-date plant terrestrialization, and ABA dependency^20,21,36^.

Next to *GLK1*—the most connected TF-coding gene—other highly connected transcriptional regulators appear in module blue. These include homologues of photomorphogenesis-regulating genes such as *CONSTANS-like 3* (*COL3*, the fourth most connected TF-coding gene) and *CONSTITUTIVE PHOTOMORPHOGENIC 1* (*COP1*); CO/COL and GLKs are both degradation targets of COP1 (refs. ^37–39^). Further, the circadian regulator^40^ *BROTHER OF LUX ARRHYTHMO* is the second most connected TF-coding gene in module blue. All of this aligns with the similarity to the *Arabidopsis* module 2 and the *P.* *patens* module 6, featuring, next to light quality, also photoperiodism (Fig. 5f and Supplementary Fig. 14e,f). Further, homologues of *ETHYLENE-INSENSITIVE3-like 1* (the sixth most connected TF-coding gene) and several *ETHYLENE RESPONSE FACTOR*s (*ERF*s) are among the most connected TF-coding genes. Previous investigations of the Zygnematophyceae *Spirogyra pratensis* have shown that *SpEIN3* can rescue *Arabidopsis* *ein3-1* mutant plants^41^; exogenous application of ethylene on *Spirogyra* triggers stress-, plastid- and photosynthesis-associated gene expression responses similar to land plants^22^, which we recover, as outlined, across co-expression modules (Fig. 5f, Extended Data Fig. 3 and Supplementary Fig. 14a–f) and shared differential patterns (Fig. 3b and Extended Data Fig. 2). This speaks for a conserved regulatory framework that involves the plastid, photosynthesis, ethylene-associated factors, and maybe ethylene itself, in environmental signalling cascades in the common ancestor of land plants and their closest algal relatives.

Module yellow correlates positively with light intensity (*r* = 0.62, *P* = 10^−14^) and negatively with absorption and *F*_v_/*F*_m_ (*r* = −0.79, *P* = 10^−28^ and *r* = −0.81, *P* = 3 × 10^−31^; Fig. 4b); GO terms are associated with plastids and proteolytic enzymes^42,43^ (FtsH and ClpP), recapitulating well-known ties of protein homeostasis and plastid maintenance (Extended Data Fig. 3). Indeed, yellow features five hubs that are homologous to genes coding for CLP proteases, critical for chloroplast protein homeostasis^44,45^, and hubs homologous to genes that orchestrate the coordination of transcriptional activity between chloroplasts and the nucleus (Fig. 5b); the latter includes homologues of (1) *pTAC6*, which is essential for plastid gene expression and thus chloroplast development in *Arabidopsis*^46^, and (2) a homologue of *GENOMES UNCOUPLED 2*, one of the foremost genes in the classical plastid–nucleus communication pathway^47^. Among the TF-coding genes in module yellow is a homologue of the bZIP light signalling master regulator *ELONGATED HYPOCOTYL 5* (ref. ^48^) (*HY5*). Module yellow is among those with the most consistency in similar modules across the analysed streptophyte co-expression networks and hubs (Fig. 5f,h), as exemplified by the GO term similarities between yellow and *Arabidopsis* module 18 (compare Fig. 5g and Extended Data Fig. 3) and the consistency of the plastid operational genes as hubs (Fig. 5h). Hence, hallmark genes for plastid operation and its integration into molecular cell physiology probably acted in concert since before the dawn of embryophytes.

### Of ancient signalling cascades and cell wall perturbance

Mitogen-activated protein kinases (MAPK) constitute environmental response pathways in all eukaryotes^49^. In land plants, several abiotic and biotic cues have been described to trigger MAPK-mediated signalling^50–53^. Genes coding for MAPK and phototropin kinases appear as hubs in module blue. Moreover, plant MAPK-based signalling is interwoven with wound response and brassinosteroid signalling^50^; the *MAPK* orthologue in *Zygnema* is also highly connected (Fig. 5h) and blue is similar to the kinase-rich module 17 of *Arabidopsis* (Fig. 5f). Stress often coincides with a perturbance of plant cell wall homeostasis. Module pink includes hubs for such wounding and cell wall-derived signals. This pairs with the GO term brassinosteroid signalling, which balances growth, cell wall homeostasis and stress in *Arabidopsis*^54,55^. Among the hubs in pink are homologues for (1) diverse receptor kinases known from *Arabidopsis* to sense alterations in cell wall integrity^56^ and (2) *EXORDIUM-like* (*EXL*; *Mesotaenium* has 12 *EXL* homologues), which integrates growth with environmental signalling^57^ (Fig. 5d). This pairs with genes coding for the COBRA family proteins being the most and third most connected hubs in the module. COBRA proteins are known to be involved in cell expansion and balancing pathogen response with growth^58–60^. It appears that *Mesotaenium* bears parts of a loop that senses physico-chemical perturbance of cell wall homeostasis; in land plants, these loops include brassinosteroid signalling^61^ and wiring of the core genes mentioned here are ancient, evident by the recurrent high connectivity of *EXL* and *COBL* homologues (Fig. 5h) throughout 600 million years of streptophyte evolution.

### LDs: a response pre-dating plant terrestrialization

**Fig. 6:**
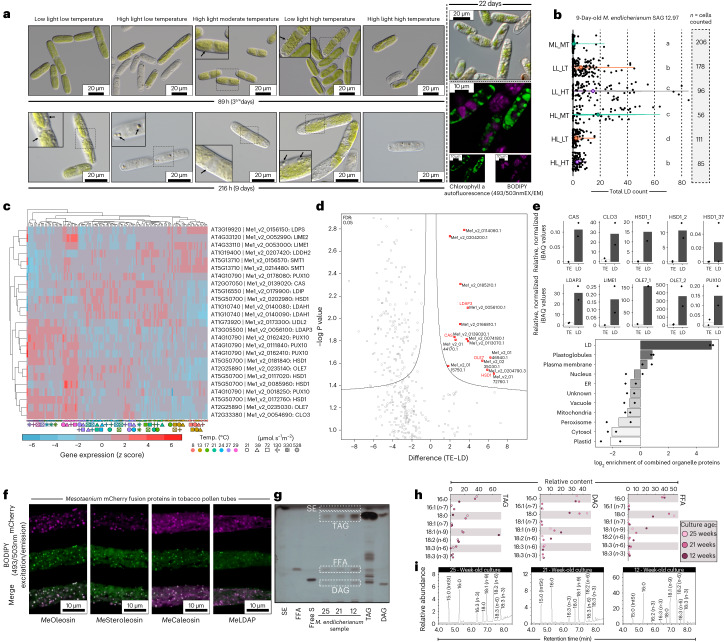
LDs accumulate in *Mesotaenium* upon changing environments. **a**, DIC and confocal micrographs of *Mesotaenium endlicherianum* SAG 12.97 cells accumulating LDs (arrows) upon exposure to different temperature/light conditions (abbreviations) of the gradient table for 89 h or 216 h. For confocal microscopy, algae were cultured independent of table conditions at 75 µmol photons m^−2^ s^−1^ and 22 °C for 22 days. LDs are visible as distinct globular structures and were stained with BODIPY (false-coloured green; 493 nm excitation, 503 nm emission); chlorophyll autofluorescence in false-coloured purple; for each condition, at least ten micrographs were taken, all showing similar phenotypes of the cells. **b**, Violin plots of LD quantification after 9 days of exposure to different environmental conditions; significance grouping (Mann–Whitney *U*) is based on *P* < 0.05; see also Supplementary Fig. 27. **c**, Heat map of row-scaled *z* scores of the expression of homologues for LD biogenesis and function (see also Supplementary Fig. 28). Conditions are displayed at the bottom as symbols in different colours; best *Arabidopsis* hits (via BLASTp) are shown on the right. **d**,**e**, Proteomic investigation into lipid-enriched phases extracted from *Mesotaenium*; note the enrichment in hallmark proteins of LDs. Volcano plot showing significantly (false discovery rate (FDR) <0.05) enriched *Mesotaenium* proteins in the lipid-enriched (LD) versus the TE (**d**). Bar plots show the relative, normalized iBAQ values for ten LD signature proteins detected in *Mesotaenium* (**e**). Bottom bar plot shows the log_2_ enrichment of proteins characteristic for subcellular compartments. LL, ML and HL, low, moderate and high light; LT, MT and HT, low, moderate and high temperature, respectively; ER, endoplasmic reticulum. **f**, LD proteins of *M.* *endlicherianum* localize to LDs in tobacco pollen tubes: cLSM images of transiently expressed proteins appended to mCherry in transiently transformed *N.* *tabacum* pollen tubes. LDs were stained with BODIPY 493/503; for each construct, the images are representative of at least nine micrographs of transformed pollen tubes per fusion construct. Scale bars, 10 µm. **g**,**h**, Lipid composition in *M.* *endlicherianum* LDs of 12- to 25-week-old cultures and standards for sterol esters (SE), FFA, free sterols (free S), TAG and DAG via analytical TLC (**g**) and preparative TLC followed by GC for profiling (**h**). **i**, Full lipid profiles assessed via GC. Source data

In land plants, lipid droplet (LD) formation and triacylglycerol (TAG) accumulation are common to many stress responses, including heat, cold and drought^62–66^. We observed that cells of *Mesotaenium* accumulated inclusions resembling LDs upon prolonged exposure to stress (Fig. 6a). Consistently, these globular structures were stained by BODIPY 493/503 (EM/EX), a common dye for lipid- and oil-rich compartments^67,68^. Under different temperature and light conditions, counts of LDs per cell showed significant differences (Fig. 6b and Supplementary Table 8). A *CGI-58* homologue is the tenth most connected hub in module green (Fig. 5c). CGI-58 is key to lipid homeostasis, causing, if perturbed, Chanarin–Dorfman syndrome in humans and LD overaccumulation in *Arabidopsis*^69,70^ (Fig. 5c); *CGI-58* is the 22nd most connected gene in *Arabidopsis* module 5 (Fig. 5h). Further, differential gene expression profiles pinpointed elevation of transcripts for characteristic LD protein homologues such as steroleosin (HSD1) and oleosin (OLE7) under high temperature and moderate light conditions (29 °C, 21–130 µmol photons m^−2^ s^−1^) and LD-associated protein (LDAP) and PUX10 under high temperature and light conditions (21–29 °C, 130–528 µmol photons m^−2^ s^−1^; Fig. 6c).

To scrutinize whether these structures are comparable to LDs of land plants, we performed subcellular fractionizations, obtained lipid-rich phases and subjected them to proteomics using liquid chromatography–mass spectrometry (LC–MS). We identified 739 proteins in the putative LD fraction and 1,574 proteins in the total extract (TE) (Supplementary Table 9). Of these, 14 were significantly enriched in the putative LD fraction (Fig. 6d) including hallmark LD proteins^71^ such as OLE, caleosin (CLO), HSD and LDAP (Fig. 6e). We confirmed the localization to LDs for these four proteins by transiently expressing mCherry-tagged variants in tobacco pollen tubes; mCherry clearly overlapped with BODIPY 493/503 fluorescence (Fig. 6f). Resembling LDs of seeds^71^, we found predominantly TAG in the lipid content of the LDs (Fig. 6g and Supplementary Fig. 27b); the lipid profiles of *Mesotaenium* LDs varied with age of the cultures (Fig. 6h,i). Overall, *Mesotaenium* responds to stress conditions by formation of LDs containing signature proteins typical of embryophytic LDs.

## Discussion

Owing to their plain morphology, Zygnematophyceae emerged as the unexpected closest algal relatives of land plants^4–7^. That said, the molecular programmes of Zygnematophyceae speak of their close relationship to land plants. These point to a conserved chassis that probably operated in the last common ancestor of land plants and algae, featuring the proposed action of various hallmark proteins (for example, PYL homologues^20^, GRAS family TFs^8^ and more) that were once considered land plant innovations. During plant terrestrialization, challenges did not come in isolation. The aim of this work was to define stress responses to temperature and irradiance combinations in a close algal relative of land plants. In the approach we have chosen, we made sure to capture both tolerable ranges of cues and those that go beyond tipping points, to allow for robust definition of where stress starts and to pinpointing molecular programmes whose expression dynamics follow the kinetics of their environmental trigger (for example, light intensity in the case of programmes for high light response); this included capturing the well-known double assault of low temperature and high light on the photosynthesis machinery. Building on the genomic resources for *Mesotaenium*, we have here delved into the molecular physiology and genetic programmes of this alga, revealing which programmes bear out when challenged with environmental cues.

Recent studies have proposed homology for the chassis of plastid–nucleus communication upon adverse environmental conditions between land plants and phragmoplastophytic streptophyte algae^20,72,73^. The GUN pathway probably has a conserved role in chloroplast transcription and streptophyte algal *GUN1* homologues can rescue chloroplast retrograde signalling of *Arabidopsis Atgun1* mutants^74^; the degree of evolutionary conservation in the retrograde signalling pathway across streptophytes remains obscure^74^. Signals from damaged chloroplasts inhibit *GLK1* expression in *Arabidopsis*^75^. The negative correlation of module blue (featuring Me*GLK*) with high light (leading to damaged chloroplasts) supports a role of *Me*GLK in operational retrograde signalling. Indeed, our comparative analyses revealed a consistency in plastidial integration on the basis of similar networks in land plants and *Zygnema* (Fig. 5f) and with regard to highly connected hub genes associated with ROS and plastid protein homeostasis (Fig. 5g,h). Altogether, these insights point to operational processes of the plastid of the closest relatives of land plants, governed by nuclear gene expression for dealing with light regimes and adjustment of photosynthetic performance. On balance, our data underscore that the wires between components in plastid–nucleus communication are probably shared across more than 600 million years of streptophyte evolution.

In land plants, the formation of LDs is known to occur under a variety of adverse environmental conditions^63,64,76^. Stress-dependent formation of LDs probably evolved before land plants came to be^24,77,78^, but their molecular underpinnings outside of land plants remain unclear. Here, we confirmed the identity of these *Mesotaenium* LDs using confocal microscopy, LD-specific staining and proteomics. Our comprehensive transcriptomic data illuminate co-expressed modules that might constitute a homologous programme for stress-dependent LDs that acted before plants conquered land.

## Methods

### Algal culturing and gradient table setup

We used the axenic and genome-sequenced *Mesotaenium endlicherianum* SAG 12.97 (ref. ^79^) from the Algal Culture Collection, Göttingen (SAG^80^). *Mesotaenium* was cultivated in C-medium^81^ for an average of 12 days in an aerated culture glass flask (SCHOTT) at 80 µmol photons m^−2^ s^−1^ (12h/12h light/dark cycle (light from 6 am to 6 pm, Central European winter time) at 18 °C). Before the experiment, cell density was analysed using a LUNA Automated Cell Counter (Logos Biosystems) and set to 2.03 × 10^7^ cells ml^−1^ (diluting with C-medium if needed; settings for cell counting: cell roundness, 60%; minimum size, 3 µm; maximum size, 60 µm), corresponding to Abs680nm = 0.33 (Epoch Microplate reader, BioTek Instruments). For the gradient table setup, the algal suspension was distributed across 504 wells (42 12-well plates (tissue culture testplates 12 no. 92412, TPP); 2.5 ml of culture per well). Plates were sealed with surgical Micropore tape (3M) to minimize evaporation. The 42 12-well plates were then placed on a table that generates a cross-gradient of temperature (8.6 ± 0.5 °C to 29.2 ± 0.5 °C on the *x* axis) and irradiance (21.0 ± 2.0 to 527.9 ± 14.0 µmol photons m^−2^ s^−1^ on the *y* axis) (Supplementary Table 1). The temperature gradient was generated using a custom-made table (Labio) equipped with true-daylight LEDs (sTube 2 W 120 ver 11:11, Snaggi) set to a 16 h/8 h light/dark cycle (light from 6 am to 10 pm, Central European winter time). *Mesotaenium* samples exposed to the 504 different conditions for 65 h (for sampling for RNA-seq and physiological measurements) and 89 h (for detailed light microscopy) on the gradient table. Condensed water at the top of the 12-well plates lids was removed three times in the 65 h by lightly tapping the lids twice.

### Algal culturing and gradient table setup (pre-experiments n1(I), n 1,2(II))

To assess optimal, stress and lethal culture conditions for *Mesotaenium endlicherianum* SAG 12.97 three pre-experiments were performed (n1(I), performed once, and n1,2(II), performed twice). We assessed *Mesotaenium* performance in Woods Hole Medium (WHM)^82,83^ and C-medium^81^ for an average of 23.6 days in aerated culture glass flasks (SCHOTT) at 80 µmol photons m^−2^ s^−1^ (12h/12h light/dark cycle (light from 6 am to 6 pm, Central European winter time) at 18 °C). Before the experiment, different cell densities were analysed using a microplate reader and adjusting the culture to Abs680nm 0.06 (n1(I) or 0.12 (n1,2(II)) (Epoch Microplate reader, BioTek Instruments). For the gradient table setup, the algal suspension was distributed across 504 wells (42 12-well plates (tissue culture testplates 12 no. 92412, TPP); 2.5 ml of culture per well). Plates were sealed with surgical Micropore tape (3M) to minimize evaporation. The 42 12-well plates were then placed on a table that generates a cross-gradient of temperature (for n1(I): 12.7 ± 1.0 °C to 34.0 ± 0.8 °C on the *x* axis; for n1,2(II): 8.6 ± 0.5 °C to 29.2 ± 0.5 °C on the *x* axis) and irradiance (21.0 ± 2.0 to 527.9 ± 14.0 µmol photons m^−2^ s^−1^ on the *y* axis) (Supplementary Table 1 and Supplementary Fig. 1a). The temperature gradient was generated using a custom-made table (Labio) equipped with true-daylight LEDs (sTube 2 W 120 ver 11:11, Snaggi) set to a 16 h/8 h light/dark cycle (light from 6 am to 10 pm, Central European winter time). *Mesotaenium* samples were exposed to the 504 different conditions either for 191 h (n1,2(II)) or 216 h (n1(I)) (for performing physiological measurements) and 216 h (n1,2(II)) (for absorption spectra measurements). Condensed water at the top of the 12-well plates lids was removed by lightly tapping the lids twice.

### Plate reader

In vivo absorbance at 480, 680 and 750 nm of all 42 plates was measured after 65 h exposition (4–6 h after light on) with an absorbance microplate reader Epoch (BioTek Instruments). Nine data points per well were analysed and averaged using Gen5 2.0 software (Biotek), resulting in 108 measurements per 12-well plate per wavelength. For downstream analyses, these values were averaged resulting in one value per 12-well plate per wavelength (Supplementary Fig. 1). After 89 h exposition, 16 plates were chosen from the prominent gradients (the four most extreme conditions in the corners and a cross of vibrant growth along the two gradients) for analysing a full absorption spectrum (300–900 nm) using the same setup (Supplementary Fig. 9 and Supplementary Table 10).

### Photophysiological measurements

For maximum quantum yield measurements (*F*_v_/*F*_m_) the maxi version of the IMAGING-PAM (ImagMAX/L, M-series, Walz) with an IMAG-K5 CCD camera, controlled with the ImagingWinGigE (V2.32) software, was used. The *Mesotaenium* cultures in the 12-well plates were dark adapted for 10–30 min before measuring. Before measurements, the lid was removed. For the *F*_v_/*F*_m_ measurement, a short saturation pulse (intensity 3) was applied. The measurement settings on the IMAGING-PAM were as follows: measuring light 1, gain 3, damping 2 and mean over area of interest was turned off. No special saturation pulse routine was applied to modify the signal-to-noise ratio of the chlorophyll fluorescence measurement.

### Statistical analysis of absorption and *F*_v_*/F*_m_ values and temperature/light cluster analysis

Statistical analysis of the absorption and the *F*_v_/*F*_m_ values was done using a Kruskal–Wallis test with a post hoc Fisher’s least significant difference test^84^ using R (version 4.1.3). *P* values were Bonferroni corrected and grouped into significant groups using R packages ‘agricolae’ version 1.3–5 and ‘dplyr’ version 1.0.9. For heat map generation of physiological values plotted against temperature or light, the R package ‘pheatmap’ version 1.0.12 was used. For cluster analysis, the R package ‘factoextra’ version 1.0.7 was used. Clusters were generated using the eclust function with clustering function ‘kmeans’ with the number of clusters set to six and for hierarchical clustering; ‘euclidean’ was used as the distance measure. Clusters were visualized with PCA in R.

### RNA extraction and sequencing

After absorption measurements, the 12-well plates were put back on the table to let cells adjust to the table conditions again for a minimum of 5 min before collecting them. For RNA extraction 0.4 ml was taken from every well of the 42 12-well plates on the table after pipetting the cells up and down twice to homogenize them. In total, 4.8 ml liquid culture was taken per condition on the table (that is, pooling 0.4 ml of each 12 wells per each of the 42 conditions). The samples were then centrifuged for 5 min at 20 °C and 4,200*g*. The supernatant was removed and the pellet was frozen at −80 °C. To extract RNA, the Spectrum Plant Total RNA Kit (STRN250-1KT, Sigma-Aldrich) was used according to the manufacturer’s instructions. For cell disruption, samples in lysis buffer were ultrasonicated for 1 min and vortexed. RNA samples were treated with DNAse I (EN0521; Thermo Fisher) and shipped on dry ice to Novogene where they were quality checked with a Bioanalyzer (Agilent Technologies). Libraries were built on the basis of total RNA using poly-T oligo-attached magnetic beads. Following fragmentation, synthesis of the first-strand complementary DNA was carried out using random hexamer primers and second-strand cDNA using dUTP, instead of dTTP. A directional size-selected library was built that included PCR-based amplification. Sequencing adaptors were 5′ adaptor: 5′-AGATCGGAAGAGCGTCGTGTAGGGAAAGAGTGTAGATCTCGGTGGTCGCCGTATCATT-3′ and 3′ adaptor: 5′-GATCGGAAGAGCACACGTCTGAACTCCAGTCACGGATGACTATCTCGTATGCCGTCTTCTGCTTG-3′. The library was sequenced on an Illumina NovaSeq 6000 platform and data were downloaded using wget (GNU Wget 1.14).

### Quality control of reads

We checked the quality of our raw reads via FastQC^84^ (v0.11.9) and summarized the results via MultiQC^85^ (v1.11). On the basis of these and the used adaptor sequence, we filtered and trimmed reads via Trimmomatic^86^ (v 0.36) with these parameters: (‘ILLUMINACLIP: novogene_adapter_sequences_Trimmomatic.fa:2:30:10:2:True LEADING:26 TRAILING:26 SLIDINGWINDOW:4:20 MINLEN:36′). We checked the quality of the trimmed reads with FastQC and MultiQC again.

### Genome annotation

The original annotation of *M.* *endlicherianum*^8^ had a lower number of genes compared with other Zygnematophyceae algae. We took advantage of our newly generated RNA-seq dataset to improve genome annotation. Trimmed reads were mapped via HISAT2 (ref. ^87^) and assembled via StringTie^87^. The StringTie results showed many novel isoforms as well as novel transcripts. We also used BUSCO V5 (ref. ^88^) to measure the completeness of the gene models in annotation V1 independent of StringTie. Although the gene prediction method used by BUSCO at the genome level is very efficient, it is not unexpected if it misses some proteins that were annotated in a genome via experimental data, based on bioinformatic methods and next-generation sequencing data, or ab initio-based gene prediction methods. Therefore, we expect that the BUSCO score based on the proteins of a gene model should be equal to or greater than the BUSCO score of the genome. When we compared the BUSCO score between the genome and protein sequences for *M.* *endlicherianum* with ‘viridiplantae.odb.10-2020-09-10’, we noticed that they show similar numbers (Supplementary Fig. 2). Therefore, we decided to re-annotate the genome of *M.* *endlicherianum* with our comprehensive RNA-seq datasets as well as public protein and genome sequences published for its close relatives.

We annotated the *M.* *endlicherianum* genome using REAT (v0.6.1). Various gene models were predicted based on different types of evidence and methods. The final gene models and annotation V2 were based on agreement with the experimental evidence. At the end, we tried to quantify ‘completeness’ and quality of the new annotation V2 and the old V1.

First, we used RNA-seq evidence with REAT’s ‘Transcriptome Workflow’ with HISAT2 (v2.2.1), Scallop^89^ (v0.10.5) and StringTie (v2.1.5). We also used Portcullis^90^ (v1.2.4) to identify genuine junctions based on short reads alignments. This workflow uses Mikado^91^ (v2.3.4) to identify the ‘best’ set of transcripts from multiple transcript assemblies.

Then, we used gene homology information from representative streptophytes in REAT’s ‘Homology Workflow’. SPALN^92,93^ (v2.4.7) was used to align representative protein sequences onto the *M.* *endlicherianum* genome. The representative dataset consisted on genome, gene models and protein sequences of *Anthoceros agrestis*^94^ (Oxford strain), *Arabidopsis thaliana*^95^, *Azolla filiculoides*^96^, *Chara braunii*^72^, *Chlorokybus melkonianii*^97^ (for naming, see ref. ^98^), *Chlamydomonas reinhardtii*^99^ (v5.6), *Klebsormidium nitens*^100^, *Mesostigma viride*^101^, *Marchantia polymorpha*^102^ (v6.1r1), *Penium margaritaceum*^11^, *Physcomitrium patens*^103^ (v3.3), *Selaginella moellendorffii*^104^ and *Spirogloea muscicola*^8^. We also used the junction file produced by Portcullis. Since there were no close relatives of *M.* *endlicherianum* on the SPALN species-specific parameter set, we used three different closest possibilities (Angiosp, Chlospec and MossWorts) and built three models. These alignments are filtered using a predefined set of criteria (compare code on GitHub) including exon length, intron length and internal stop codon, among others. The final gene models of V2 were prepared by Mikado.

Afterwards, we used REAT’s ‘Prediction Workflow’ to predict gene models ab initio and based on RNA-seq and homology evidence. This uses Augustus^105–107^ (v 3.4.0), SNAP^108^ (version 2006-07-28), Glimmer^109^ (v0.3.2) and CodingQuarry^110^ (v2.0), which generate different gene models as the raw material for EVidenceModeler^111^ (v1.1.1) that chooses the best set of exons and combine them in a gene model using weights (see GitHub) that could be adjusted for each sort of prediction and evidence. To include untranslated regions where possible, the EVidenceModeler output is then processed by Mikado using untranslated region-containing gene models from the transcriptome and homology workflows as inputs, as well as gene models classified by REAT as gold, silver and bronze based on their agreement with the set of protein sequences from other streptophyte genomes (streptophyte algae and land plants), transcriptome alignment, homology alignment and junctions. To train ab initio predictors, a user-defined number of models are randomly chosen in a user-defined ratio between mono-exonic (10%) and multi-exonic (90%). These models were chosen from best-classified models (gold and silver). For Augustus, we performed meta parameter optimization and train a model with kfold of 8. Beside ab initio predictions, we used Augustus to predict gene models with three different weights for each evidence type as suggested by REAT authors (compare code on GitHub).

At last, we used Minos^112^ (v1.8.0), which is gene model consolidation pipeline and produces external metrics based on DIAMOND^113^ (v0.9.34) ‘BLASTp/BLASTx’, Kallisto^114^ (v0.46.2) expression quantification, coding potential calculator^115^ (CPC2 v0.1) and BUSCO assessments. These metrics pass through Mikado in combination with various gene models produced with different methods (as mentioned above); Minos determines the best gene model for each region based on user-defined criteria (for details, see GitHub) and external metrics. Minos also puts a tag on each gene model to categorize them based on a user-defined threshold (we used default values) for sequence similarity coverage of homologues, BUSCO score, coding potential calculator score, transcript per million expression and transcript score into ‘high confidence’, ‘low confidence’ and ‘predicted genes’.

### Genome annotation assessment

We used two methods to compare the quality of the new gene model with the published one. We compared the BUSCO scores of the annotated protein sequences as well as genome sequence using the reference ‘viridiplantae.odb.10-2020-09-10’ dataset. We also used maker^116^ (v3.01.04) to calculate the AED^117^ to evaluate the agreement of the gene models with external evidences. Maker-P was used to build the *M.* *endlicherianum* gene model V1.

Further, we used the maker package to perform functional annotation via InterProScan and BLAST using the agat^118^ package (v0.9.2). Additionally, we performed a BLAST (v2.11.0+) search against *A.* *thaliana* protein sequences (Araport11) and reported the best hit for each sequence (Supplementary Table 11) and used eggNOG-mapper^119,120^ (v2.1.8) to perform functional annotation. We used DIAMOND^113^ (v2.0.15) with ultra-sensitive mode, with *e* value cut-off of 1e^−7^ and in an iterative manner. We used the protein sequences as our inputs and Viridiplantae (33090) as our taxonomy scope.

### RNA-seq analysis: pseudoalignment

To quantify gene expression, we used a Snakemake-managed pipeline (7.7.0) that hinged on Kallisto^114^ (v0.45.0). We indexed the transcriptome file with —kmer-size=31 parameter, and used —bootstrap-samples 100 and —rf-stranded to quantify gene expression based on pseudo-aligned reads. We used MultiQC to obtain an overview of alignment for each condition.

### Filtering, normalization, modelling mean–variance relationship and data exploration

Kallisto quantification files were imported into R (v4.2.0; tidyverse v1.3.1) with tximport^121^ (v1.24.0) to calculate the counts from abundance via ‘lengthScaledTPM’ based on our study design file (Supplementary Table 12). We used edgeR^122^ (v3.38.1) for filtering and trimmed mean of *M*-values normalization^123^ of the reads (genes with >1 count per million at log2 scale in at least three samples—the number of replicates—were kept). Then, we used the voom function from limma^124–127^ (v3.52.2) to model the mean–variance relationship. The normalized expression table on the log2 scale is available in Supplementary Table 13. We performed PCA based on the expression table output of voom and visualized the result with ggplot2 (ref. ^128^) (v3.3.6). We visualized the heat map of distance and Spearman correlation between all samples considering all genes via pheatmap (v1.0.12), and calculated clusters via the Euclidian method.

### RNA-seq analysis: WGCNA

We used the WGCNA^28,129^ package (v1.71) with the expression table produced by limma. We checked for and filtered out outliers as suggested by WGCNA authors (Supplementary Fig. 10). Then, we visualized the scale-free topology model fit (*R*^2^) against the soft thresholds (*β*) to pick a *β* for our network construction (Supplementary Fig. 11). We used signed network type and ‘bicor’ as our correlation function for WGCNA. On the basis of these results, we picked 16 as our soft threshold ‘*β*’. We experimentally chose a merging threshold of 0.25 after exploring different values from 0.2 to 0.4 and investigating the relationship between eigengenes and temperature, light intensity, *F*_v_*/F*_m_ and absorption (Supplementary Fig. 12). We built the gene co-expression network using a merging threshold of 0.25 for modules, maximum portion of outliers as 0.05 and minimum module size of 30. Then, we visualized the correlation between each module’s eigengene and temperature, light intensity, *F*_v_*/F*_m_ and absorption to identify which modules are more related to each treatment (Fig. 4c). We provided a table for all genes, their module assignment, inter- and intramodular connectivity, gene significance for temperature and light intensity, correlation with temperature and light intensity, and their module membership (that is, signed eigengene-based connectivity) (Supplementary Table 5). We also visualized the graphical representation of the topological overlap matrix (TOM) of our samples (Supplementary Fig. 13). To have a visual representation of gene expression in each module, we drew heat maps for each module via pheatmap (using the Euclidean method for calculating the distance and complete method clustering) (Supplementary Fig. 14). GO enrichment analysis was performed via the clusterProfiler package^130,131^ (v4.4.4) using the output of eggNOG-mapper and adjusted *P* value cut-off of 0.05 and *q* value cut-off of 0.05, considering only genes that are present in our GO term-to-gene table, which was expressed and passed filtering as our background gene universe (Supplementary Table 6). Determining the proper background gene list has major importance in enrichment analysis^132^.

To see how *A.* *thaliana*’s well-known genes in stress-response mechanisms (downloaded from the TAIR database via keyword search) were distributed across different modules, we performed BLASTp searches against the new *M.* *endlicherianum* annotated proteins. We visualized the distribution of these IDs for different stress-related keywords (Supplementary Fig. 15) and the expression of these genes across different samples via pheatmap (Supplementary Fig. 16). We defined as module hubs the top 20 genes (nodes) with the highest connectivity within each module (Supplementary Tables 5 and 14).

### Differential gene expression analysis

We performed differential gene expression analysis using the limma package. We divided samples into multiple groups as follows: low light intensity (21 and 39 µmol photons m^−2^ s^−1^), medium light intensity (72 and 129 µmol photons m^−2^ s^−1^), high light intensity (329 and 527 µmol photons m^−2^ s^−1^), low temperature (8 and 12 °C), medium temperature (17, 20 and 23 °C) and high temperature (26 and 29 °C; see grid/coloured table layout in Fig. 2). We performed all-against-all comparisons and an additional comparison of those samples from an *F*_v_/*F*_m_ < 0.5 versus low light intensity + medium temperature. We used duplicateCorrelation as suggested by Smyth et al.^133^ to consider technical replicates. We used cluster Profiler for GO enrichment analysis^131^ with an adjusted *P* value and *q* value cut-off of 0.01 and only genes that were expressed and passed filtering as our background universe. The heat map of gene expression profiles, dot plot and cnetplot of enriched GO terms for each comparison is available in Supplementary Table 14 and Supplementary Figs. 17–25).

### Phylogenetic analyses

We assembled a protein database based on the protein releases from the genomes of: *Anthoceros agrestis* BONN^94^, *Anthoceros punctatus*^94^, *Amborella trichopoda*^134^, *Arabidopsis thaliana*^135^, *Azolla filiculoides*^96^, *Bathycoccus prasinos*^136^, *Brassica oleracea*^137^, *Brassica rapa*^138^, *Brachypodium distachyon*^139^, *Capsella grandiflora*^140^, *Chara braunii*^72^, *Chlorokybus melkonianii*^97^ (for naming, see ref. ^98^), *Chlamydomonas reinhardtii*^99^, *Coccomyxa subellipsoidea*^141^, *Gnetum montanum*^142^, *Klebsormidium nitens*^100^, *Marchantia polymorpha*^143^, *Mesostigma viride*^97^, *Micromonas pusilla*^144^, *Micromonas* sp.^144^, *Oryza sativa*^145^, *Picea abies*^146^, *Physcomitrium patens*^103^, *Salvinia cucullata*^96^, *Selaginella moellendorffii*^104^, *Solanum lycopersicum*^147^, *Theobroma cacao*^148^, *Mesotaenium endlicherianum*^8^, *Ostreococcus lucimarinus*^149^, *Penium margaritaceum*^11^, *Spirogloea muscicola*^8^, *Ulva mutabilis*^150^, *Volvox carteri*^151^, *Isoetes taiwanensis*^152^ and *Ceratopteris richardii*^153^.

Homologues for proteins were detected using BLASTp with *Arabidopsis* and *Mesotaenium* proteins as query against the aforementioned proteins as database. Alignments were computed using MAFFT v7.490 (ref. ^154^). All phylogenies were computed with IQ-TREE^155^ multicore version 1.5.5; their respective best-fit model for protein evolution was determined using ModelFinder^156^ (integrated in IQ-TREE multicore version 1.5.5 for Linux 64-bit built 2 June 2017) according to Bayesian Information Criterion; and 1,000 ultrafast bootstrap^157^ pseudoreplicates were carried out and 100 non-parametric bootstrap^158^ pseudoreplicates for the LDAP phylogeny. We coloured phylogeny trees via ggtree (v3.9.0).

### DIC and confocal laser scanning microscopy

**Extended Data Fig. 4:**
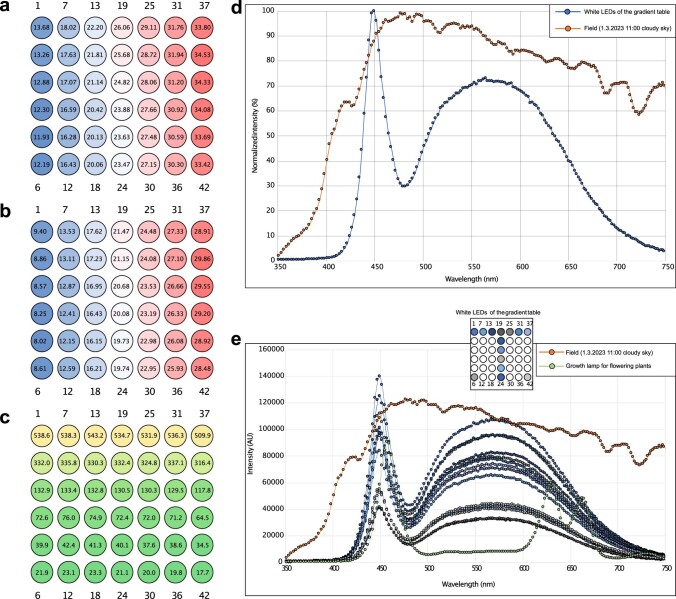
Pre-experimental setups: temperature conditions comparison, light intensities, and light spectra. (**a**) Temperature conditions for the first experimental setup (n1(I)) depicted by a blue to red color gradient (see also supplementary table ST 1.4). (**b**) Temperature conditions for the second and the final experimental setup (n1(II), n2(II), n1(III), n2(III), and n3(III)) depicted by a blue to red color gradient (see also supplementary table ST 1.2). (**c**) Light intensity/irradiance values depicted by a green to yellow color gradient (see also supplementary table ST 1.1). (**d**) Average light spectra of the gradient table (blue) assessed using SpectraPen (PSI, Brno, CZ) compared to a spectra assessed of natural sunlight (orange). (**e**) Light spectra of various plates of the gradient table (see overview) in various shades of blue assessed using SpectraPen (PSI, Brno, CZ) compared to a light spectrum assessed of natural sunlight (orange) and a light spectrum from a growth lamp used for flowering plants.

**Extended Data Fig. 5:**
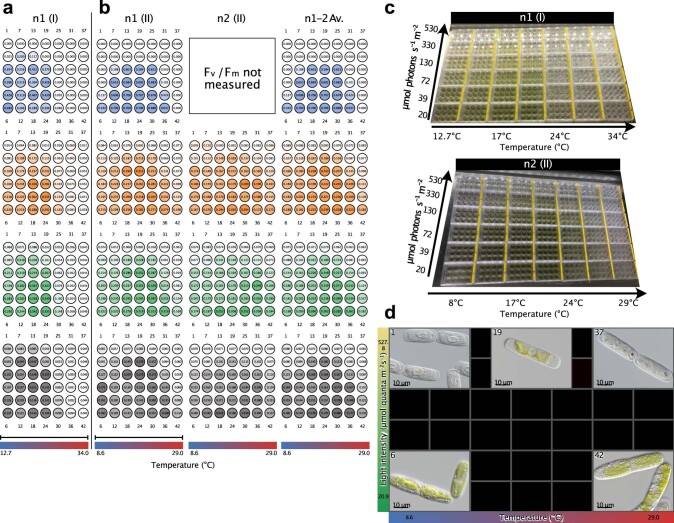
Pre-experimental setup II: Fv/Fm, absorption, and morphology. (**a**) Fv/Fm values (blue gradient) and absorption values (orange, green, grey color gradient, Colors indicate measured wavelength: orange gradients = Absorption measured at λ 480 nm, green gradients = Absorption measured at λ 680 nm, grey gradients = Absorption measured at λ 750 nm) of the first pre-experimental setup (n1(I)) with temperature settings ranging from 12.7–34 °C. (**b**) Fv/Fm and absorption values of second pre- experimental setup (n1(II) and n2(II)) and averaged values (n1-2 Av.) with new temperature settings ranging from 8.6 -29.0 °C. (**c**) Photographs of the pre-experimental setups n1(I) with temperature conditions ranging from 12.7–34 °C and n2(II) with temperature conditions ranging from 8.6 to 29.0 °C after incubation on the table for 216 h (n1(I)) or 191 h (n2(II)) respectively. The photograph of pre-experiment n2(I) is not shown. (**d**) Differential interference contrast (DIC) micrographs of SAG 12.97 cells (pre-experimental setup n1(II)) under most extreme environmental conditions (four corners: samples 1, 6, 37, and 42) as well as under high irradiance 527.8 µmol photons m^-2^ s^-1^ at 20.5 °C; for each well, at least 10 micrographs were taken, all showing similar phenotypes of the cells.

**Extended Data Fig. 6:**
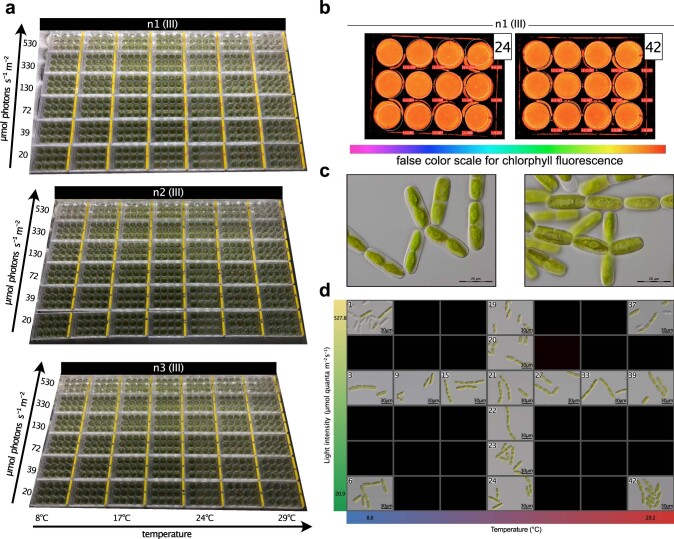
Main-experimental setup (n1,2,3 (III)): Morphology and growth. (**a**) Photographs of the main experimental setups n1, n2, and n3 (III) with temperature conditions ranging from 8.6 to 29.0 °C after incubation on the table for 65 h. (**b**) Fm measurements (maximal fluorescence) using IMAGING-PAM in various table conditions, legend on the right is a false color gradient indicating fluorescence intensity. (**c**) Differential interference contrast (DIC) micrographs of SAG12.97 cells grown on C-Medium (growth conditions see methods: growth conditions prior to exposure to environmental conditions); at least 10 micrographs were taken, all showing similar phenotypes of the cells. (**d**) Differential interference contrast (DIC) micrographs of SAG12.97 under most extreme environmental conditions (four corners: samples 1, 6, 37, and 42) as well as along an irradiance gradient at 21 °C (samples 19–24) and a temperature gradient at 130 µmol photons m^-2^ s^-1^ (samples 3, 9, 15, 21, 27, 33, and 39); for each well, at least 10 micrographs were taken, all showing similar phenotypes of the cells.

**Extended Data Fig. 7:**
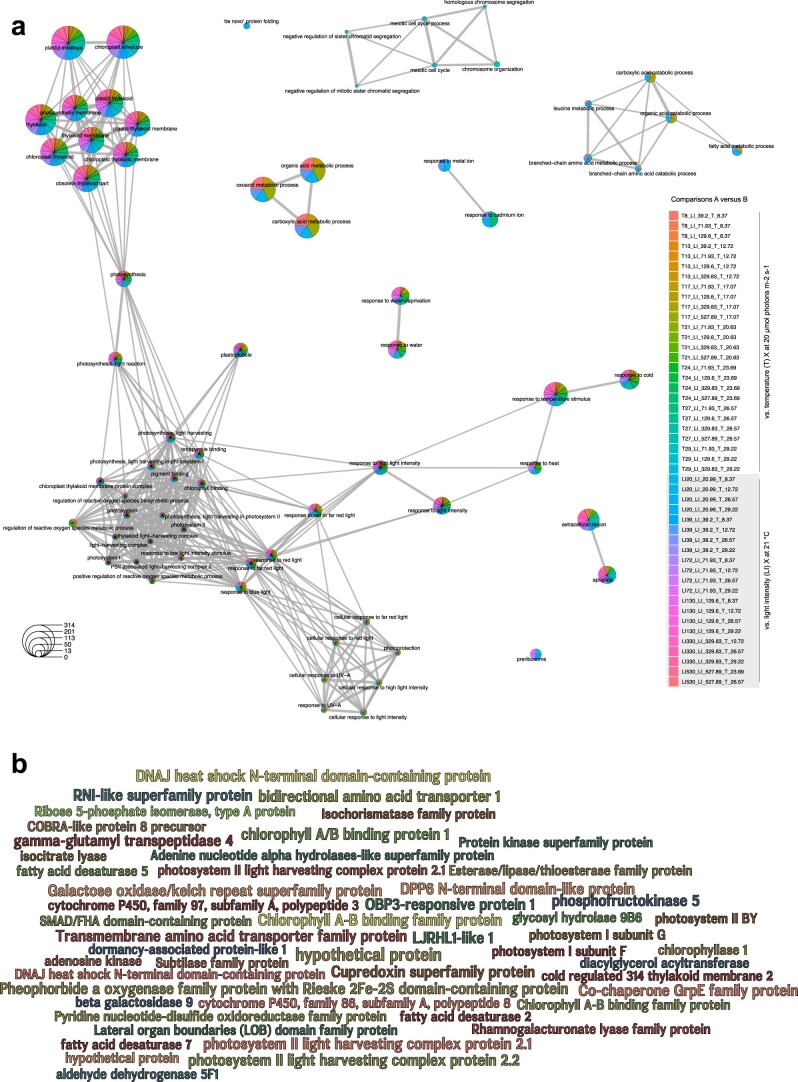
Differential gene expression comparisons highlight plastid-related responses. (**a**) Biological theme comparison of GO terms enriched in differential gene expression analyses in which one factor was always kept constant; the top 10 different connected graphs are shown. (**b**) Wordle of the 124 genes that showed significant regulation across multiple comparisons shown in main Fig. 2f,g and Extended Data Fig. 1; word size correspond to the number of comparisons in which a gene appeared. Color serves to increase the contrast between words.

**Extended Data Fig. 8:**
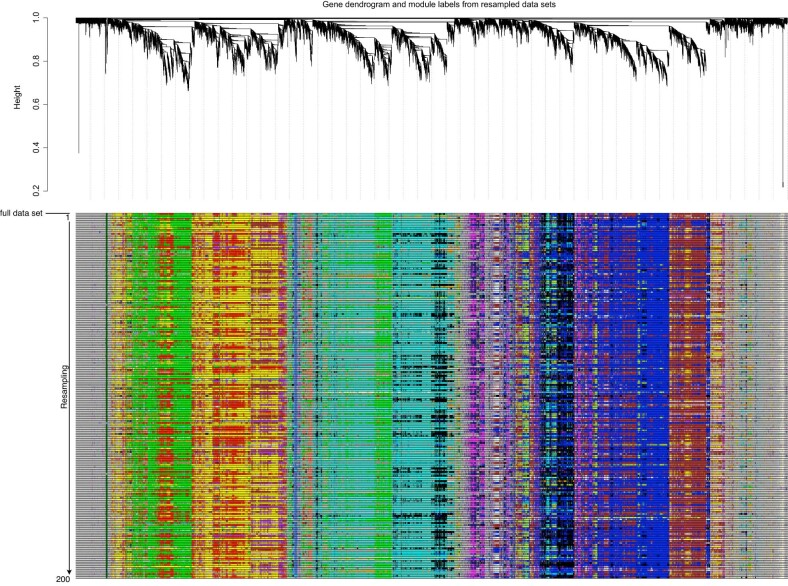
Module stability analysis. We performed 200 simulations by sampling from our input expression profiles under different conditions using the WGCNA package. Each row in the heat map represents a simulation and stable modules form a ‘column’ of similar color on the heat map.

Differential interference contrast (DIC) imaging was done for all replicates from the table with an Olympus BX-60 microscope (Olympus, Japan) with a ProgRes C14plus camera and the ProgRes CapturePro Software (version 2.9.01) (JENOPTIK AG). The morphology of chosen conditions (Fig. 1, Extended Data Figs. 4–6 and Supplementary Fig. 1) of *Mesotaenium* cells that were 89 h on the table was analysed.

For algae that were used for quantifying the abundance of LD per cell, a ZEISS Axioscope 7 microscope (Carl Zeiss) was used including the Zen software (Carl Zeiss). The LD count was carried out in Fiji^159^. For statistical analysis of the LD count data, we first used a Shapiro–Wilk test^160^ to assess normality and used Mann–Whitney *U* tests^161^ with R (version 3.6.1) accordingly.

Confocal laser scanning microscope was done on a Zeiss LSM780 (Carl Zeiss) set as in Müller et al.^162^. For the staining of the LD structures, we used the neutral lipid specific stain BODIPY 493/503 (EM/EX) (Merck). *Mesotaenium* cells were grown for 22 days on WHM medium at 70–80 µmol photons m^−2^ s^−1^ and 22 °C. These cells were ultrasonicated for 1 min with 1:500 BODIPY and incubated on a shaker for 5 min before visualization.

### LD isolation and proteomics

For LD isolation 23-day-old *Mesotaenium* cells grown on WHM medium at 70–80 µmol photons m^−2^ s^−1^ and 22 °C were homogenized using a Tenbroeck or potter homogenizer in LD isolation buffer (10 mM sodium phosphate buffer pH 7.5, 200 µM phenylmethylsulfonyl fluoride, 0.5 mM dithiobis(succinimidyl propionate) and 10 mM *N*-ethylmaleimide). The resulting centrifuged supernatant of a 100*g* spin for 1 min was considered as TE. After two further high-speed centrifugations (SW40 Ti for 1 h, 4 °C at 100,000*g*, TLA120 for 1 h at 100,000*g* and 4 °C) the floating fat pad was precipitated at −20 °C using 100% ethanol overnight. The precipitated pellet was washed with 80% ethanol twice, dried and then suspended in 6 M urea. Protein concentration was determined using the bicinchoninic acid assay. An in-gel sodium dodecyl sulphate gel digestion was done with trypsin adapted from Shevchenko et al.^163^. C18 Stage tip purification was done according to Rappsilber et al.^164,165^. Protein samples were analysed using LC–MS. For this, peptide samples were reconstituted in 20 µl LC–MS sample buffer (2% acetonitrile and 0.1% formic acid). Then, 2 µl of each sample were subjected to reverse-phase liquid chromatography for peptide separation using an RSLCnano UltiMate 3000 system (Thermo Fisher Scientific). Peptides were loaded on an Acclaim PepMap 100 pre-column (100 µm × 2 cm, C18, 5 µm, 100 Å; Thermo Fisher Scientific) with 0.07% trifluoroacetic acid at a flow rate of 20 µl min^−1^ for 3 min. Analytical separation of peptides was done on an Acclaim PepMap RSLC column (75 µm × 50 cm, C18, 2 µm, 100 Å; Thermo Fisher Scientific) at a flow rate of 300 nl min^−1^. The solvent composition was gradually changed within 94 min from 96% solvent A (0.1% formic acid) and 4% solvent B (80% acetonitrile and 0.1% formic acid) to 10% solvent B within 2 min, to 30% solvent B within the next 58 min, to 45% solvent B within the following 22 min and to 90% solvent B within the last 12 min of the gradient. All solvents and acids had Optima grade for LC–MS (Thermo Fisher Scientific). Eluting peptides were on-line ionized by nano-electrospray using the Nanospray Flex Ion Source (Thermo Fisher Scientific) at 1.5 kV (liquid junction) and transferred into a Q Exactive HF mass spectrometer (Thermo Fisher Scientific). Full scans in a mass range of 300–1,650 *m*/*z* were recorded at a resolution of 30,000 followed by data-dependent top ten higher energy collisional dissociation fragmentation at a resolution of 15,000 (dynamic exclusion enabled). LC–MS method programming and data acquisition was performed with the XCalibur 4.0 software (Thermo Fisher Scientific). Afterwards, the raw proteome data were analysed using Max Quant software^166^ version 1.6.2.10. The database for this analysis was our new V2 gene model data. The data were then further processed by the Perseus (1.6.2.2) software^166,167^.

### Lipid analysis of LDs

LDs (200–300 µl) were extracted with 10 ml of methanol/chloroform/formic acid (20:10:1, vol/vol/vol), 5 ml of 0.2 M phosphoric acid and 1 M potassium chloride^168^. After vortexing and centrifugation at 50*g* for 2 min, the lower chloroform phases were dried under streaming nitrogen and redissolved in chloroform/methanol (2:1, vol/vol). For analytical analysis, one-fifth of the lipid extracts were spotted on a thin layer chromatography (TLC) silica plate (TLC Silica gel 60, 20 × 20 cm, Merck KGaG) and separated with petroleum ether/diethyl ether/acetic acid (70:30:0.5, vol/vol/vol)^169^. The lipid composition was identified after incubation in copper sulphate solution (0.4 M CuSO_4_ in 6.8 % (vol/vol) phosphoric acid) and heating at 180 °C. For preparative analysis, half of the lipid extracts were additionally separated by TLC. After development, the lipid spots were visualized after spraying with 0.05% (wt/vol) primuline in 80% (vol/vol) acetone. The silica gel spots containing TAG, diacylglycerol (DAG) and free fatty acids (FFA) were used for preparation of fatty acid methyl esters as already described^170^ with some modifications. For acidic hydrolysis, 1 ml of methanol/toluene (2:1, vol/vol) containing 2.75% (vol/vol) sulphuric acid (95–97%) and 2% (vol/vol) dimethoxypropane was added to the scraped silica gel. For quantification, 1 µg of tripentadecanoate was added and the samples were incubated for 1 h at 80 °C. To extract the resulting fatty acid methyl esters, 1.5 ml of saturated aqueous sodium chloride solution and 1.2 ml of hexane were added and centrifuged at 450*g* for 10 min. The hexane phase was dried under streaming nitrogen and redissolved in 10 µl acetonitrile. Gas chromatography (GC) analysis was performed with an Agilent (Waldbronn, Germany) 6890 gas chromatograph fitted with a capillary DB-23 column (30 m × 0.25 mm; 0.25 µm coating thickness; J&W Scientific, Agilent) modified from Hornung et al.^171^. Helium was used as carrier gas at a flow rate of 1 ml min^−1^. The temperature gradient was 150 °C for 1 min, 150–200 °C at 4 K min^−1^, 200–250 °C at 5 K min^−1^ and 250 °C for 6 min. The peak area was collected with the ChemStation software (Agilent). From the absolute fatty acid contents, relative fatty acid profiles for TAG, DAG and FFA were calculated.

### Pollen tube transformation and microscopy

Coding sequences for *Mesotaenium* homologues salient to LD biology were *MeCaleosin*f 5′-GGGGACAAGTTTGTACAAAAAAGCAGGCTCATGTCGAAGCTCAGTCTTGCC-3′, *MeCaleosin*r 5′-GGGGACCACTTTGTACAAGAAAGCTGGGTCAGACTGCTTCTTCCTCTGCTT-3′, *MeLDAP*f 5′-GGGGACAAGTTTGTACAAAAAAGCAGGCTCATGGCCGAAAGTCAGGGCCC-3′, *MeLDAP*r 5′-GGGGACCACTTTGTACAAGAAAGCTGGGTCCGACTTCTTGAGGGCGTCGGC-3′, *MeSteroleosin*f 5′-GGGGACAAGTTTGTACAAAAAAGCAGGCTCATGGGGTTACTTAATGCCCTTGC-3′, *MeSteroleosin*r 5′-GGGGACCACTTTGTACAAGAAAGCTGGGTCGCCATTGGACTTGACGAGGG-3′, *MeOleosin*f 5′-GGGGACAAGTTTGTACAAAAAAGCAGGCTCATGCCTCAGGATCAGCAGCAAG-3′, and *MeOleosin*r 5′-GGGGACCACTTTGTACAAGAAAGCTGGGTCCTTCCTCTCCTTCTCAACCTTGT-3′. Constructs for expression in pollen tubes were cloned into the pLatMCC-GW vector using the fast Gateway method as described previously^162^. Pollen transformation, pollen tube growth and fixation were also performed according to this protocol. LDs were stained with BODIPY 493/503 at a final concentration of 1.3 µg ml^−1^. Microscopy images of transformed tobacco pollen tubes were acquired using an LSM 980 confocal laser scanning microscope using the objective C-Apochromat 40×/1.20 W Korr (both Carl Zeiss). mCherry was excited at 561 nm and detected at 600–640 nm. BODIPY 493/503 was excited at 488 nm and detected at 490–535 nm. In both cases, the major beam splitter MBS 488/561 was used. Both channels were recorded independently using the line mode.

### Comparative evolutionary analyses

To perform comparative evolutionary analyses among *M.* *endlicherianum*, other streptophyte algae and embryophytes, we used two separate workflows based on one criterion: the availability of at least 15 raw RNA-seq samples for a given species challenged with abiotic stresses and control conditions. This is the minimum requirement to build a co-expression network using the WGCNA package. If a species passed this criterion, we used them in two approaches; all results from the comparative analyses can be found in Supplementary Table 15.

Approach 1: to compare co-expression networks computed based on control and abiotic stress samples, we first used Orthofinder and protein sequences of *A.* *agrestis*^94^, *A.* *filiculoides*^96^, *A.* *thaliana*^135^, *B.* *distachyon*^139^, *C.* *braunii*^72^, *Closterium sp. NIES 67* (ref. ^10^), *C.* *melkonianii*^97^, *C.* *reinhardtii*^99^, *K.* *nitens*^100^, *M.* *endlicherianum*^8^, *M.* *polymorpha*^102^, *M.* *viride*^97^, *O.* *sativa*^145^, *P.* *margaritaceum*^11^, *P.* *patens*^103^, *S.* *lycopersicum*^147^, *S.* *moellendorffii*^104^, *S.* *muscicola*^8^, *Z.* *mays*^172^ and *Zygnema circumcarinatum*^9^ as well as a species cladogram to find phylogenetic HOGs using these parameters: -S mmseqs -M msa -A mafft -s species_tree.txt -y. For *A.* *thaliana*, we downloaded a gene-GO table from arabidopsis.org. For *P.* *patens*, *M.* *polymorpha* and *Zygnema 1b*, we used eggNOG-mapper and their protein sequences to create a gene-GO table using these parameters: -m diamond -dmnd_iterate yes -evalue 1e-10 -sensmode ultra-sensitive -tax_scope 33090. We downloaded raw RNA-seq reads for *A.* *thaliana*^173–177^, *P.* *patens*^178^ and accessions PRJNA277025 and PRJNA192876, *M.* *polymorpha*^179–181^ and *Zygnema 1b* (ref. ^9^) from the National Center for Biotechnology Information (NCBI). We followed the same quantification as *Mesotaenium* for each species here. In short, we used FastQC, MultiQC and Trimmomatic to check the quality of each read and filter and trim the raw reads. Then, we used Kallisto to pseudoalign the reads to the transcriptome of that species. Then, we imported gene counts for each species into R and performed similar exploratory analyses to *Mesotaenium* for each species. An additional layer of analysis here was to check for batch effect when we looked at all samples from different sources for a species. We used hierarchical clustering and PCA to pick the best expression profile from (i) uncorrected, (ii) batch-corrected as a covariate using limma, and (iii) batch-corrected using ComBat-seq^182^ to adjust for batch effects (if there were any). There is a debate in the community about which method is the best practice; therefore, we did all for every species and picked the best (less confounding effect between batches and maximum similarity between similar conditions) for each species. Then, we used the expression profile and built a signed co-expression network using the WGCNA package for each species. We followed the same procedure as *Mesotaenium*. We performed a GO enrichment analysis for each module in the co-expression networks. Then, we used the Orthofinder-based orthogroups to find genes that have a counterpart in *Mesotaenium* for each species and then we calculated the Jaccard similarity and dissimilarity between each *Mesotaenium* modules and each module of *A.* *thaliana*, *P.* *patens*, *M.* *polymorpha* and *Zygnema circumcarinatum* SAG698-1b. For each module in these co-expression networks, we looked for the connectivity of genes that share a HOG with *Mesotaenium* hubs.

Approach 2: to determine the shared DEGs under abiotic stresses across streptophyte algae, we first downloaded raw RNA-seq reads from NCBI as follows: (1) *Mougeotia*^24,25^ sp. MZCH240 and *S.* *pratensis* MZCH10213 (ref. ^24^), (2) *M.* *viride*, *C.* *cerffii*, *K.* *flaccidum*, *C.* *globularis*, *C.* *scutata*, *Zygnema ‘cylindricum’*^20^ *SAG698-1a*, (3) *Zygnema* sp.^23^ SAG2419, (4) *S.* *pratensis*^22^ UTEX92 and (5) *Z.* *circumcarinatum*^9^. If it was possible, we also obtained the transcriptome or genome file for each species. Then, we used Orthofinder and protein sequences of *Mesotaenium* and the protein sequences of these species as well as a species cladogram to find phylogenetic hierarchical orthogroups using these parameters: -S mmseqs -M msa -A mafft -s species_tree.txt -y. For species for which only the transcriptome was available, we used TransDecoder (v5.7.0) using TransDecoder.LongOrfs and TransDecoder.Predict scripts to get a protein-coding sequence for our Orthofinder run. For those that we did not have a transcriptome, we built one using Trinity (v2.15.1) (ref. ^183^) and the settings -seqType fq –trimmomatic. We followed the same quantification steps as for *Mesotaenium* and workflow A to pseudoalign reads to the transcriptome. Then, we followed similar steps to *Mesotaenium* to calculate DEGs for each species. Finally, we compared these DEGs with DEGs in *Mesotaenium* using HOGs from Orthofinder run in this workflow. We used BioNERO package^184^ to aggregate log_2_(fold change) values for each gene in each species to the corresponding HOGs and then used cluster Profiler to perform GO enrichment analyses and visualized the heat maps. In all comparisons, we considered adjusted *P* values <0.05 as significant enrichment.

### TEs

We used InterProScan^185^ (v5.59-91.0) on all predicted proteins in *Mesotaenium endlicherianum* V2 and filtered the results for transposon-related domains. This resulted in 6,186 entries in 1,748 unique gene IDs, among which only 96 were expressed in our RNA-seq data (that is, passing an expression cut-off of at least 1 count per million in at least three samples); all results are presented in Supplementary Table 4b).

### Reporting summary

Further information on research design is available in the Nature Portfolio Reporting Summary linked to this article.

## Supplementary information

Supplementary Information Overview of supplementary contents and Supplementary Figs. 1–28.

Reporting Summary

Supplementary Tables Supplementary Tables 1–15.

## Data Availability

All RNA-seq reads have been uploaded to the NCBI Sequence Read Archive and can be accessed under BioProject PRJNA832564 and Sequence Read Archive accessions SRR18936040 to SRR18936170. Furthermore, data can be interactively explored at https://mesotaenium.uni-goettingen.de and proteomic data have been uploaded to EMBL-EBI PRIDE (accession PXD037847). On Zenodo, we have deposited (1) raw light and confocal micrographs generated, for example, for LD assessment in *Mesotaenium* and pollen tubes 10.5281/zenodo.7921367 and (2) raw and visualized phylogenetic data 10.5281/zenodo.7950653. The additional previously published RNA-seq datasets that were used for comparisons are: (1) *A.* *thaliana*: SRR2302908 to SRR2302919, ERR754084, ERR754066, ERR754077, ERR754069, ERR754087, ERR754064, ERR754059, SRR7659142, SRR7659143, SRR7659144, SRR7659145 to SRR7659150, SRR5197904, to SRR5197909; (2) *M.* *polymorpha*: SRR12076853, SRR12076855, SRR12076857, SRR12076859, SRR12076861, SRR12076863, SRR12076865, SRR12076867, SRR12076869, SRR12076871, SRR12076873, SRR12076875, SRR12076877, SRR12076879, SRR12076917 to SRR12076925, SRR15186078 to SRR15186125, DRR093991 to DRR093996; (3) *P.* *patens*: SRR1824306 to SRR1824320, SRR10235460 to SRR10235483, SRR787291, SRR787292, SRR787293, SRR787294, SRR787295; (4) *Z.* *circumcarinatum* SAG698-1b: SRR24939299, SRR24940177, SRR24909175, SRR24757807, SRR24757829, SRR24757830, SRR24757831, SRR24205691 to SRR24205702, SRR24286545 to SRR24286562, SRR24576622, SRR24576623, SRR24385702, SRR24450996, SRR24450997, SRR24451196, SRR24480449, SRR24707416, SRR24707417, SRR24952091, SRR21891679 to SRR21891705; (5) *C.* *cerffii* (at the time, *C.* *atmophyticus*, see ref. ^98^): SRR5949009, SRR5949013 to SRR5949016, SRR5949027 to SRR5949030; (6) *C.* *scutata*: SRR5948993, SRR5948995 to SRR5948998, SRR5949001, SRR5949004, SRR5949005, SRR5949007; (7) *K.* *flaccidum*: SRR5949010, SRR5949011, SRR5949012, SRR5990072 to SRR5990080; (8) *M.* *viride*: SRR5949021 to SRR5949026; (9) *Mougeotia* sp. MZCH240: SRR9083681, SRR9083682, SRR9083688, SRR9083692 to SRR9083701; (10) *S.* *pratensis* MZCH10213: SRR9083685, SRR9083686, SRR9083687, SRR9083689, SRR9083690, SRR9083696; (11) *S.* *pratensis* UTEX928: SRR4018077 to SRR4018100; (12) *Z.* *circumcarinatum* SAG698-1a: SRR5948999, SRR5949000, SRR5949002, SRR5949003, SRR5949006, SRR5949008, SRR5949017, SRR5949018; and (13) *Z.* *circumcarinatum* SAG2419: SRR6047298, SRR6047299, SRR6047302 to SRR6047305. Source data are provided with this paper.

## Source data

Source Data Fig. 6 Whole scan of the TLC, shown in Fig. 6g.

## Code availability

Codes and data used for genome re-annotation, WGCNA and differential gene expression analysis are available on our GitHub page https://github.com/deVries-lab/Response_to_a_gradient_of_environmental_cues_in_mesotaenium_endlicherianum.
