# Supplementary material for: Environmental gradients reveal stress hubs pre-dating plant terrestrialization
Source: Nat Plants. 2023 Aug 28;9(9):1419–38. doi: 10.1038/s41477-023-01491-0 (PMC10505561; doi:10.1038/s41477-023-01491-0)
Supplement: Supplementary file 1 — Overview of supplementary contents and Supplementary Figs. 1–28. [file 41477_2023_1491_MOESM1_ESM.pdf]

nature plants

Article https://doi.org/10.1038/s41477-023-01491-0

Environmental gradients reveal stress hubs pre-dating plant terrestrialization

In the format provided by the authors and unedited

OVERVIEW OF SUPPLEMENTAL INFORMATION

SUPPLEMENTAL FIGURES

Supplemental Figure S1A: Main-experimental setup (n1,2,3 (III)): Fv/Fm, absorption, correlation.
Supplemental Figure S1B: Regression analysis.
Supplemental Figure 2. BUSCO comparison between genome, protein sequences V1, protein sequences V2
Supplemental Figure 3. Cumulative fraction of annotation vs AED plot for gene model V1 and V2
Supplemental Figure 4. Module membership versus Gene Significance for genes in different modules with respect to F_v/F_m
Supplemental Figure 5. Module membership versus Gene Significance for genes in different modules with respect to Temperature
Supplemental Figure 6. Module membership versus Gene Significance for genes in different modules with respect to light intensity
Supplemental Figure 7. Heatmap of the correlation between module eigengenes and light intensity, temperature, absorption, replicate, and F_v/F_m as well as student test p-value
Supplemental Figure 8. The GLK alignment
Supplemental Figure 9. Absorption spectra of all replicates at chosen conditions.
Supplemental Figure 10. Sample dendrogram and trait heatmap to identify outliers for WGCNA.
Supplemental Figure 11. Picking a soft threshold for WGCNA based on scale independence and Mean connectivity
Supplemental Figure 12. Clustering of different modules and traits based for identifying a merging threshold
Supplemental Figure 13. The graphical representation of the topological overlap matrix
Supplemental Figure 14A. Heatmap of gene expression Z-score values for each module.
Supplemental Figure 14B. GO enrichment analysis for *A. thaliana* co-expression modules.
Supplemental Figure 14C. GO enrichment analysis for *M. polymorpha* co-expression modules.
Supplemental Figure 14D. GO enrichment analysis for *P. patens* co-expression modules.
Supplemental Figure 14E. GO enrichment analysis for *Z. circumcarinatum* SAG 698-1b co-expression modules.
Supplemental Figure 14F. GO enrichment analysis for *M. endlicherianum* co-expression modules.
Supplemental Figure 15. Distribution of best blast hit of *A. thaliana* stress response genes among WGCNA modules
Supplemental Figure 16. Heatmap of best blast hit of *A. thaliana* stress response genes in *M. endlicherianum* across different growth conditions
Supplemental Figure 17. Dotplot, cnetplot and heatmaps of DEGs comparing FvFm control vs stress
Supplemental Figure 18. Dotplot, cnetplot and heatmaps of DEGs comparing HLI_HT vs LLI_MT
Supplemental Figure 19. Dotplot, cnetplot and heatmaps of DEGs comparing MLI_HT vs LLI_MT
Supplemental Figure 20. Dotplot, cnetplot and heatmaps of DEGs comparing LLI_HT vs LLI_MT
Supplemental Figure 21. Dotplot, cnetplot and heatmaps of DEGs comparing HLI_MT vs LLI_MT
Supplemental Figure 22. Dotplot, cnetplot and heatmaps of DEGs comparing MLI_MT vs LLI_MT
Supplemental Figure 23. Dotplot, cnetplot and heatmaps of DEGs comparing HLI_LT vs LLI_MT

Supplemental Figure 24. Dotplot, cnetplot and heatmaps of DEGs comparing MLI_LT vs LLI_MT
Supplemental Figure 25. Dotplot, cnetplot and heatmaps of DEGs comparing MLI_LT vs LLI_MT
Supplemental Figure 26A. Supplementary Figure S26A: Pairwise GO enrichment analysis of HOGs that are regulated in 10 streptophyte algae and *Mesotaenium*.
Supplemental Figure 26B. Fully-labeled phylogenies of hub genes.
Supplemental Figure 27. Lipid droplet count setup 2.
Supplemental Figure 28. LDAP phylogeny.

SUPPLEMENTAL TABLES

Supplemental Table 1. Temperature and light intensity measurements of all 504 coordinates on the gradient table.
Supplemental Table 2. All 504 F_v/F_m and absorption measurements of all replicates.
Supplemental Table 3. Number of genes and transcripts in gene model V2
Supplemental Table 4. The general stats of raw reads, trimmed reads, and pseudoalignment
Supplemental Table 5. Summary of WGCNA Results
Supplemental Table 6. The results of GO-enrichment analysis for all modules of WGCNA
Supplemental Table 7. The list of top20 hubs for each module.
Supplemental Table 8. Counts of lipid droplets in micrographs.
Supplemental Table 9. Full proteomic results, showing Mesotaenium gene model V2 identifiers, Arabidopsis gene identifiers, and IBAQ values.
Supplemental Table 10. All data on absorption spectra of all replicates at chosen conditions.
Supplemental Table 11. Best blast hit of *M. endlicherianum* gene model against *A. thaliana* (Araport11)
Supplemental Table 12. Study design file used for RNASeq analysis
Supplemental Table 13. The CPM normalized expression table on the log2 scale
Supplemental Table 14. The GO-enrichment results of 9 pairwise comparisons
Supplemental Table 15. Comparative analyses across 600 million years of streptophyte evolution

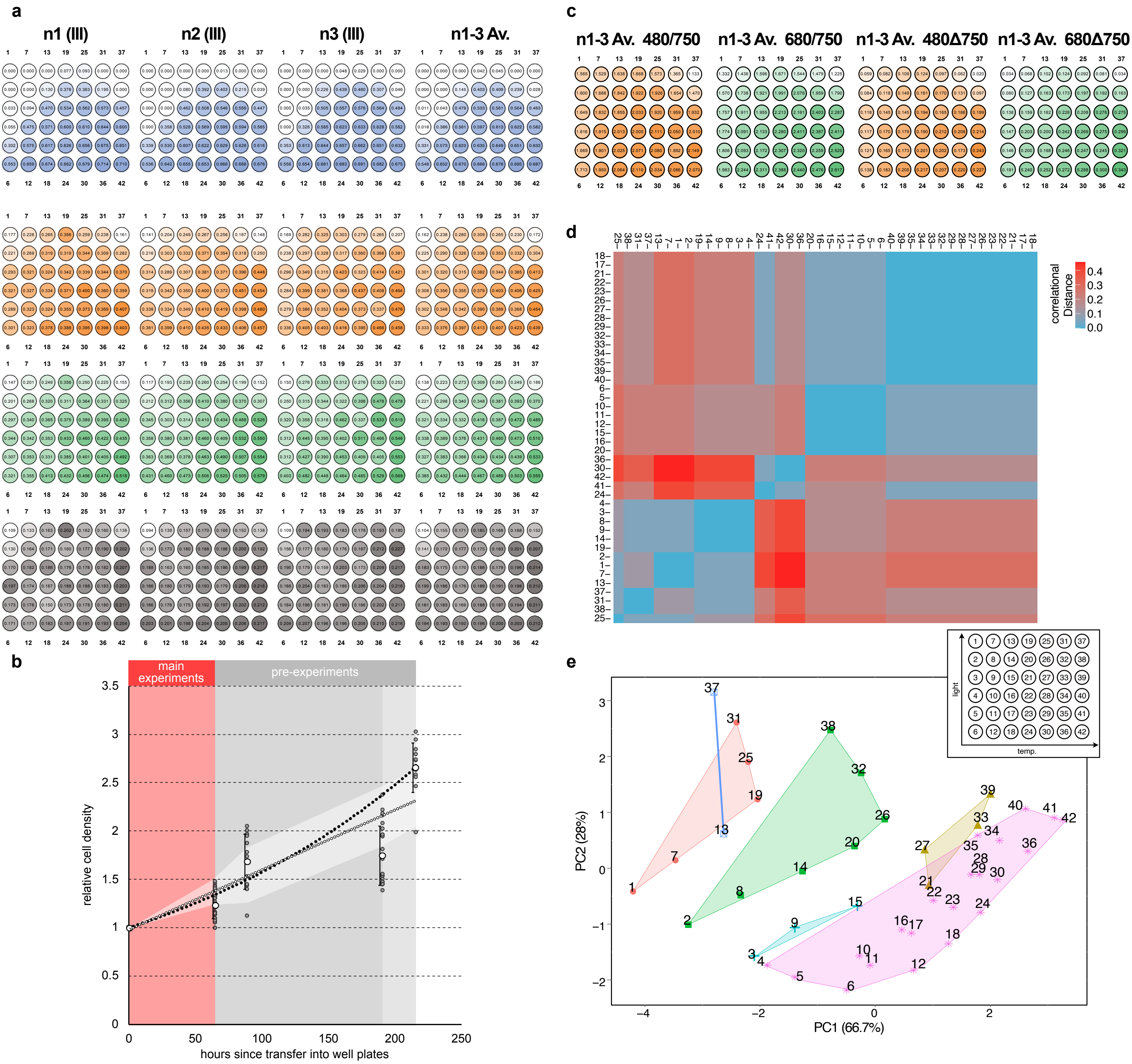

Supplementary Figure S1A: Main-experimental setup (n1,2,3 (III)): F*v*/F*m*, absorption, correlation. (a) F*v*/F*m* values of all biological replicates (n1-n3) as well as the averaged values (n1-3 Av.). Blue color gradient corresponds to increase/decrease of values. Numbers shown at the top/bottom indicate 12-well plate numbering on the gradient table. Absorption values of all biological replicates (n1,2,3 (III)) as well as the averaged values (n1-3 Av.). Color gradient corresponds to increase/decrease of values. Colors indicate measured wavelength: orange gradients = Absorption measured at λ 480 nm, green gradients = Absorption measured at λ 680 nm, grey gradients = Absorption measured at λ 750 nm. Numbers shown at the top/ bottom indicate 12-well plate numbering on the gradient table. **(b)** Scatter plot and trend lines of relative cell density (y-axis) absorption measured at 750 nm for all performed experimental setups (main experiments (n1-3(III)) in red, pre-experiments (n1(I), n1,2(II), n1-3(III)) in grey). On the x-axis the hours of growth of the *Mesotaenium* cultures in well plates on the gradient table is shown. **(c)** Calculation of 480/680/750 nm ratio as well as 480Δ750 and 680Δ750 nm of the averaged absorption values of n1,2,3 (III). **(d)** Visualization of correlational distances between light and temperature conditions and physiological values (F*v*/F*m*, 480Δ750 and 680Δ750 nm) via heatmap, from blue, zero distance, to red, furthest distance. Correlation-based distance was calculated using "spearman" method. **(e)** Principal component analysis (PCA) showing the correlation of light and temperature conditions to the averaged (n1-3 Av.) physiological values (F*v*/F*m*, 480Δ750 and 680Δ750 nm).

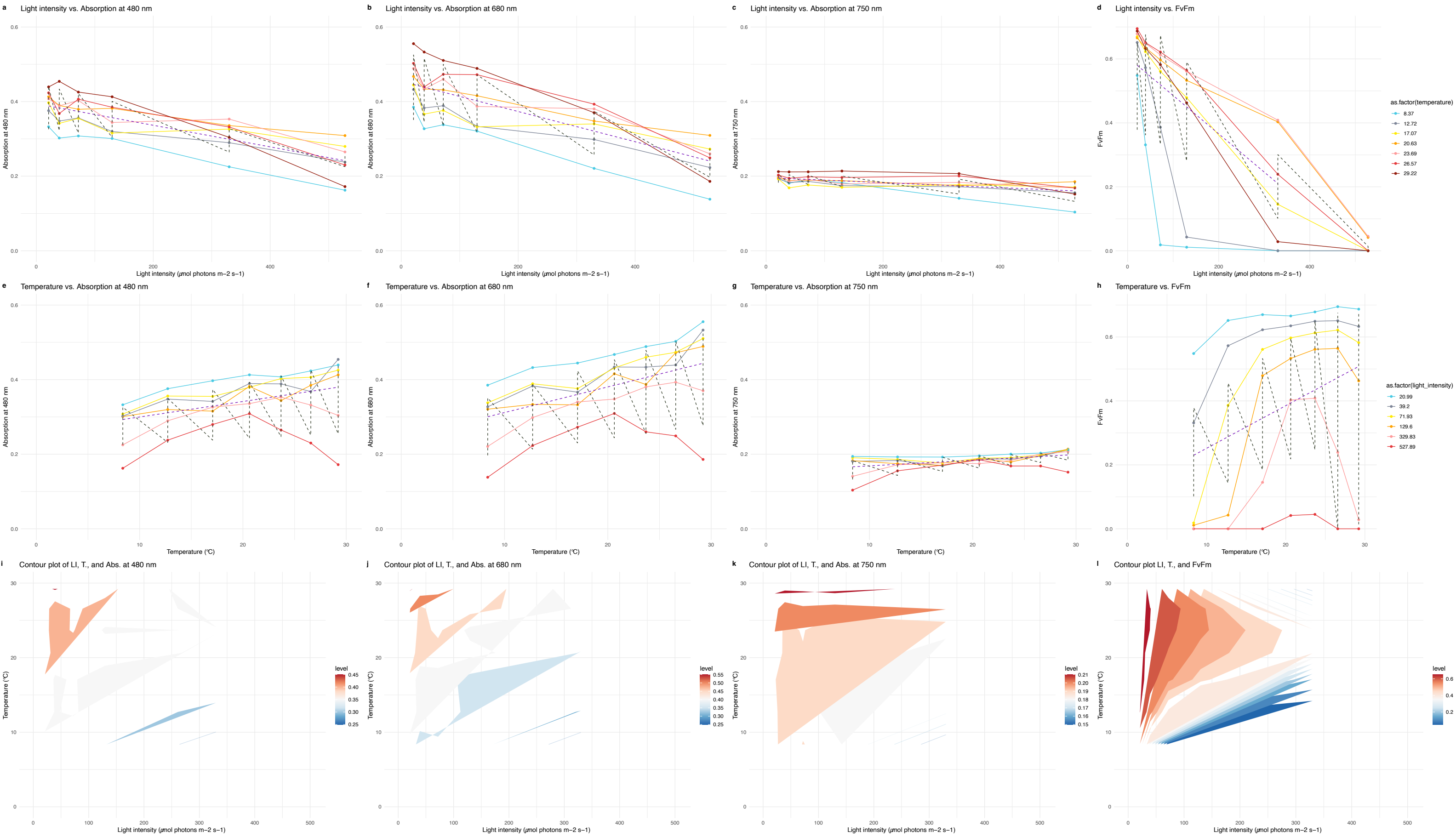

Supplementary Figure S1B: Regression analysis. Analysis of the independent parameters light (a-d) and temperature (e-h) on the dependent physiological/algal performance values absorption and Fv/Fm. Contour plots (i-j) highlight the combined effects of the independent parameters on algal performance indices.

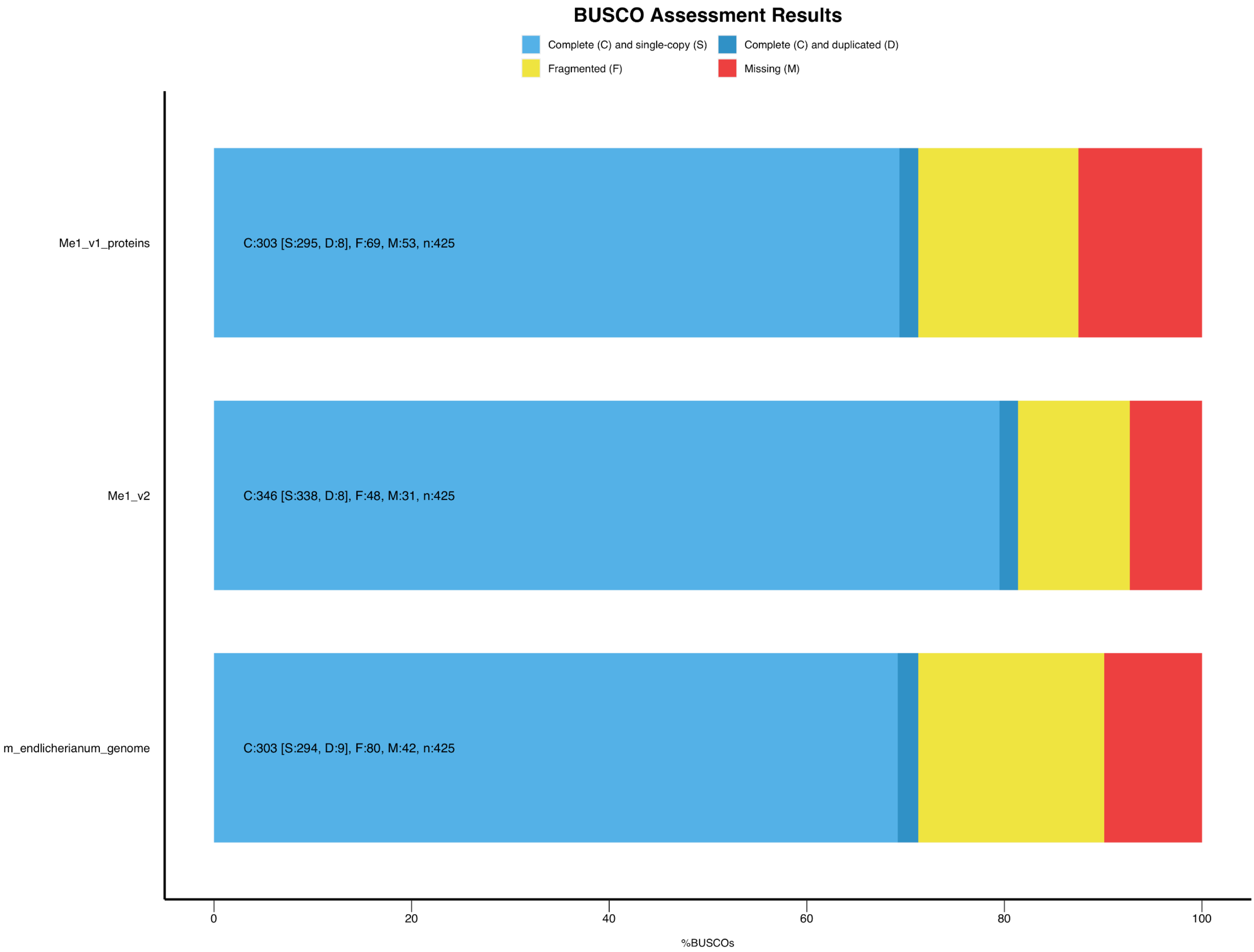

Supplementary Figure S2: Comparison of BUSCO scores. Comparisosns were carried out between genome, protein sequences V1, and protein sequences V2. The BUSCO score of annotation V2 resulted in less missing (red), less fragmented (yellow) and more complete (blue) genes.

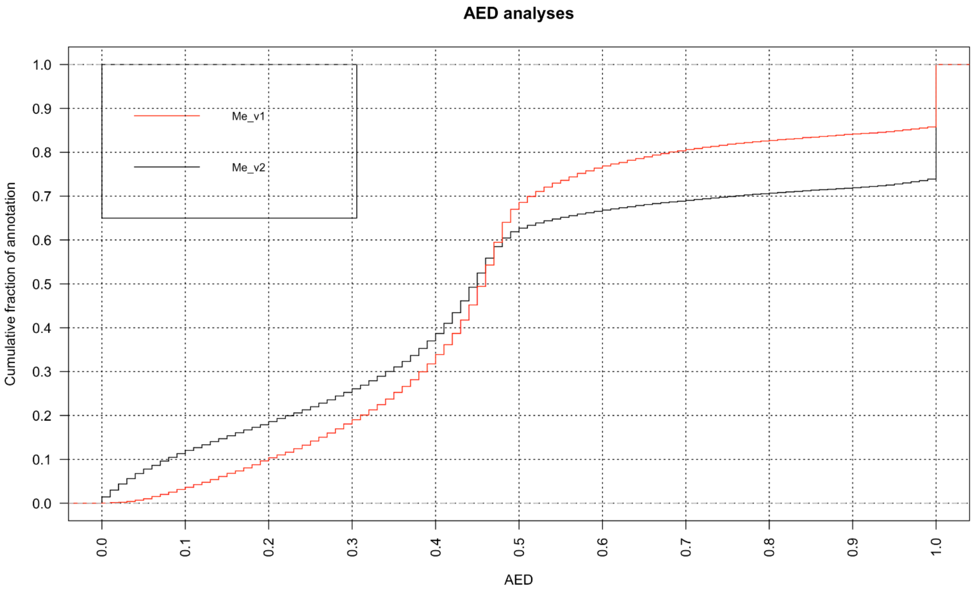

Supplementary Figure S3A: Quality assessment of the annotations. Cumulative fraction of annotation versus annotation edit distance (AED) plot for annotations V1 and V2. The red line marks V1, the black line marks V2.

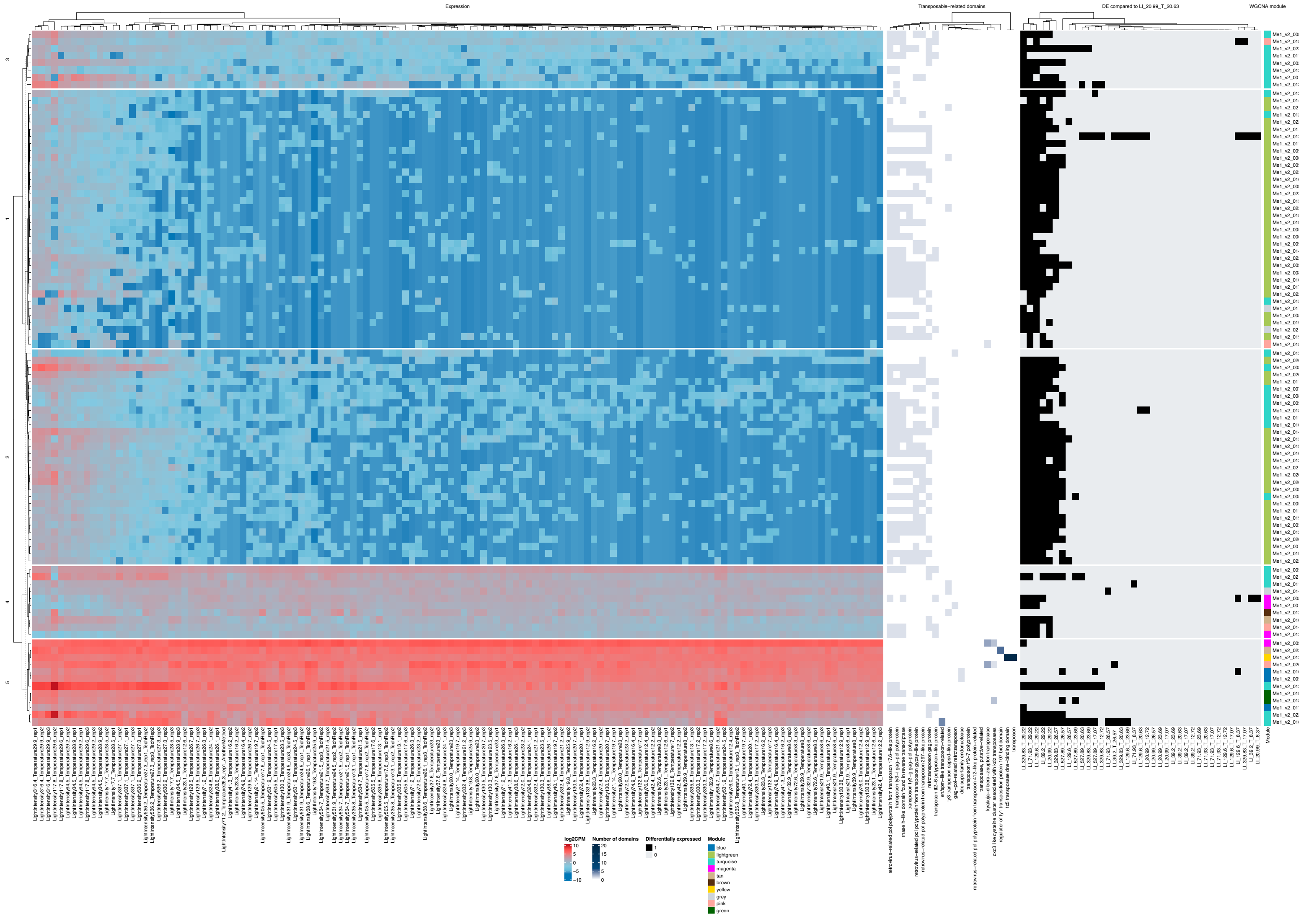

Supplementary Figure S3B: Expression of the 96 genes related to transposon biology. On the left, a heatmap shows the log2-transformed count per milion of all 96 genes that (i) are predicted to code for proteins with domains salient to transposon biology and (ii) passed the expression threshold. In the middle, the type and number of transposon-related domains predicted for these proteins are shown. On the right, a matrix indicates whether these genes were significantly differentially expressed when comparingsamples from the conditions named on the x-axis versus the algal samples exposed to 20°C and 20 µmol photons m^{-2} s^{-1}. The assignment of each gene's co-expression module is shown beside the gene names.

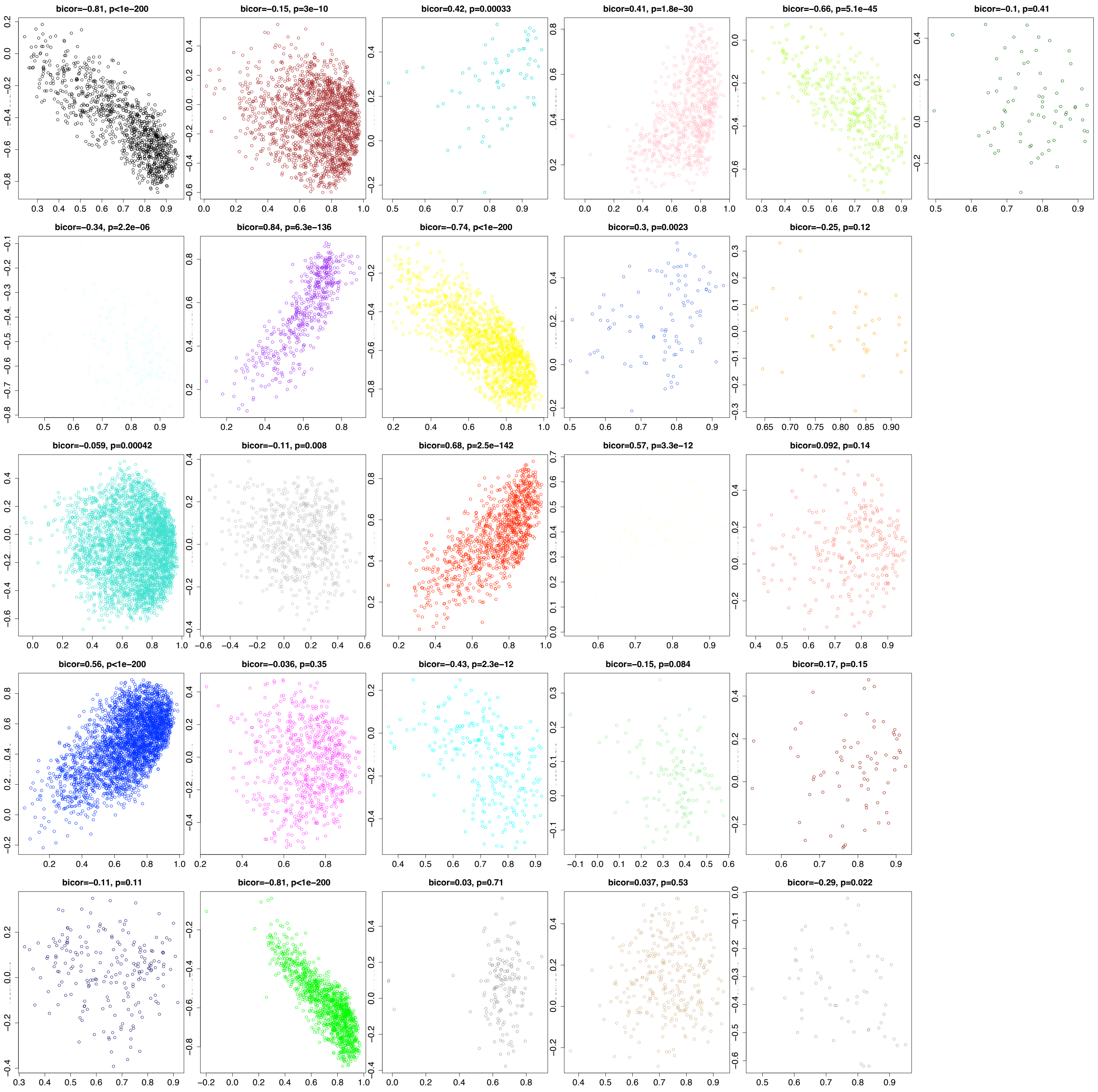

Supplementary Figure S4: Module membership versus Gene Significance for genes in different modules with respect to Fv/Fm. Colors correspond to the module name. X-axis shows "Module Membership in the module" and Y-axis shows "Gene significance for trait (Fv/Fm)". Bicor (biweight midcorrelation) was used as a robust measure for correlation in co-expression analysis; non-adjusted Student asymptotic p-values for multiple correlations were calculated. Exact p-values are shown except if p-value < 1e-200, then it was replaced by <1e-200. Statistics were one-sided.

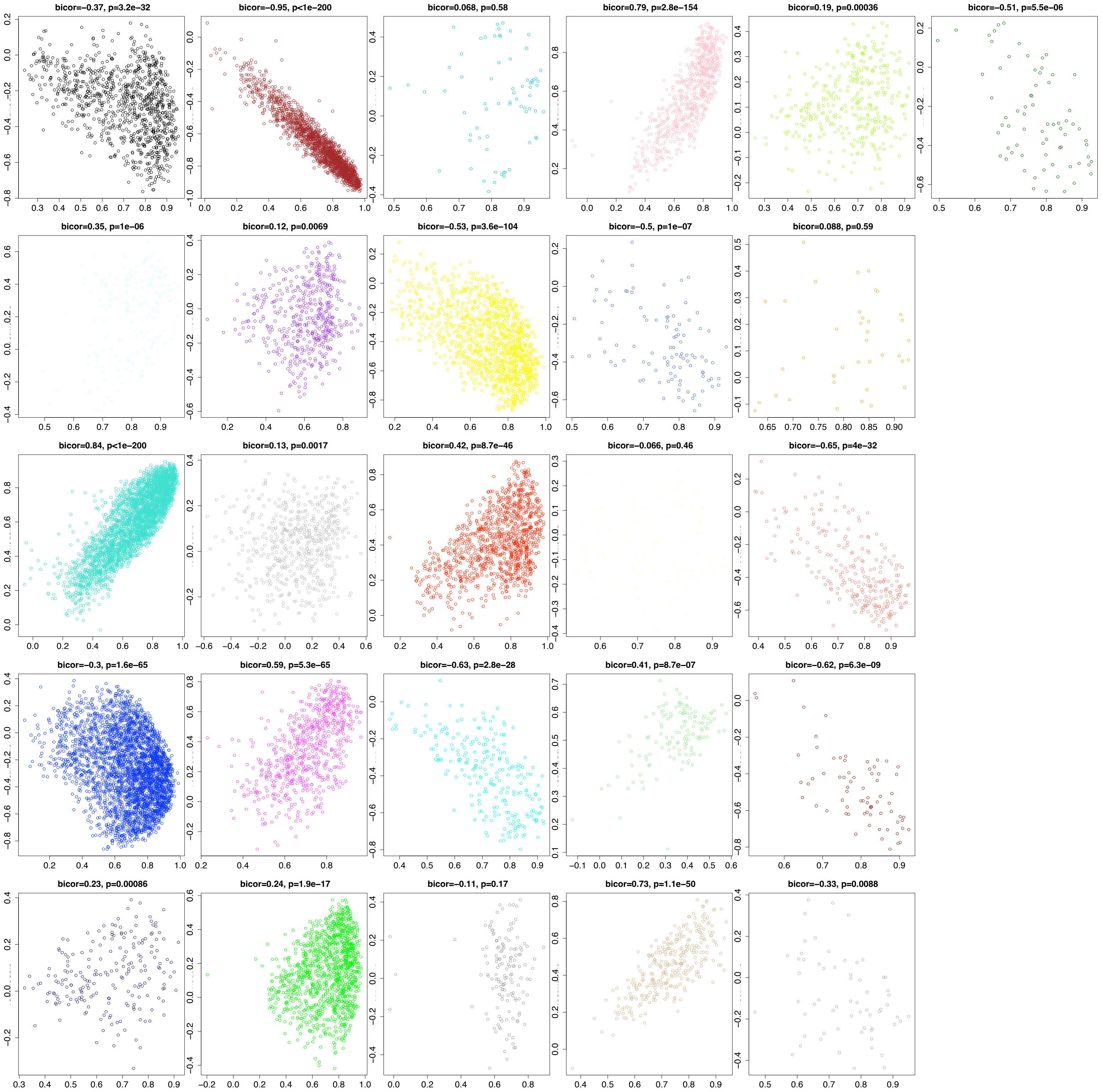

Supplementary Figure S5: Module membership versus Gene Significance for genes in different modules with respect to Temperature. Colors correspond to the module name. X-axis shows "Module Membership in the module" and Y-axis shows "Gene significance for trait (Temperature)". Bicor (biweight midcorrelation) was used as a robust measure for correlation in co-expression analysis; non-adjusted Student asymptotic p-values for multiple correlations were calculated. Exact p-values are shown except if p-value < 1e-200, then it was replaced by <1e-200. Statistics were one-sided.

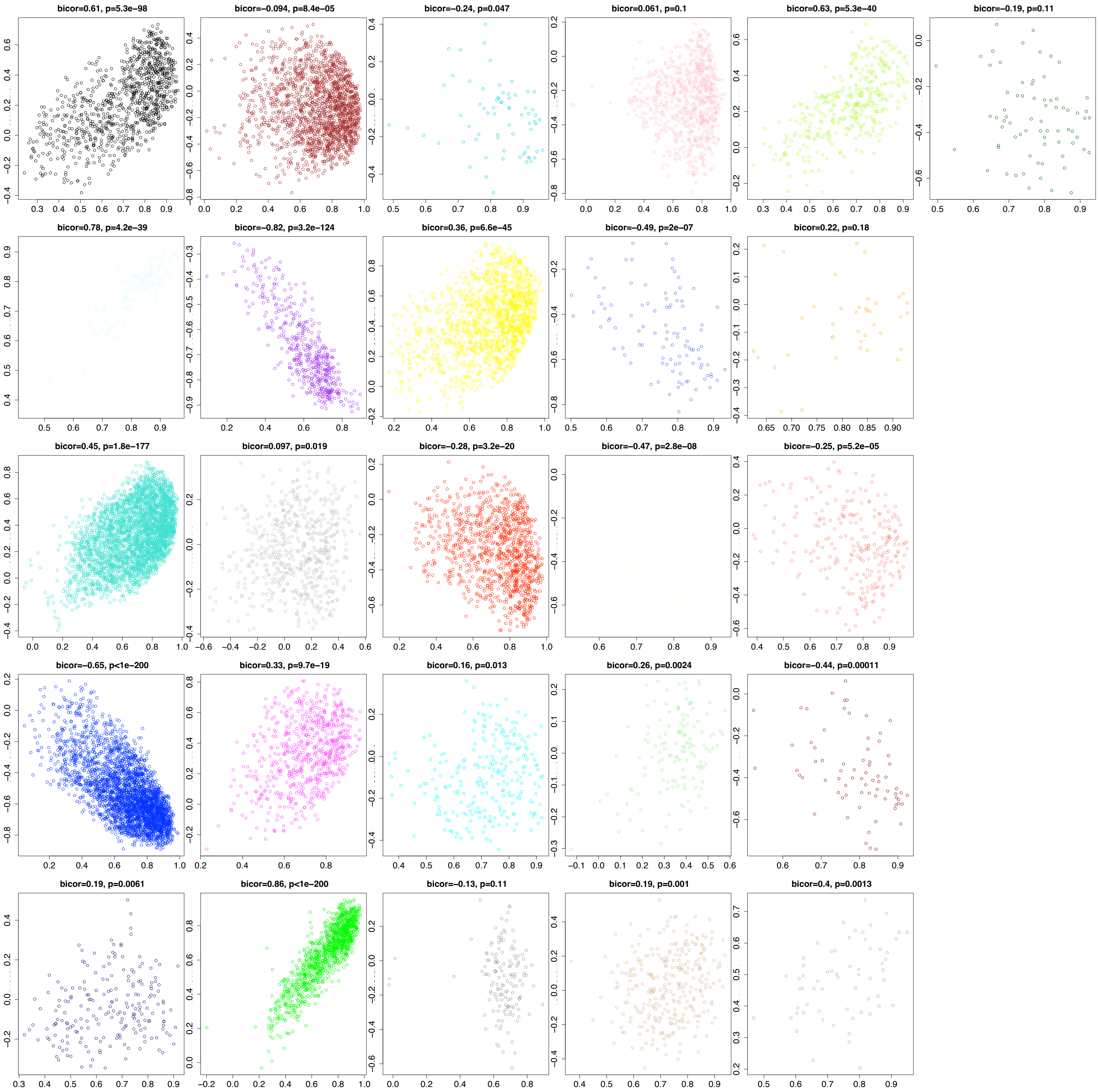

Supplementary Figure S6: Module membership versus Gene Significance for genes in different modules with respect to light intensity. Colors correspond to the module name. X-axis shows "Module Membership in the module" and Y-axis shows "Gene significance for trait (light intensity)". Bicor (biweight midcorrelation) was used as a robust measure for correlation in co-expression analysis; non-adjusted Student asymptotic p-values for multiple correlations were calculated. Exact p-values are shown except if p-value < 1e-200, then it was replaced by <1e-200. Statistics were one-sided.

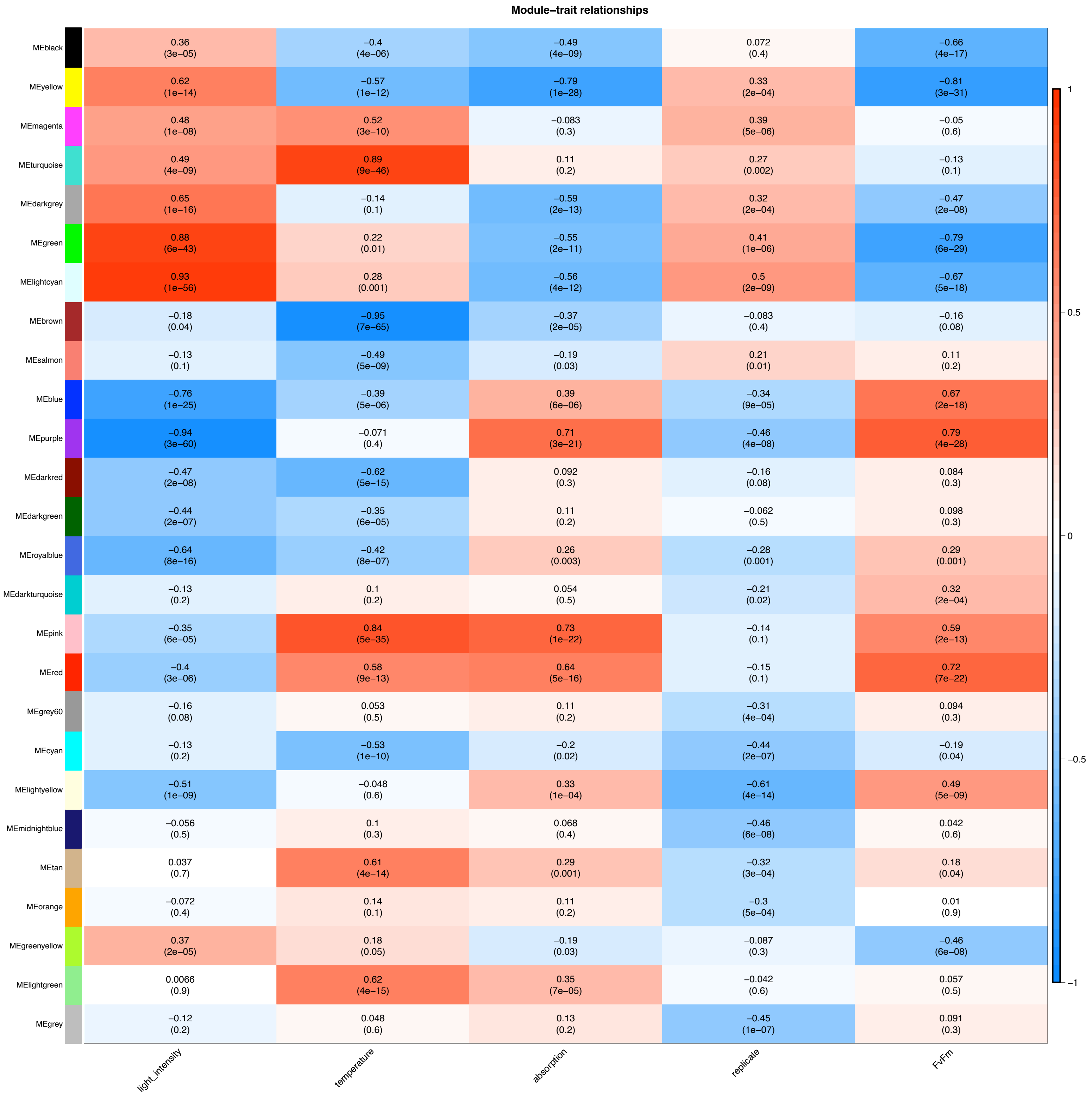

Supplementary Figure S7: Module–trait correlation based on eigengenes. Heatmap of the correlation between module eigengene expression behaviour and the parameters light intensity, temperature, absorption, replicate, and Fv/Fm as well as Student test p-value. Corresponding to the main Figure 4b. Statistics were one-sided.

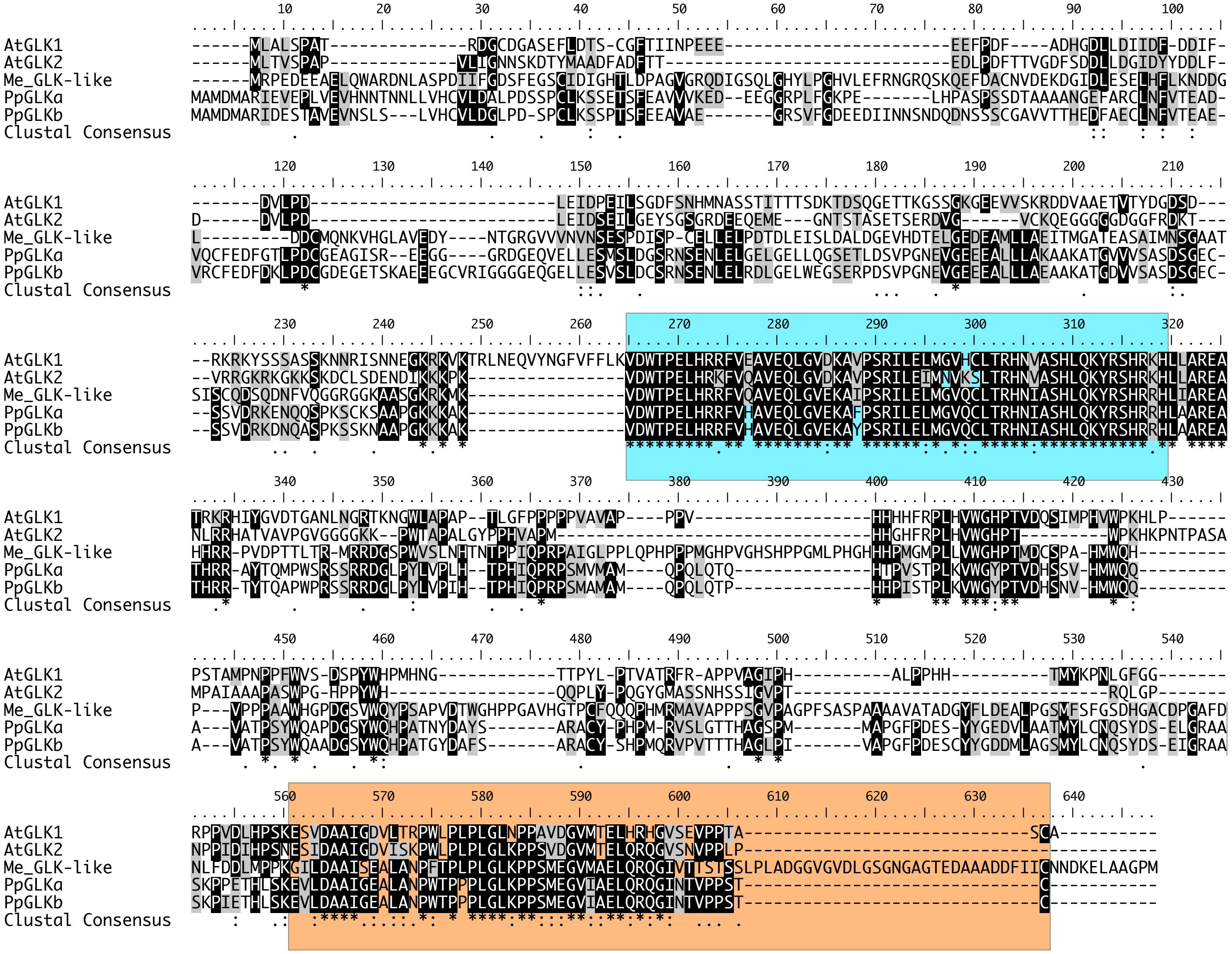

Supplementary Figure S8: Alignment Golden2-like homologs and domains. GLK homologs of Arabidopsis (At), *Mesotaenium endlicherianum* (Me), *Physcomitrium patens* (Pp) were aligned and a consensus was computed. Teal labels the DBD region, orange the GCT box.

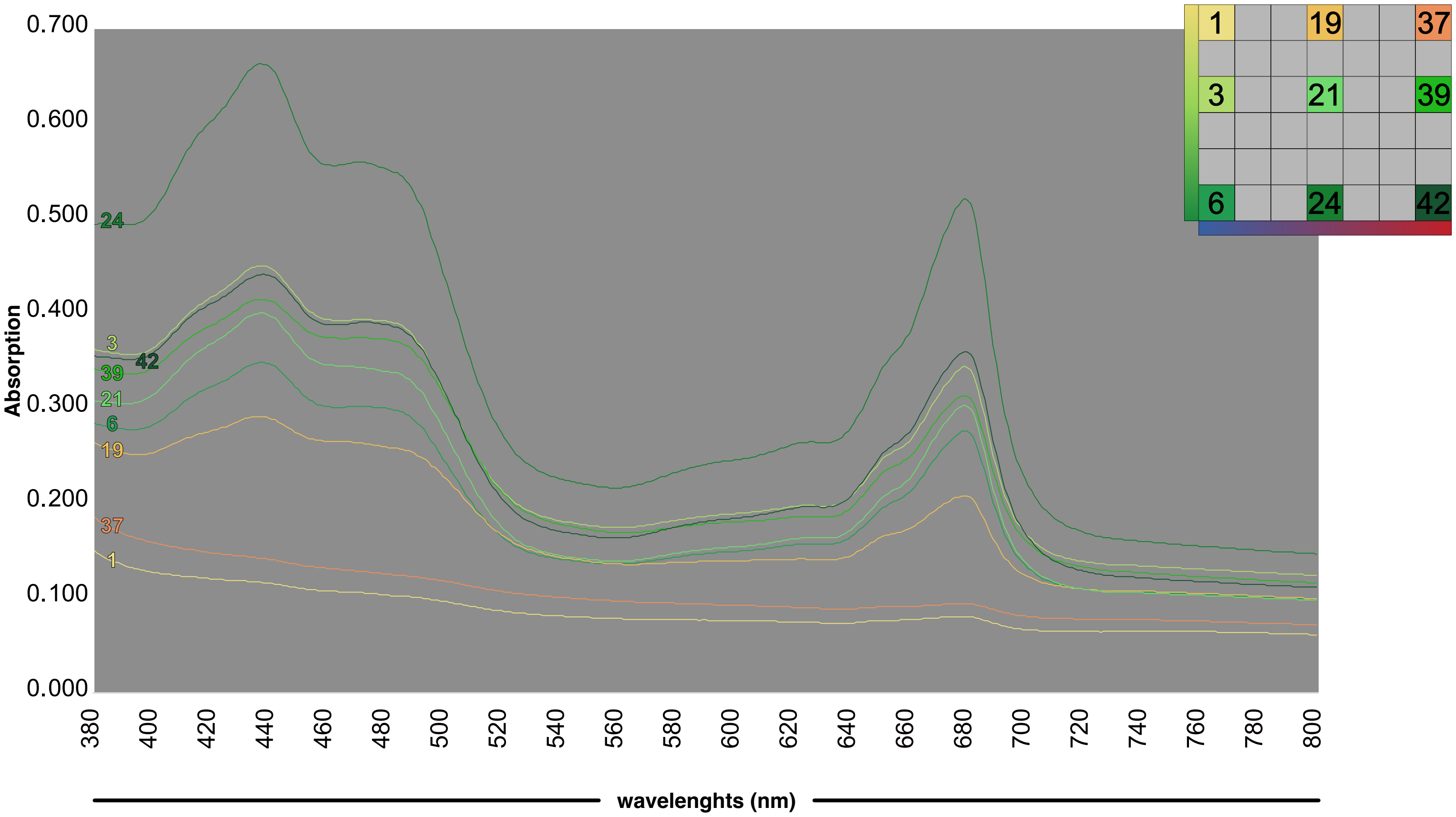

Supplementary Figure S9: Full Absorption Spectra. Shown are measured absorption spectra measured from λ 380 nm to λ 800 nm. Averaged values of three biological triplicates are shown (see individual values in Suppl. Table 10). Measured samples were taken from the most extreme environmental conditions (samples 1, 6, 37, 42) as well as along the whole temperature gradient at 130 µmol photons m^{-2} s^{-1} (samples 3, 21, 39) and along the whole irradiance gradient at 21°C (samples 24, 21, 19).

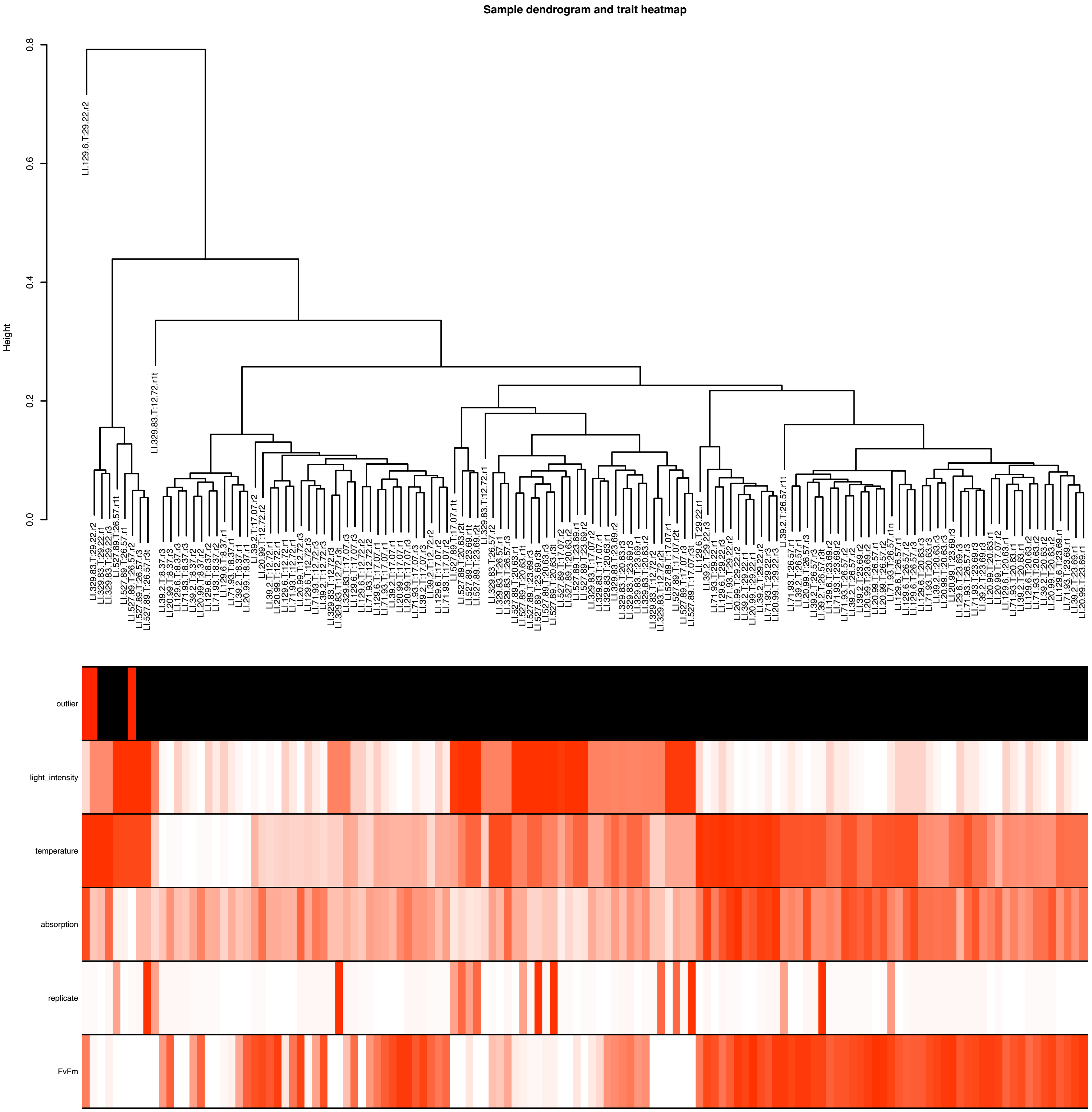

Supplementary Figure S10: Samples analysed in WGCNA. A sample dendrogram was computed (top) and projected on top of a trait heatmap to identify outliers for WGCNA.

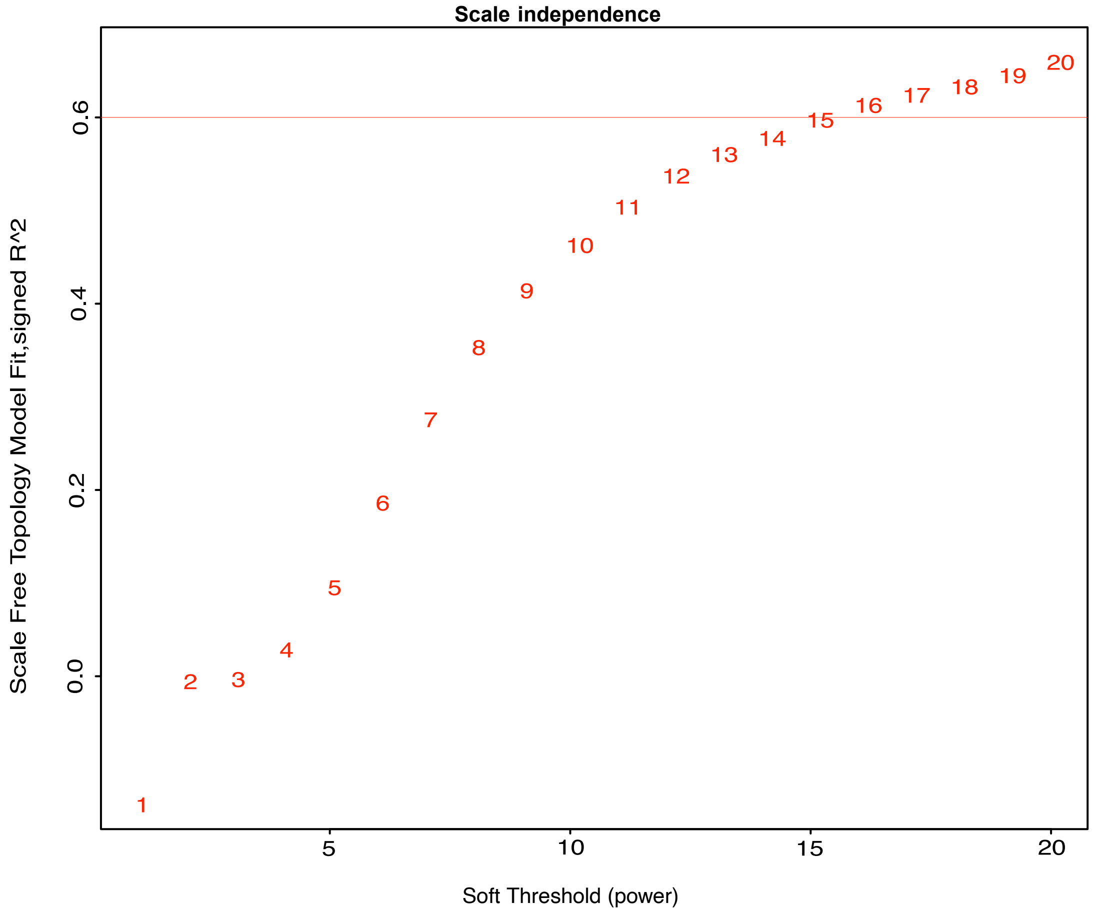

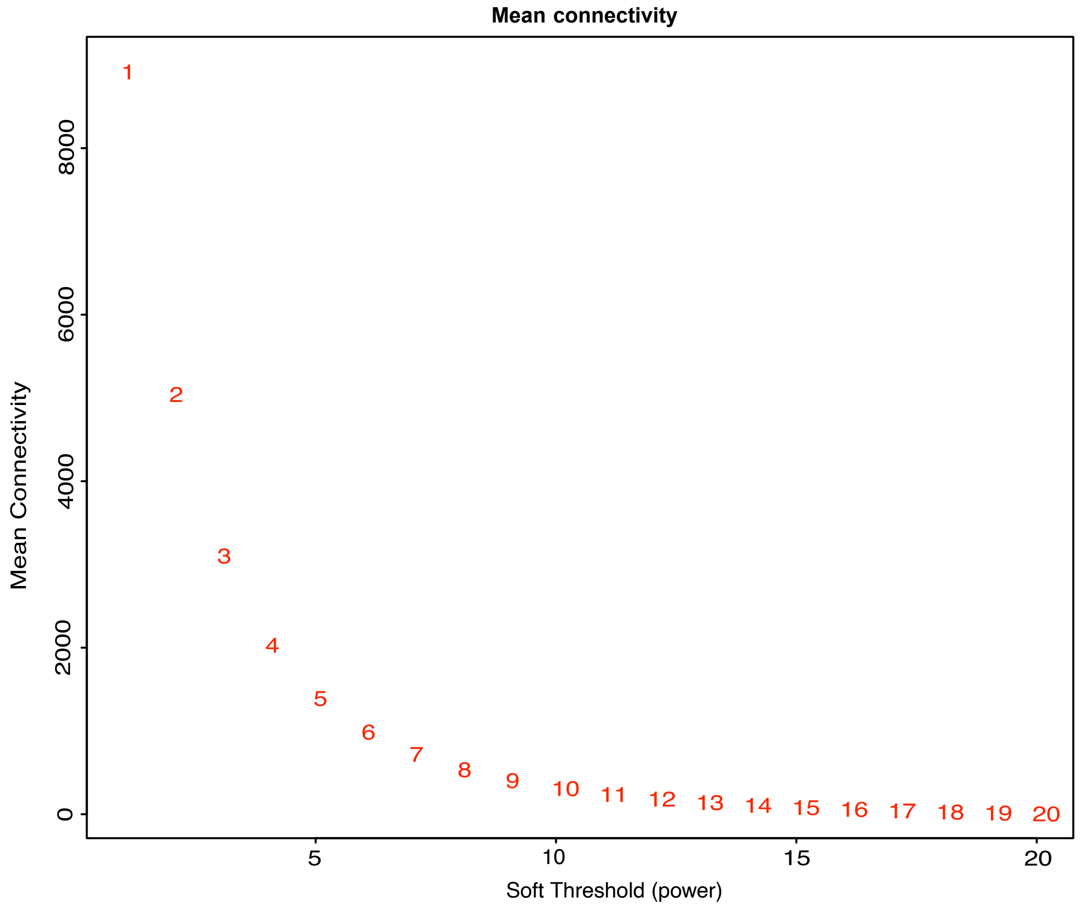

Supplementary Figure S11: Picking a soft threshold for WGCNA based on scale independence and Mean connectivity. Top: Scale-free topology index versus the power values 1 to 20; bottom: the mean connectivity versus the power values 1 to 20.

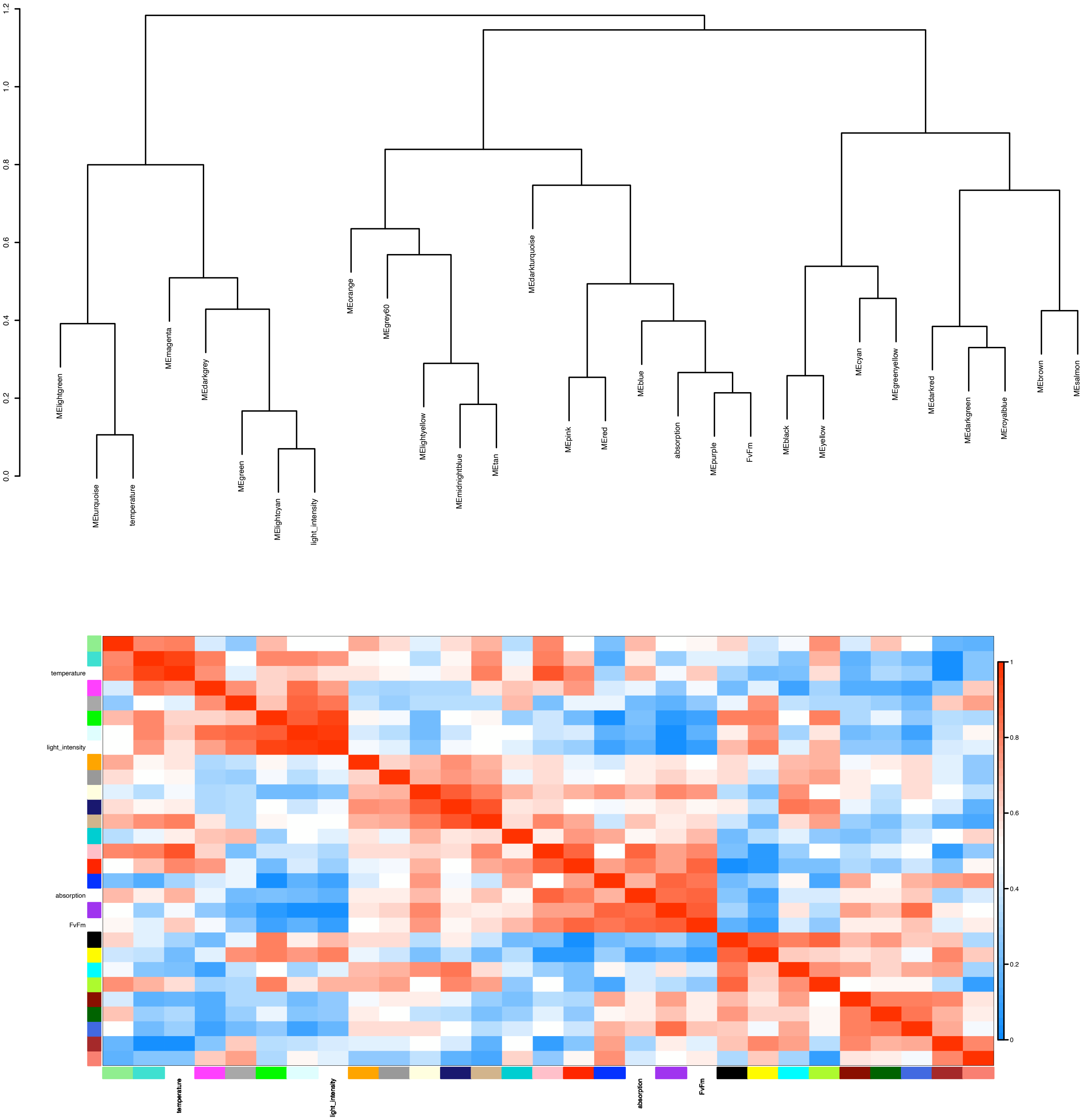

Supplementary Figure S12: Clustering of different modules and traits. Correlations between the modules (defined based on eigengenes) and the traits plotted as a heatmap from maximum correlation (1, red) to minimum (0, blue). The dendogram on the top shows the relationship between all 26 modules and the traits. The figure illustrates that a proper merging threshold was chosen. If the merging threshold is too small, many more modules would emerge, if overmerging were performed, unrelated genes would end up in one module.

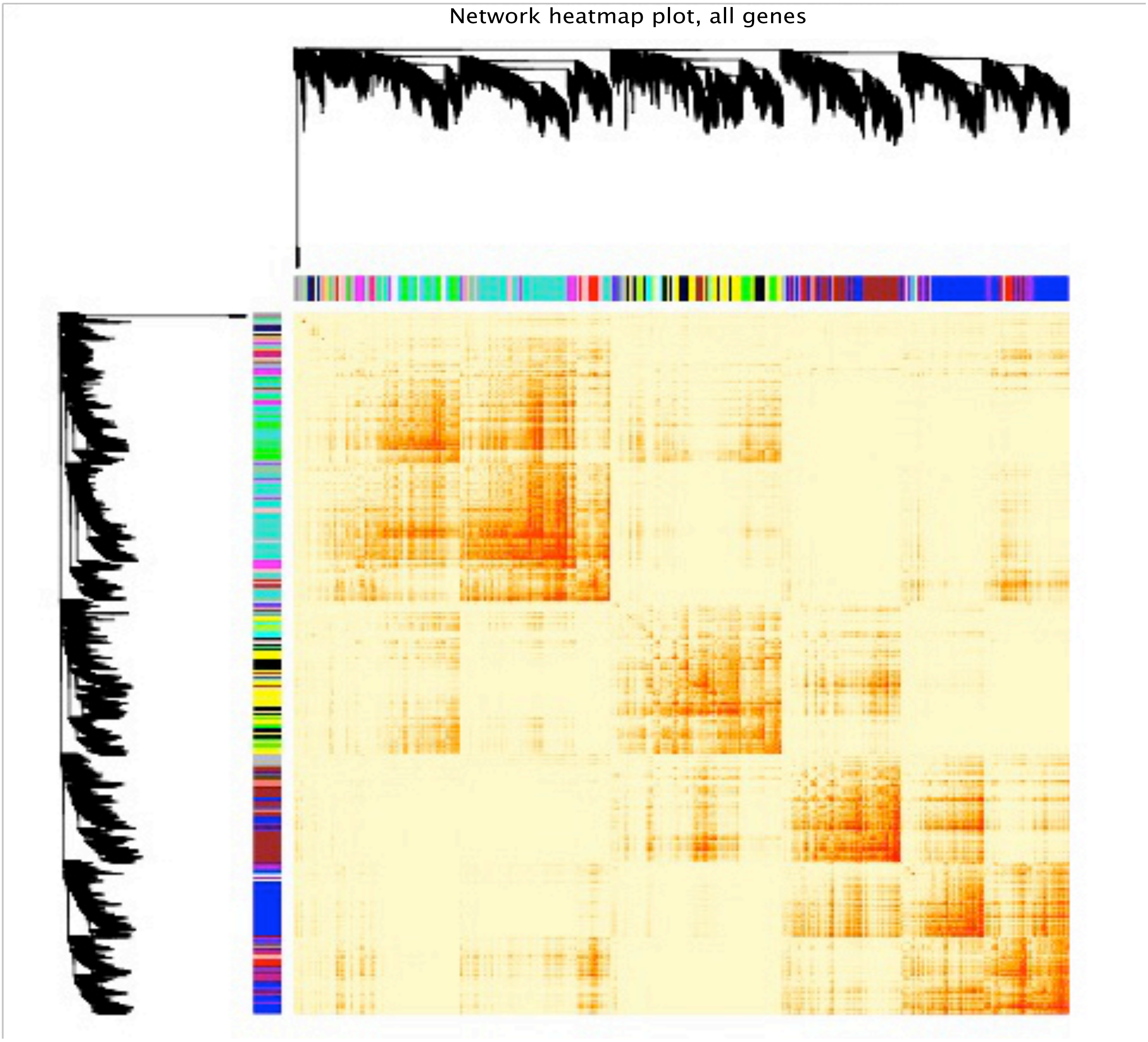

Supplementary Figure S13: The graphical representation of the topological overlap matrix. A plot of the connections of all genes versus all genes of Mesotaenium in the network. Dark orange represents

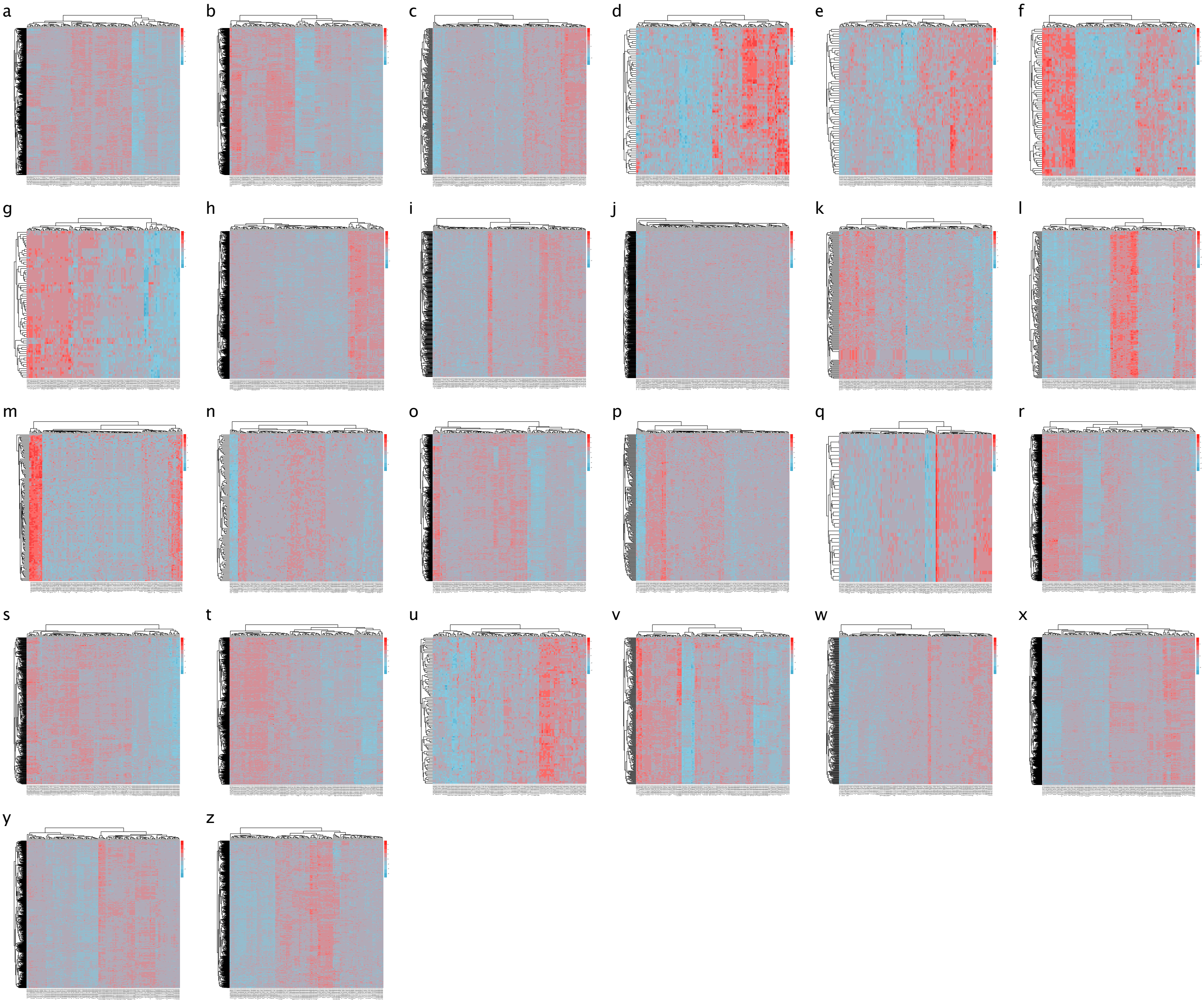

Supplementary Figure S14A: Heatmap of gene expression Z-score values for each module. (a) blue **(b)** brown **(c)** cyan **(d)** darkgreen **(e)** darkgray **(f)** darkred **(g)** darkturquoise **(h)** green **(i)** green yellow **(j)** grey **(k)** grey60 **(l)** light cyan **(m)** light green **(n)** light yellow **(o)** magenta **(p)** midnight blue **(q)** orange **(r)** pink **(s)** purple **(t)** red **(u)** royal blue **(v)** salmon **(w)** tan **(x)** turquoise **(y)** yellow **(z)** black

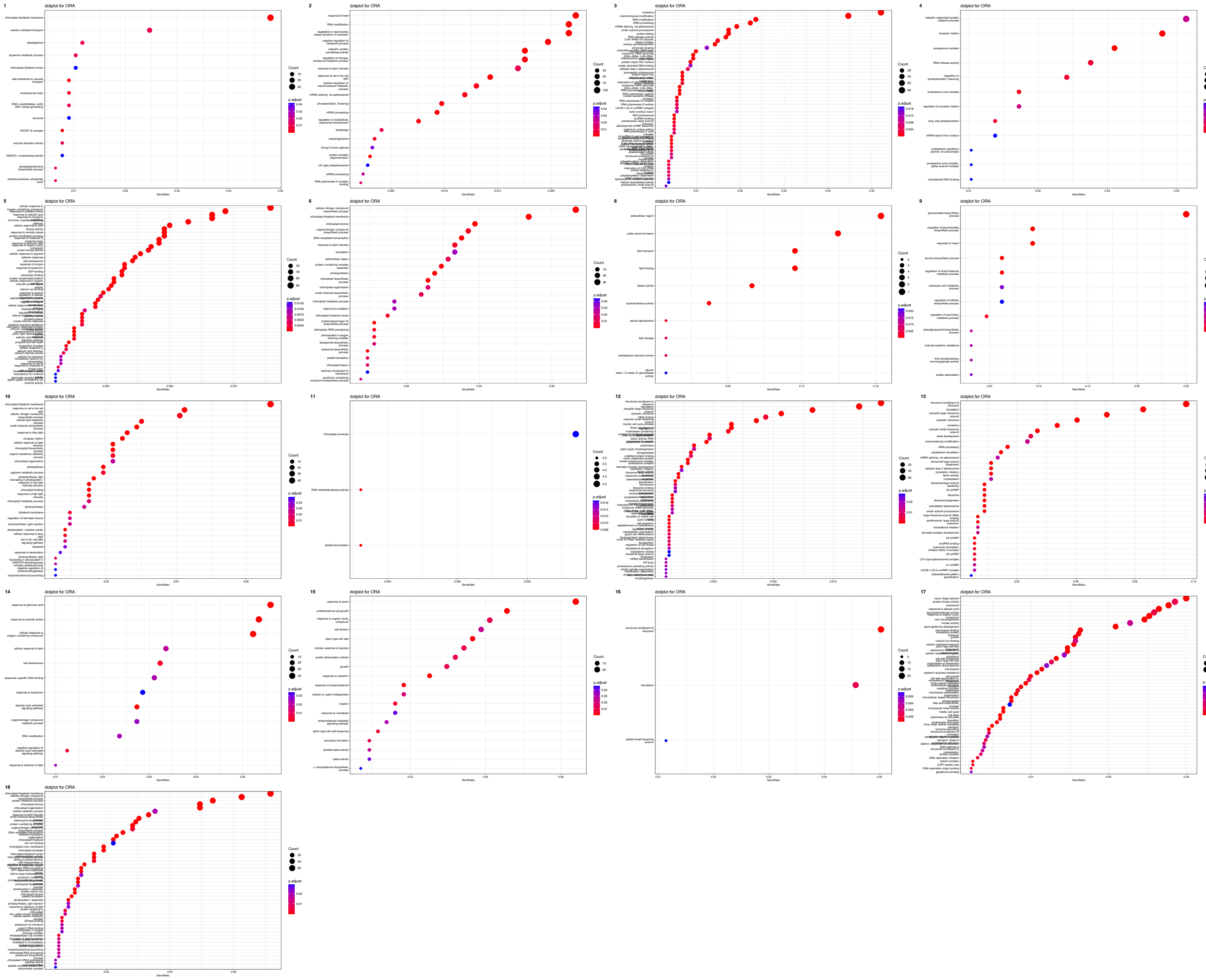

Supplementary Figure S14B: GO enrichment analysis for *A. thaliana*. Numbers in the top left corner correspond to the module numbers as in Figure 5F of the manuscript. We only visualized enriched GO terms with adj. p-value < 0.05 and if a module has no enriched terms, obviously, we could not visualize GO terms for that module. The enricher function from clusterProfiler package was used to perform enrichment analysis. A hypergeometric test comparing the set of "significant" genes against the "universe" (or background) genes was performed. Statistics is based on a one-sided test and the Benjamini-Hochberg method was used for adjustment for multiple testing. The adjusted p-values are plotted as color gradient. All p-values, adjusted p-values and q-values are shown in Supplementary Table S15.

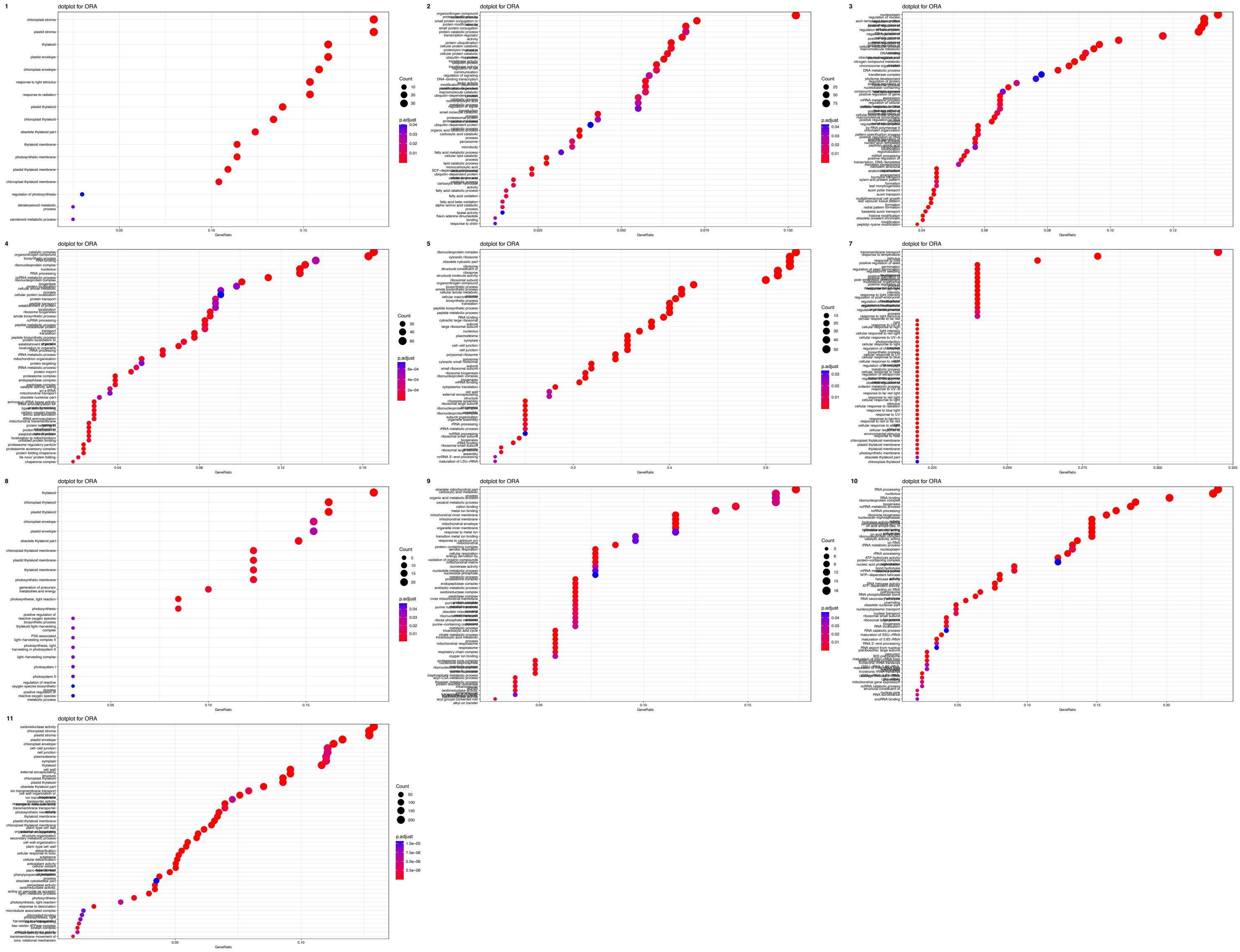

Supplementary Figure S14C: GO enrichment analysis for *M. polymorpha*. Numbers in the top left corner correspond to the module numbers as in Figure 5F of the manuscript. We only visualized enriched GO terms with adj. p-value < 0.05 and if a module has no enriched terms, obviously, we could not visualize GO terms for that module. The enricher function from clusterProfiler package was used to perform enrichment analysis. A hypergeometric test comparing the set of "significant" genes against the "universe" (or background) genes was performed. Statistics is based on a one-sided test and the Benjamini-Hochberg method was used for adjustment for multiple testing. The adjusted p-values are plotted as color gradient. All p-values, adjusted p-values and q-values are shown in Supplementary Table S15.

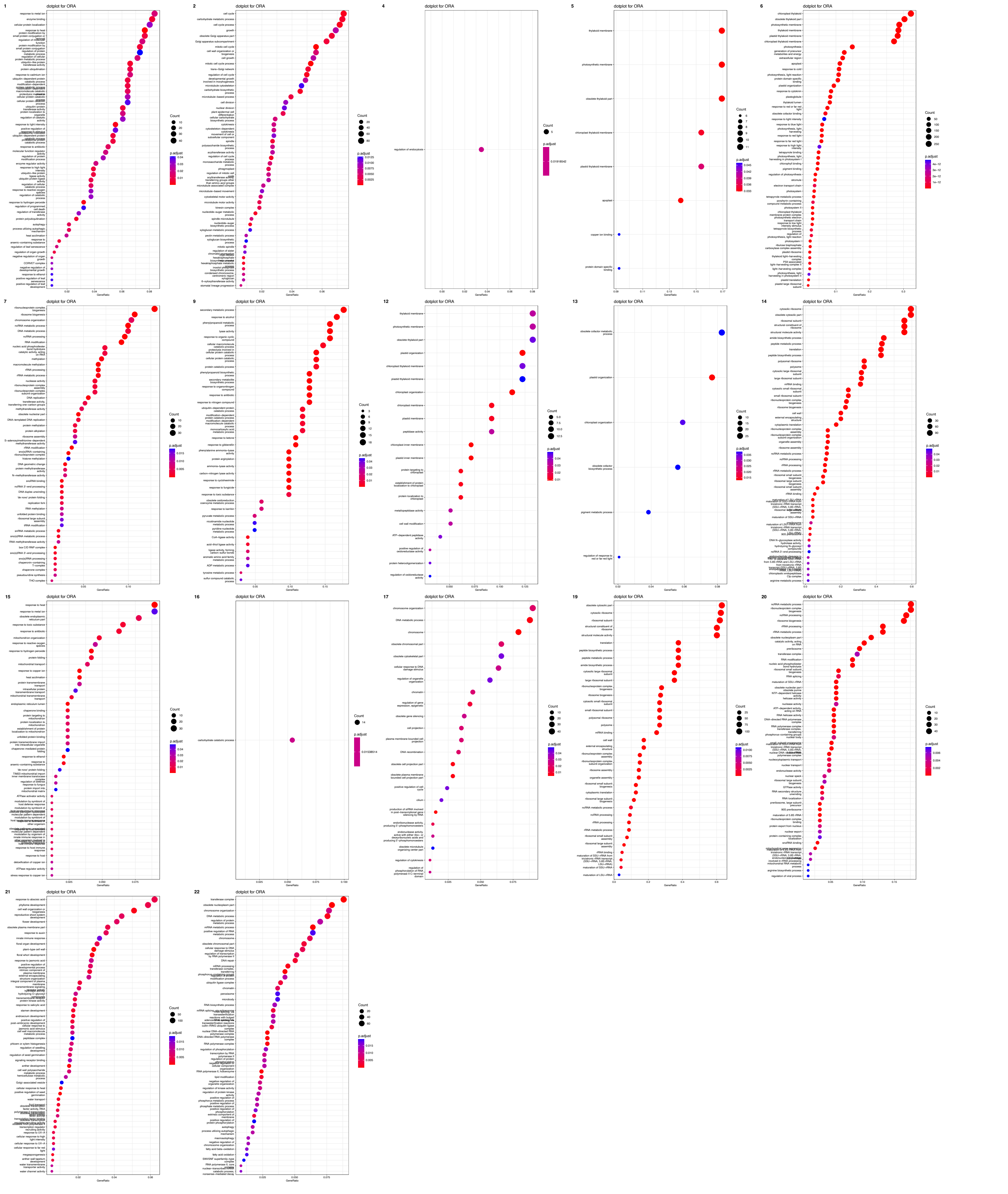

Supplementary Figure S14D: GO enrichment analysis for *P. patens*. Numbers in the top left corner correspond to the module numbers as in Figure 5F of the manuscript. We only visualized enriched GO terms with adj. p-value < 0.05 and if a module has no enriched terms, obviously, we could not visualize GO terms for that module. The enricher function from clusterProfiler package was used to perform enrichment analysis. A hypergeometric test comparing the set of "significant" genes against the "universe" (or background) genes was performed. Statistics is based on a one-sided test and the Benjamini-Hochberg method was used for adjustment for multiple testing. The adjusted p-values are plotted as color gradient. All p-values, adjusted p-values and q-values are shown in Supplementary Table S15.

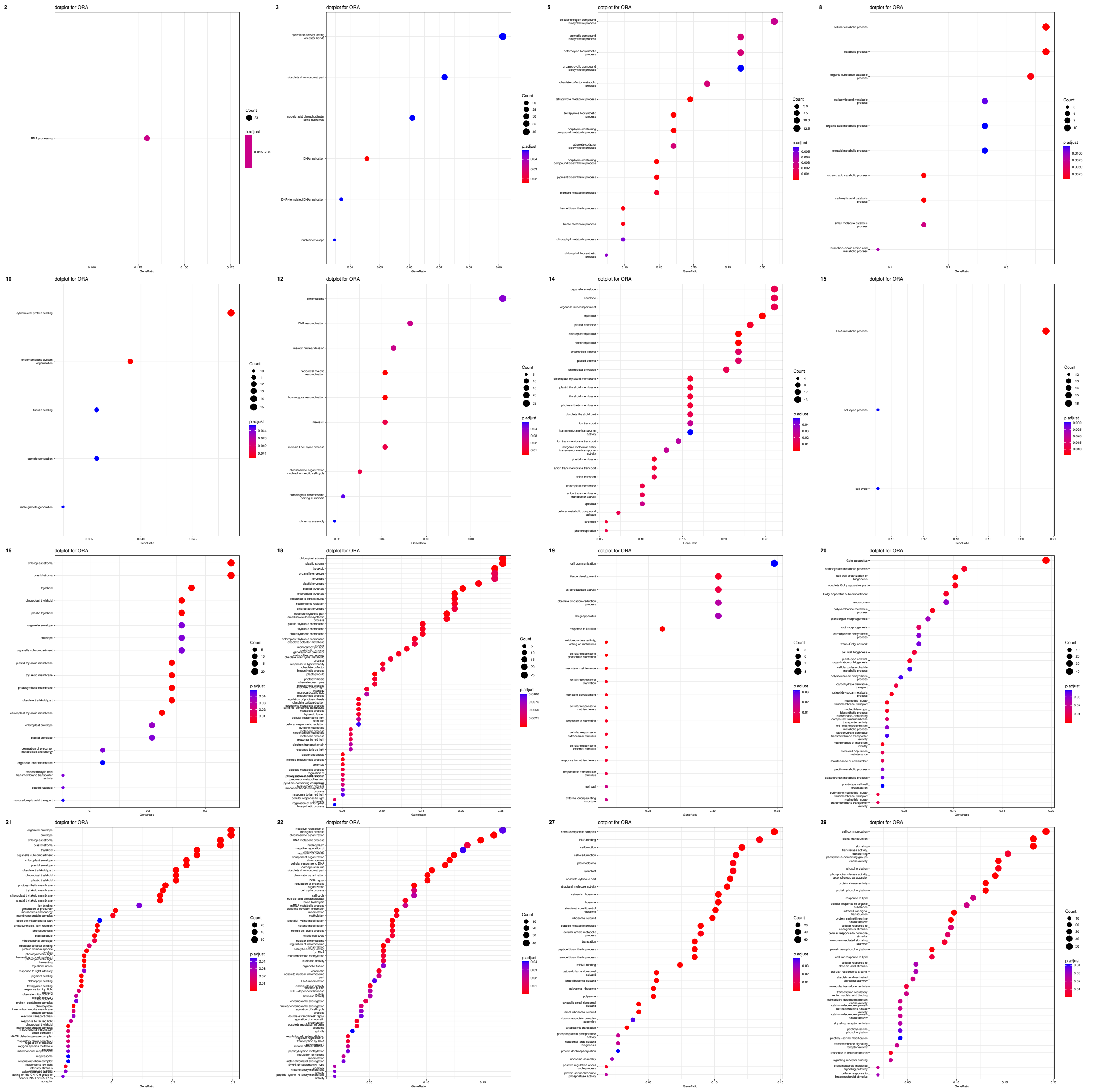

Supplementary Figure S14E: GO enrichment analysis for *Zygnema* SAG 698-1b. Numbers in the top left corner correspond to the module numbers as in Figure 5F of the manuscript. We only visualized enriched GO terms with adj. p-value < 0.05 and if a module has no enriched terms, obviously, we could not visualize GO terms for that module. The enricher function from clusterProfiler package was used to perform enrichment analysis. A hypergeometric test comparing the set of "significant" genes against the "universe" (or background) genes was performed. Statistics is based on a one-sided test and the Benjamini-Hochberg method was used for adjustment for multiple testing. The adjusted p-values are plotted as color gradient. All p-values, adjusted p-values and q-values are shown in Supplementary Table S15.

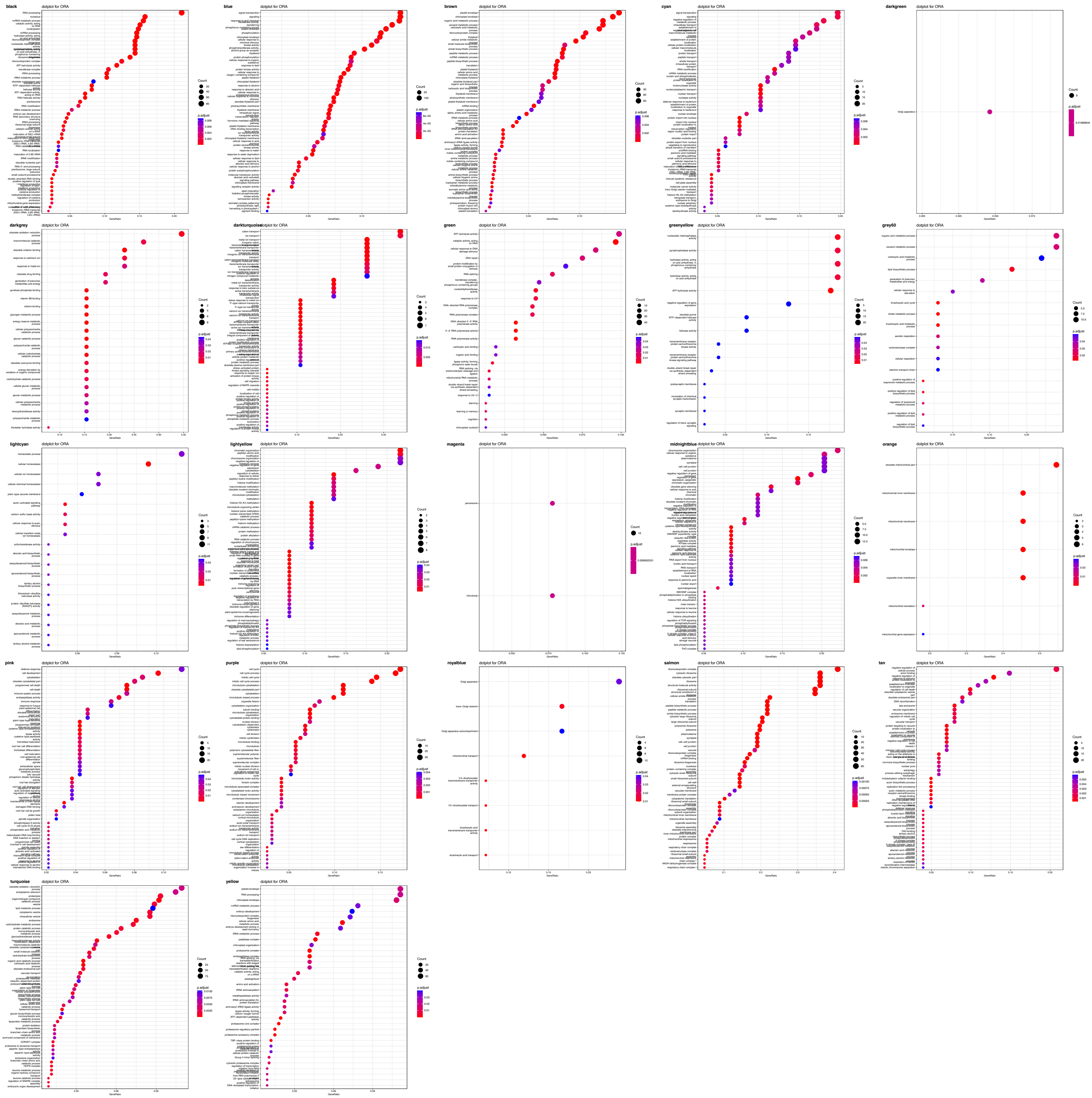

Supplementary Figure S14F: GO enrichment analysis for the 26 co-expression modules of *Mesotaenium*. Colors correspond to the module numbers as in Figure 5F of the manuscript. We only visualized enriched GO terms with adj. p-value < 0.05 and if a module has no enriched terms we did not visualize any GO terms for that module. The enricher function from clusterProfiler package was used to perform enrichment analysis. A hypergeometric test comparing the set of "significant" genes against the "universe" (or background) genes was performed. Statistics is based on a one-sided test and the Benjamini-Hochberg method was used for adjustment for multiple testing. The adjusted p-values are plotted as color gradient. All p-values, adjusted p-values and q-values are shown in Supplementary Table S6.

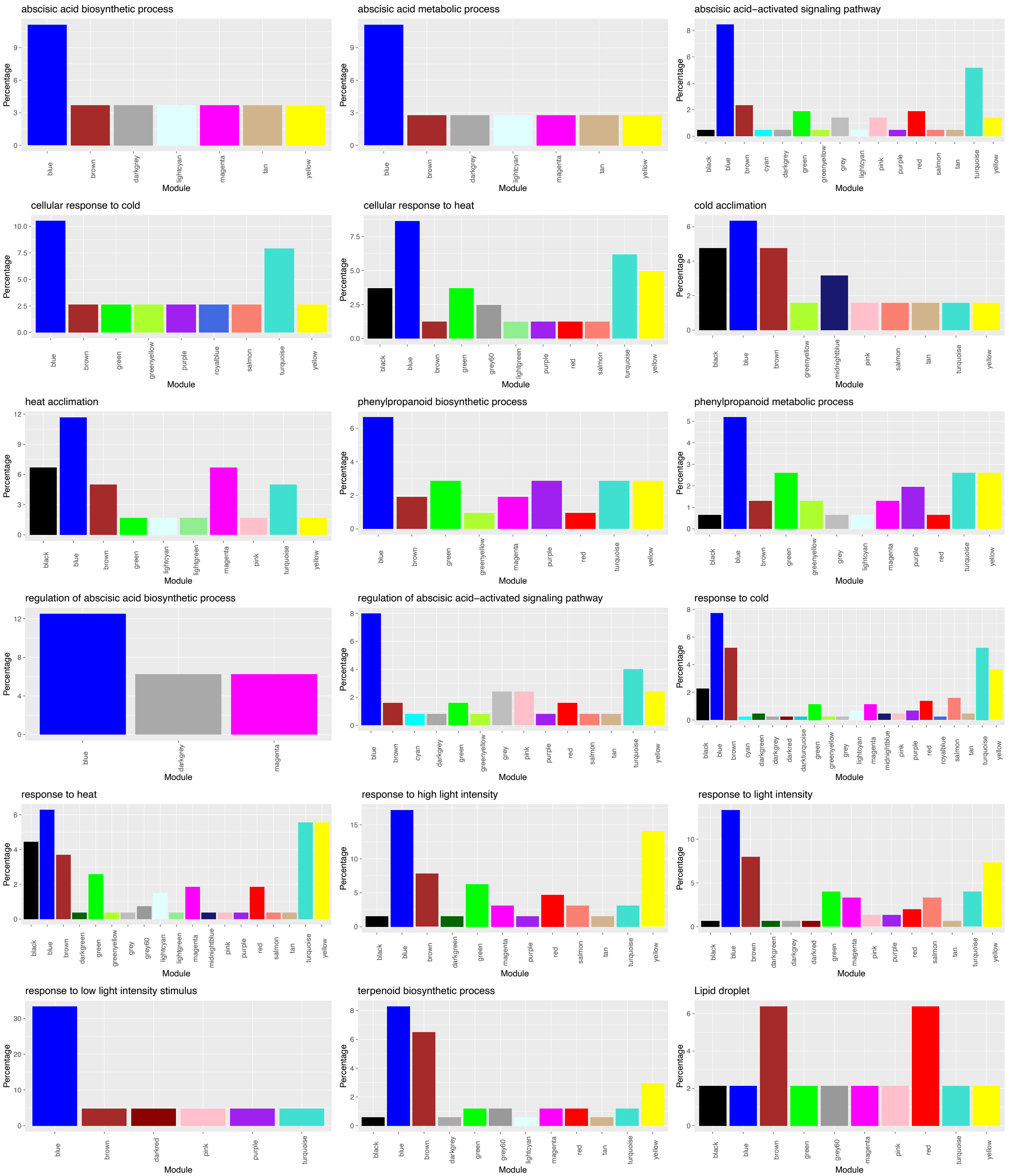

Supplementary Figure S15: Distribution of of *A. thaliana* homologs for stress response genes among WGCNA modules. Colors correspond to the 26 modules defined in main Figure 4a. Arabidopsis genes that were used as a query for homolog detection were based on keyword searches on TAIR and, in case of the lipid droplet-relevant genes, manually curated.

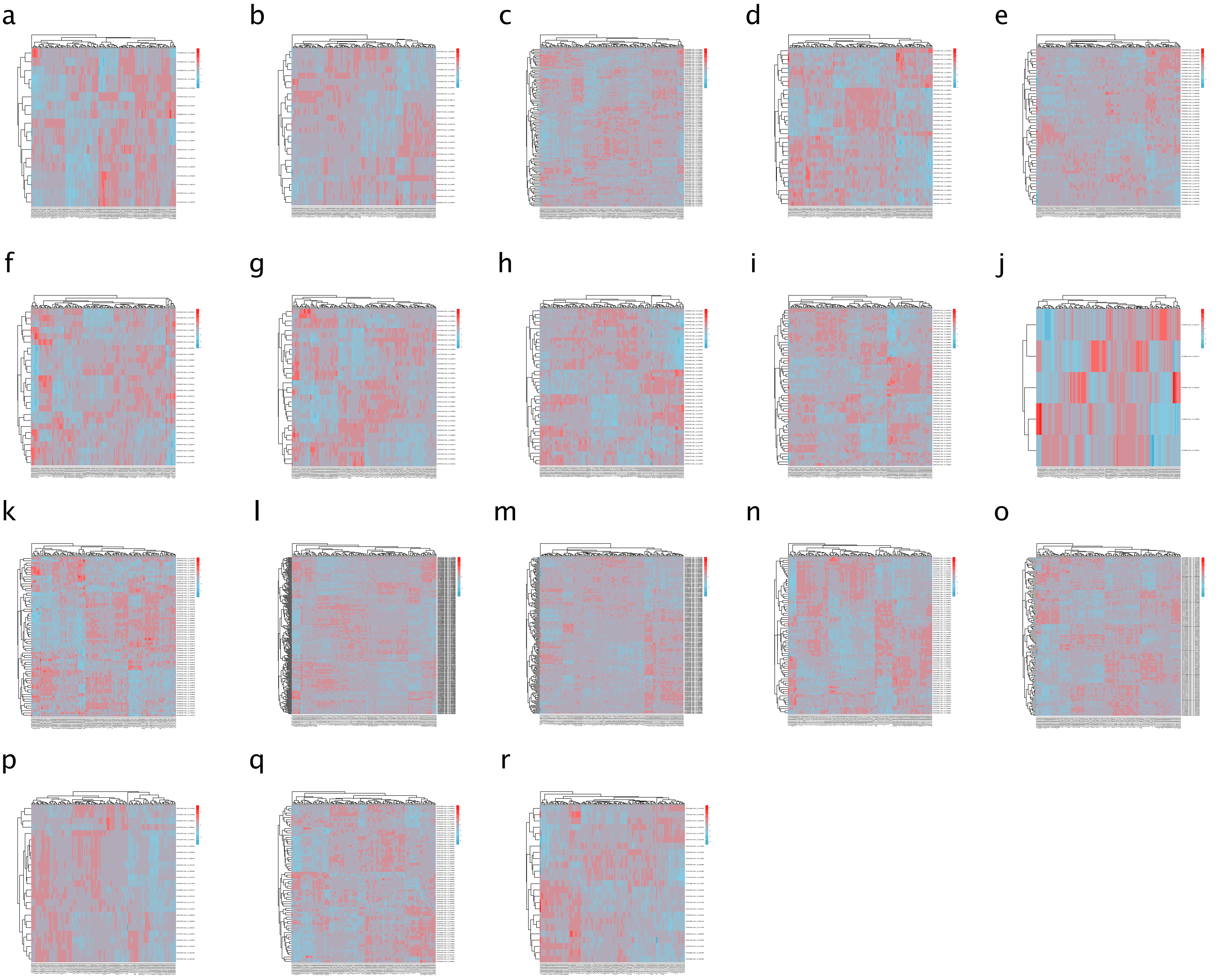

Supplementary Figure S16: Heatmap of best blast hit of *A. thaliana* stress response genes in *M. endlicherianum* across different growth conditions. **(a)** Abscisic acid biosynthetic process genes best blast hit gene expression **(b)** Abscisic acid metabolic process genes best blast hit gene expression **(c)** Abscisic acid−activated signaling pathway genes best blast hit gene expression **(d)** Cellular response to cold genes best blast hit gene expression **(e)** Cellular response to heat genes best blast hit gene expression **(f)** Cold acclimation genes best blast hit gene expression **(g)** Heat acclimation genes best blast hit gene expression **(h)** Phenylpropanoid biosynthetic process genes best blast hit gene expression **(i)** Phenylpropanoid metabolic process genes best blast hit gene expression **(j)** Regulation of abscisic acid biosynthetic process genes best blast hit gene expression **(k)** Regulation of abscisic acid−activated signaling pathway genes best blast hit gene expression **(l)** Response to cold genes best blast hit gene expression **(m)** Response to heat genes best blast hit gene expression **(n)** Response to high light intensity genes best blast hit gene expression **(o)** Response to light intensity genes best blast hit gene expression **(p)** Response to low light intensity stimulus genes best BLAST hit gene expression **(q)** Terpenoid biosynthetic process genes best blast hit gene expression **(r)** lipid droplet genes best blast hit gene expression

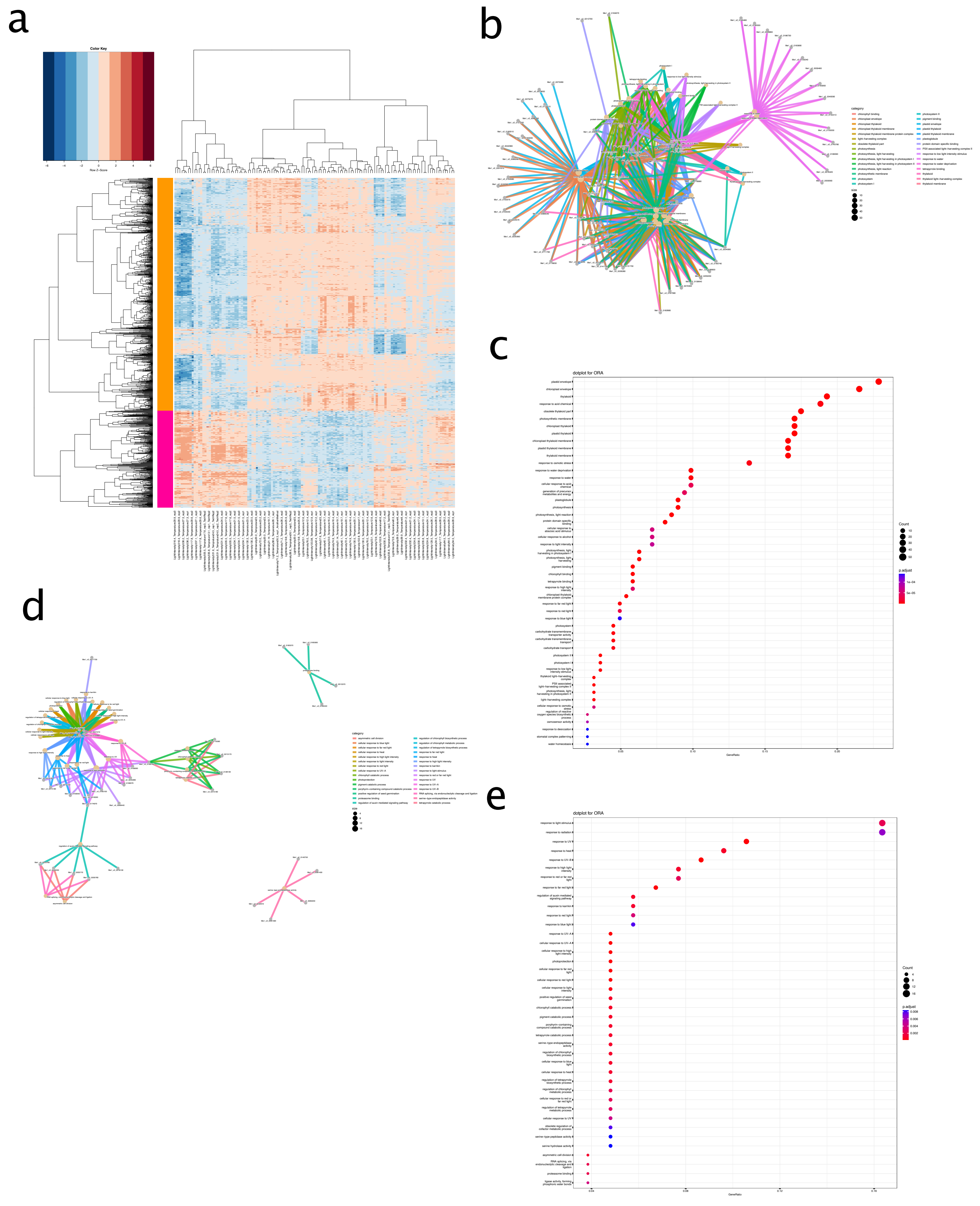

Supplementary Figure S17: DEGs and GO-enrichment of Fv /Fm vs control. (a) Heatmap of DEGs **(b)** cnetplot of orange cluster **(c)** dot plot of orange cluster **(d)** cnetplot of pink cluster **(e)** dot plot of pink cluster. For (c) and (e): The enricher function from clusterProfiler package was used to perform enrichment analysis. A hypergeometric test comparing the set of "significant" genes against the "universe" (or background) genes was performed. Statistics is based on a one-sided test and the Benjamini-Hochberg method was used for adjustment for multiple testing. The adjusted p-values are plotted as color gradient. All p-values, adjusted p-values and q-values are shown in Supplementary Table S14.

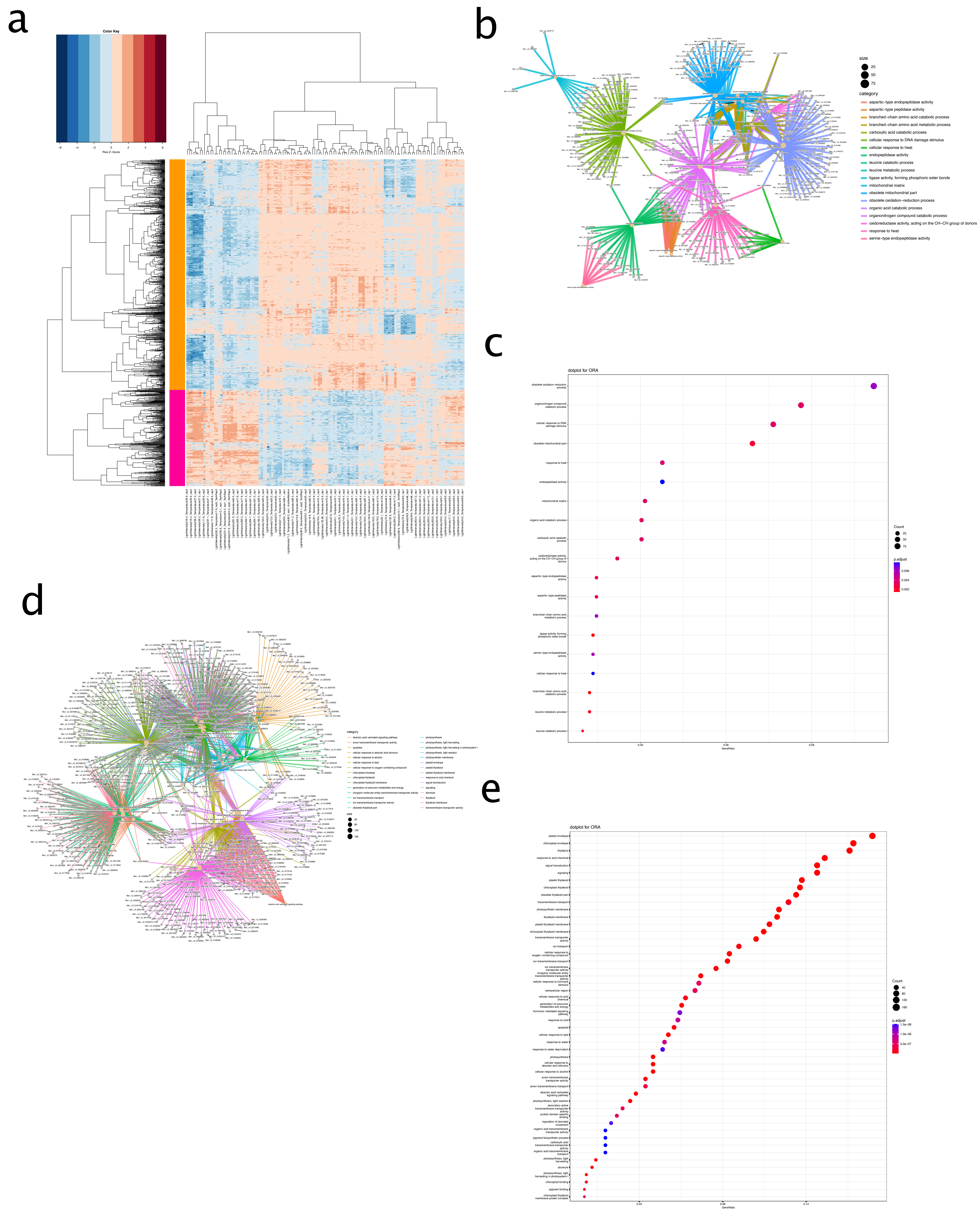

Supplementary Figure S18: DEGs and GO-enrichment of HLI_HT vs. LLI_MT control. (a) Heatmap of DEGs **(b)** cnetplot of orange cluster **(c)** dot plot of orange cluster **(d)** cnetplot of pink cluster **(e)** dot plot of pink cluster. For (c) and (e): The enricher function from clusterProfiler package was used to perform enrichment analysis. A hypergeometric test comparing the set of "significant" genes against the "universe" (or background) genes was performed. Statistics is based on a one-sided test and the Benjamini-Hochberg method was used for adjustment for multiple testing. The adjusted p-values are plotted as color gradient. All p-values, adjusted p-values and q-values are shown in Supplementary Table S14.

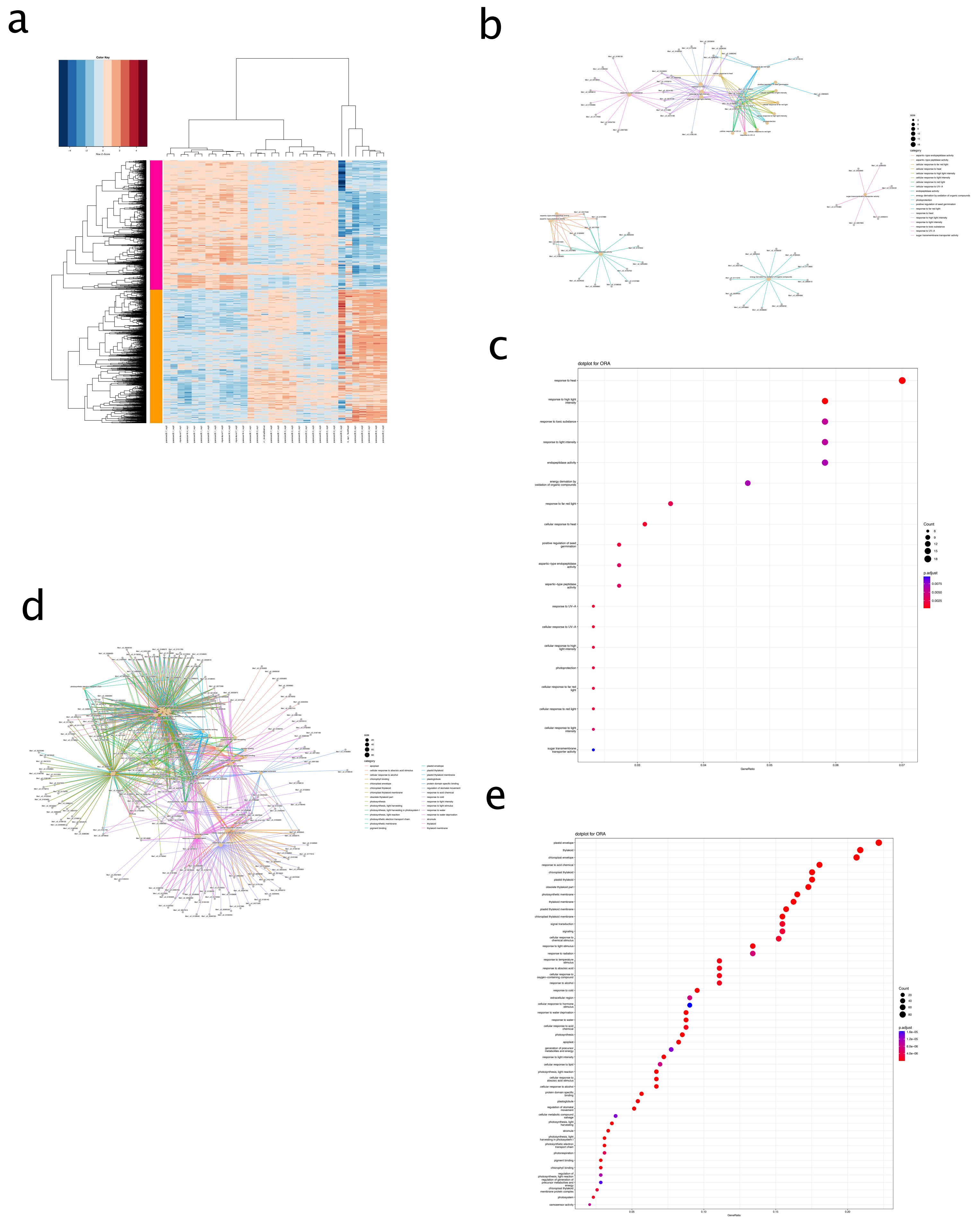

Supplementary Figure S19: DEGs and GO-enrichment of MLI_HT vs. LLI_MT control. (a) Heatmap of DEGs **(b)** cnetplot of orange cluster **(c)** dot plot of orange cluster **(d)** cnetplot of pink cluster **(e)** dot plot of pink cluster. For (c) and (e): The enricher function from clusterProfiler package was used to perform enrichment analysis. A hypergeometric test comparing the set of "significant" genes against the "universe" (or background) genes was performed. Statistics is based on a one-sided test and the Benjamini-Hochberg method was used for adjustment for multiple testing. The adjusted p-values are plotted as color gradient. All p-values, adjusted p-values and q-values are shown in Supplementary Table S14.

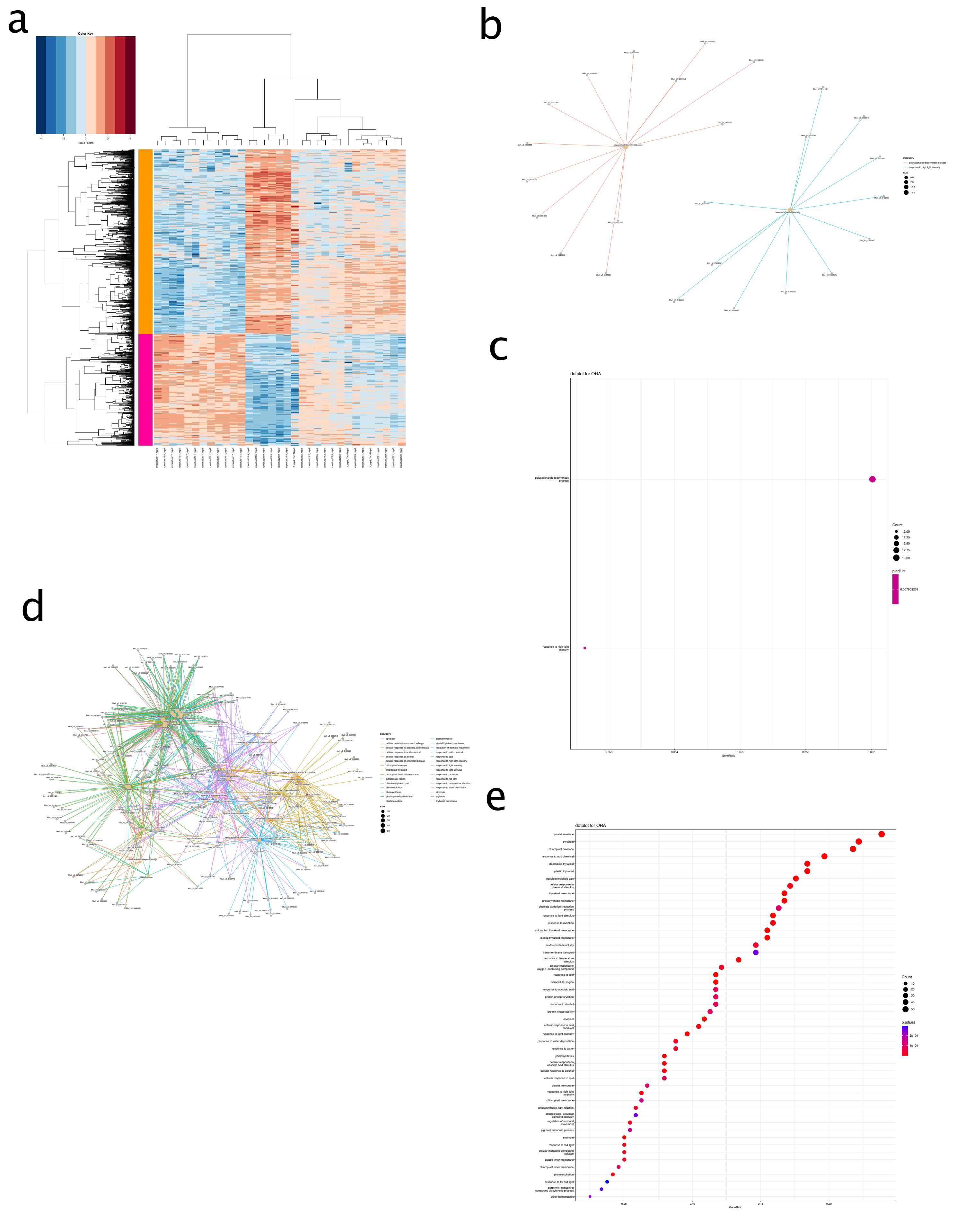

Supplementary Figure S20: DEGs and GO-enrichment of LLI_HT vs. LLI_MT control. (a) Heatmap of DEGs **(b)** cnetplot of orange cluster **(c)** dot plot of orange cluster **(d)** cnetplot of pink cluster **(e)** dot plot of pink cluster. For (c) and (e): The enricher function from clusterProfiler package was used to perform enrichment analysis. A hypergeometric test comparing the set of "significant" genes against the "universe" (or background) genes was performed. Statistics is based on a one-sided test and the Benjamini-Hochberg method was used for adjustment for multiple testing. The adjusted p-values are plotted as color gradient. All p-values, adjusted p-values and q-values are shown in Supplementary Table S14.

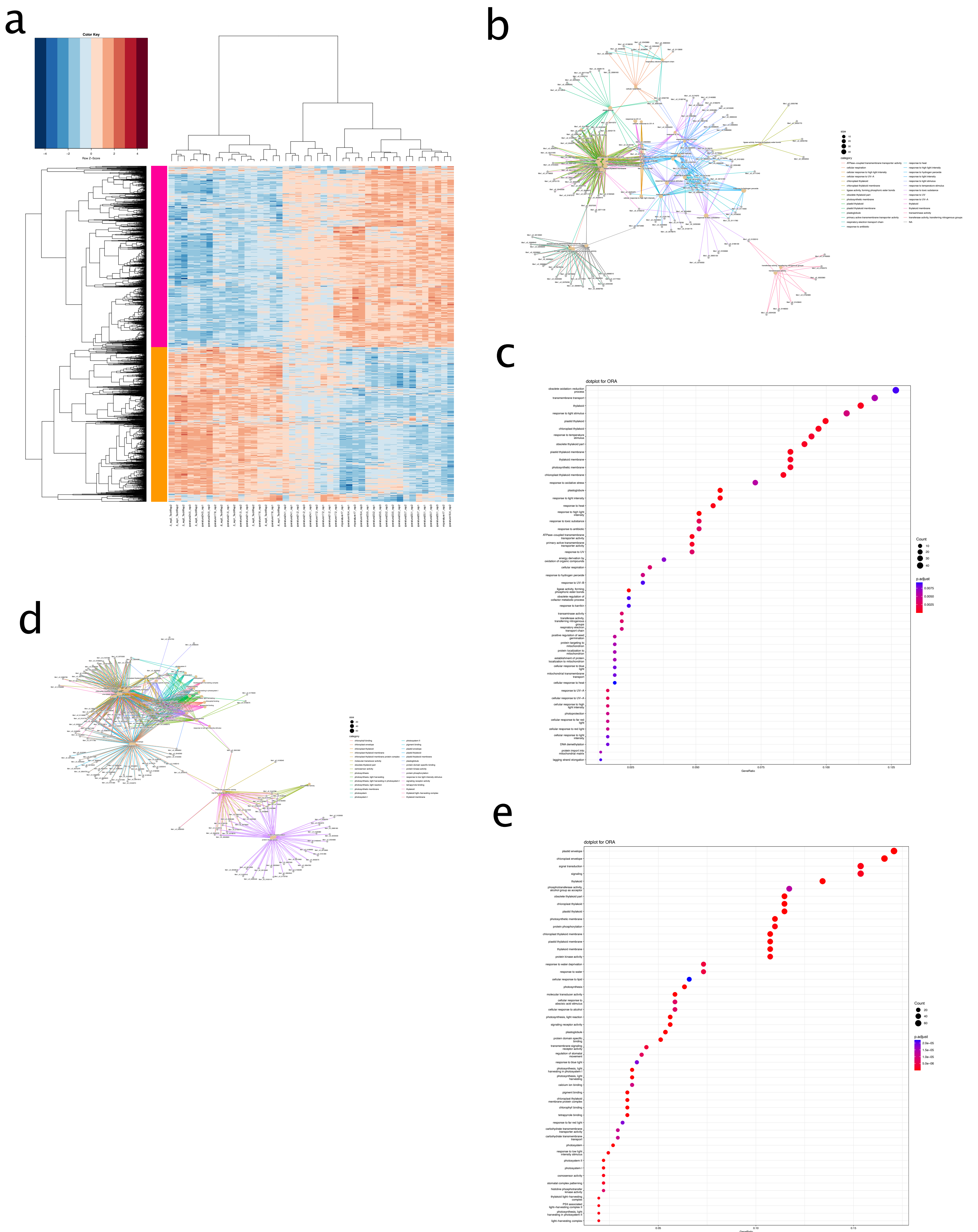

Supplementary Figure S21: DEGs and GO-enrichment of HLI_MT vs. LLI_MT control. (a) Heatmap of DEGs **(b)** cnetplot of orange cluster **(c)** dot plot of orange cluster **(d)** cnetplot of pink cluster **(e)** dot plot of pink cluster. For (c) and (e): The enricher function from clusterProfiler package was used to perform enrichment analysis. A hypergeometric test comparing the set of "significant" genes against the "universe" (or background) genes was performed. Statistics is based on a one-sided test and the Benjamini-Hochberg method was used for adjustment for multiple testing. The adjusted p-values are plotted as color gradient. All p-values, adjusted p-values and q-values are shown in Supplementary Table S14.

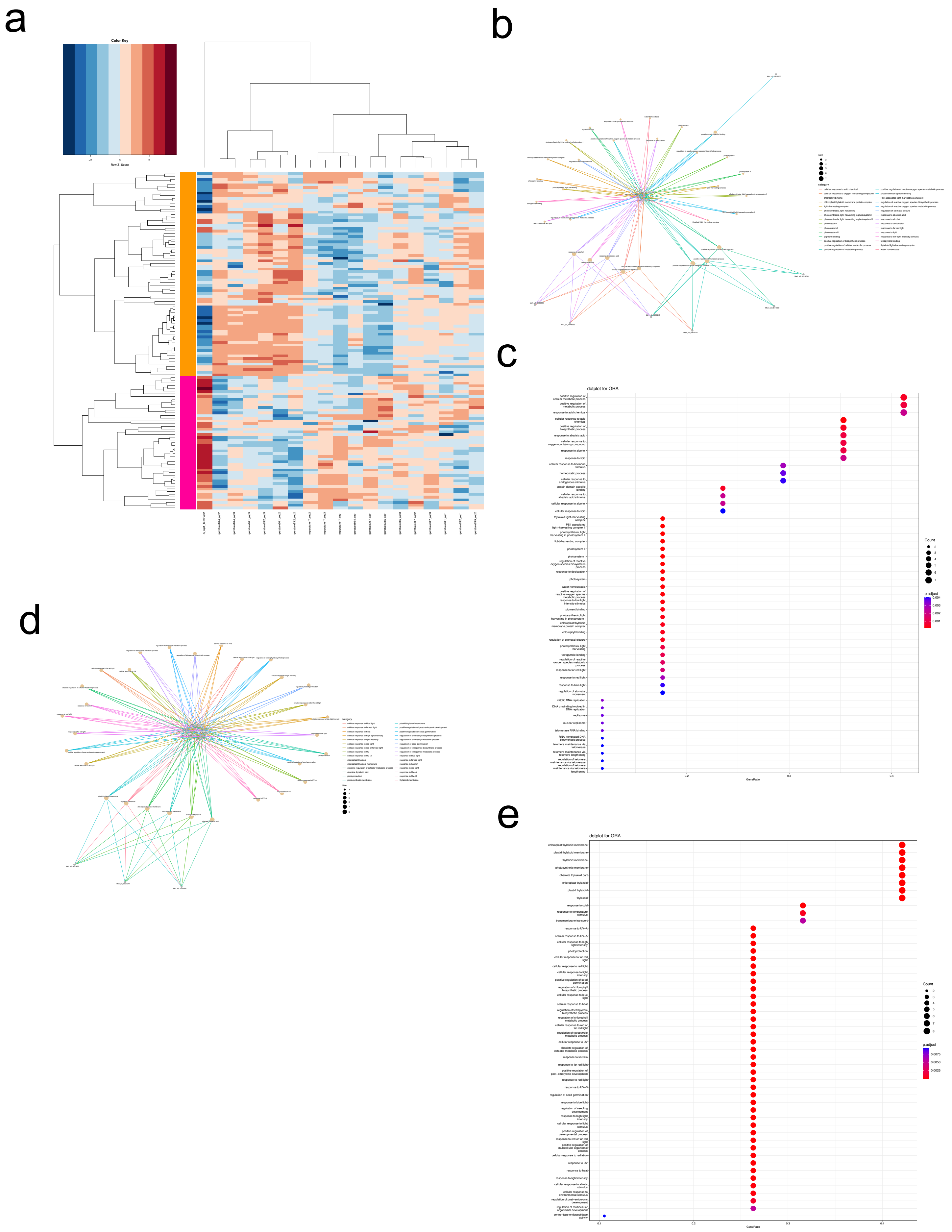

Supplementary Figure S22: DEGs and GO-enrichment of MLI_MT vs. LLI_MT control. (a) Heatmap of DEGs **(b)** cnetplot of orange cluster **(c)** dot plot of orange cluster **(d)** cnetplot of pink cluster **(e)** dot plot of pink cluster. For (c) and (e): The enricher function from clusterProfiler package was used to perform enrichment analysis. A hypergeometric test comparing the set of "significant" genes against the "universe" (or background) genes was performed. Statistics is based on a one-sided test and the Benjamini-Hochberg method was used for adjustment for multiple testing. The adjusted p-values are plotted as color gradient. All p-values, adjusted p-values and q-values are shown in Supplementary Table S14.

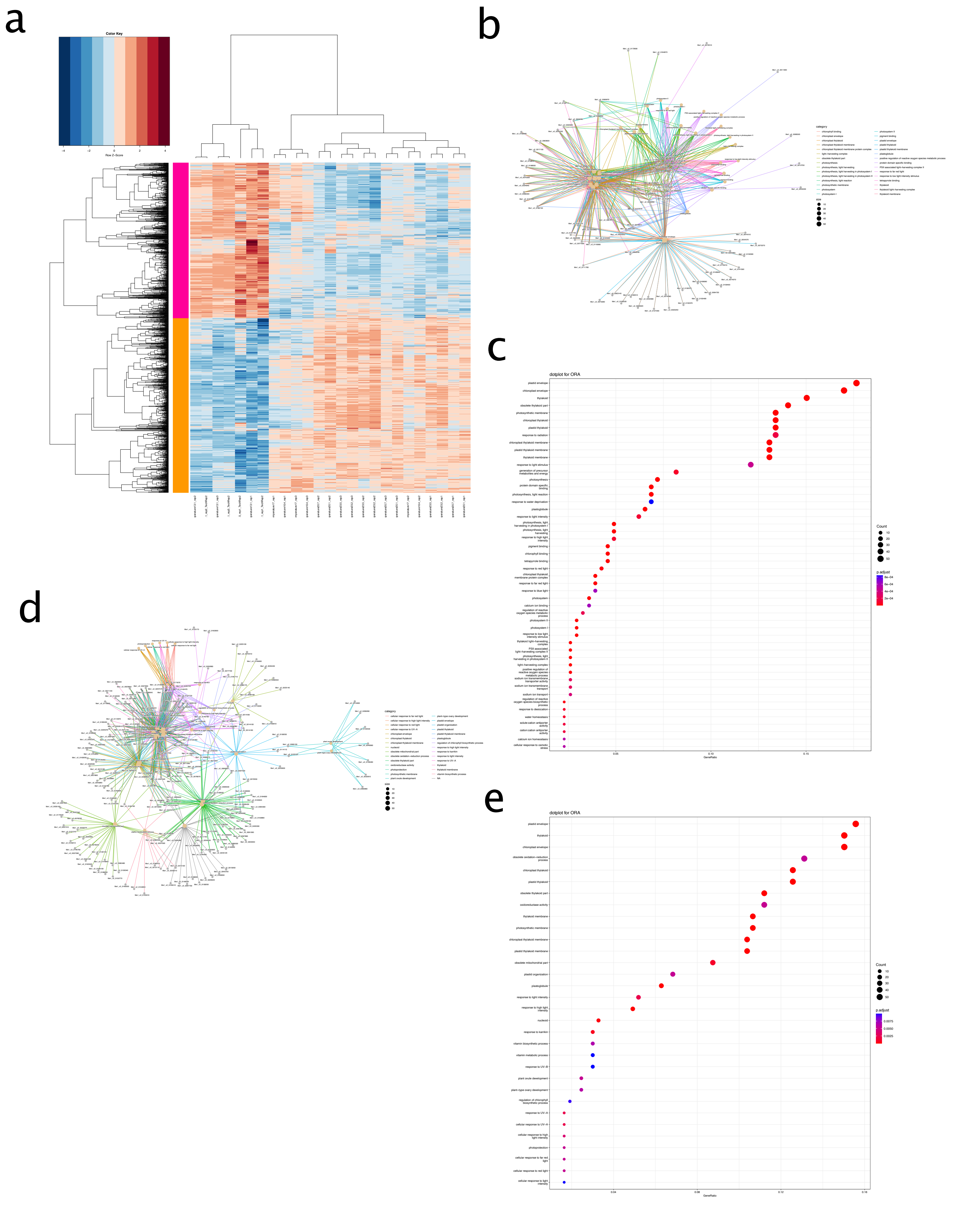

Supplementary Figure S23: DEGs and GO-enrichment of HLI_LT vs. LLI_MT control. (a) Heatmap of DEGs **(b)** cnetplot of orange cluster **(c)** dot plot of orange cluster **(d)** cnetplot of pink cluster **(e)** dot plot of pink cluster. For (c) and (e): The enricher function from clusterProfiler package was used to perform enrichment analysis. A hypergeometric test comparing the set of "significant" genes against the "universe" (or background) genes was performed. Statistics is based on a one-sided test and the Benjamini-Hochberg method was used for adjustment for multiple testing. The adjusted p-values are plotted as color gradient. All p-values, adjusted p-values and q-values are shown in Supplementary Table S14.

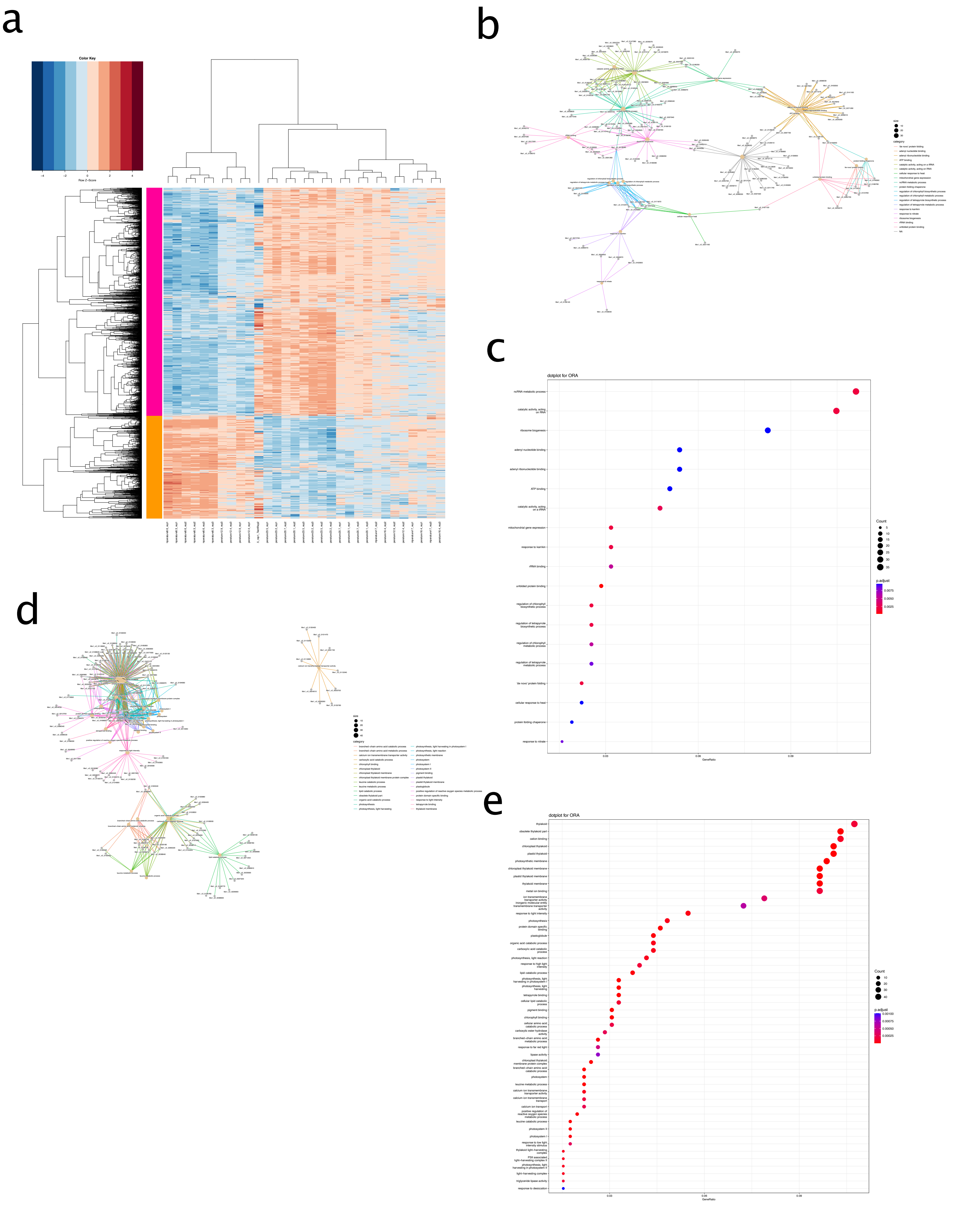

Supplementary Figure S24: DEGs and GO-enrichment of MLI_LT vs. LLI_MT control. (a) Heatmap of DEGs **(b)** cnetplot of orange cluster **(c)** dot plot of orange cluster **(d)** cnetplot of pink cluster **(e)** dot plot of pink cluster. For (c) and (e): The enricher function from clusterProfiler package was used to perform enrichment analysis. A hypergeometric test comparing the set of "significant" genes against the "universe" (or background) genes was performed. Statistics is based on a one-sided test and the Benjamini-Hochberg method was used for adjustment for multiple testing. The adjusted p-values are plotted as color gradient. All p-values, adjusted p-values and q-values are shown in Supplementary Table S14.

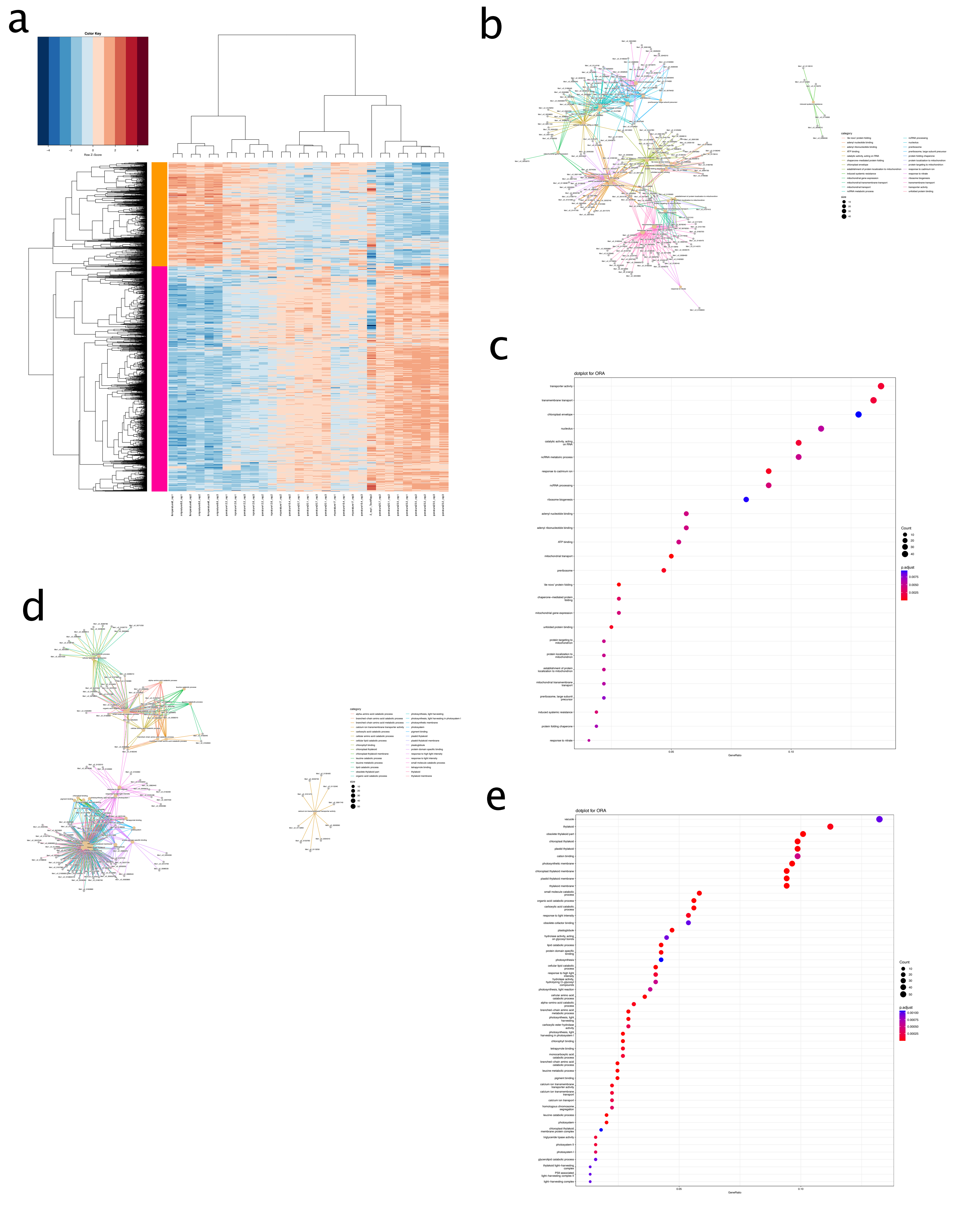

Supplementary Figure S25: DEGs and GO-enrichment of LLI_LT vs. LLI_MT control. (a) Heatmap of DEGs **(b)** cnetplot of orange cluster **(c)** dot plot of orange cluster **(d)** cnetplot of pink cluster **(e)** dot plot of pink cluster. For (c) and (e): The enricher function from clusterProfiler package was used to perform enrichment analysis. A hypergeometric test comparing the set of "significant" genes against the "universe" (or background) genes was performed. Statistics is based on a one-sided test and the Benjamini-Hochberg method was used for adjustment for multiple testing. The adjusted p-values are plotted as color gradient. All p-values, adjusted p-values and q-values are shown in Supplementary Table S14.

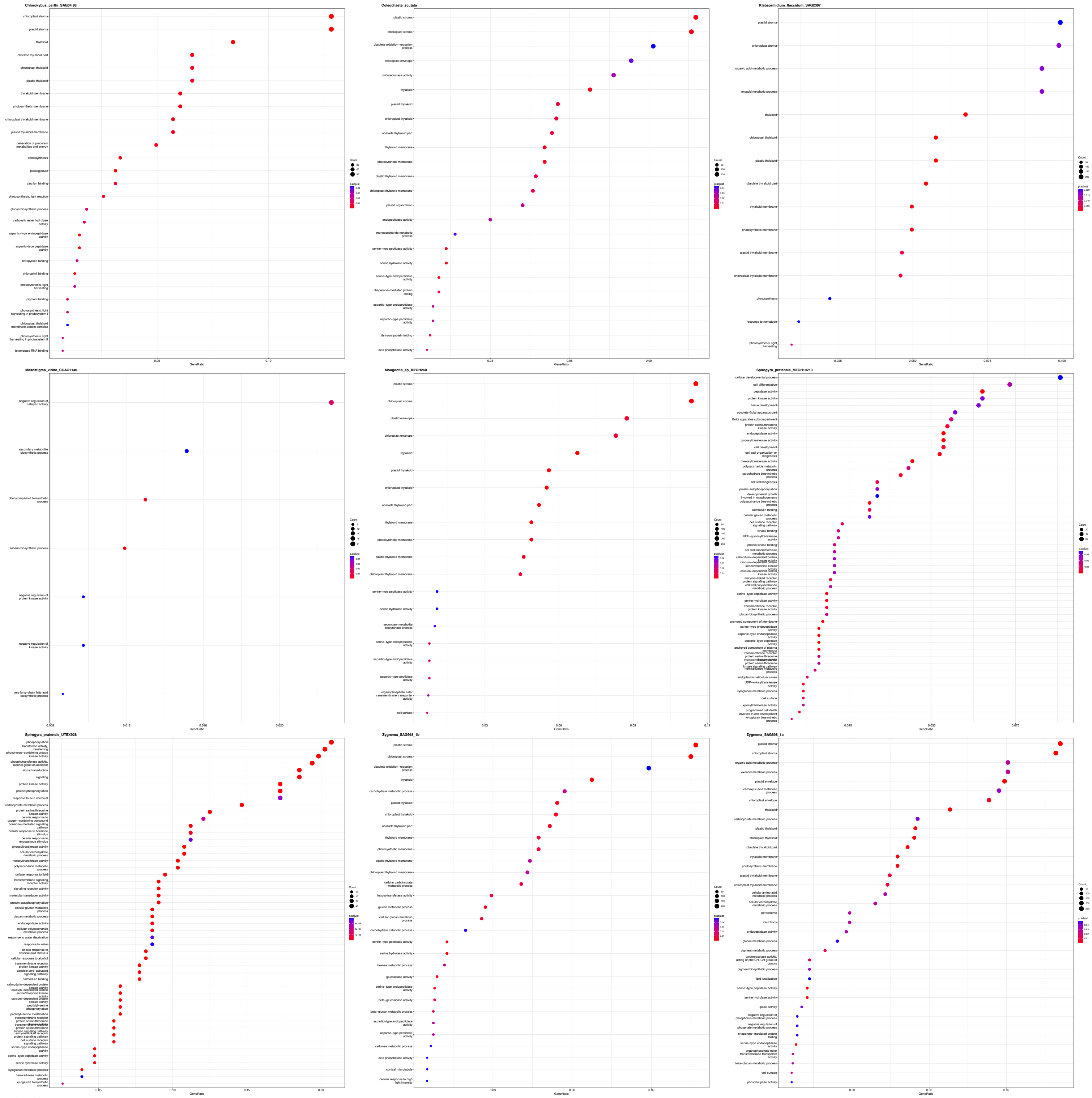

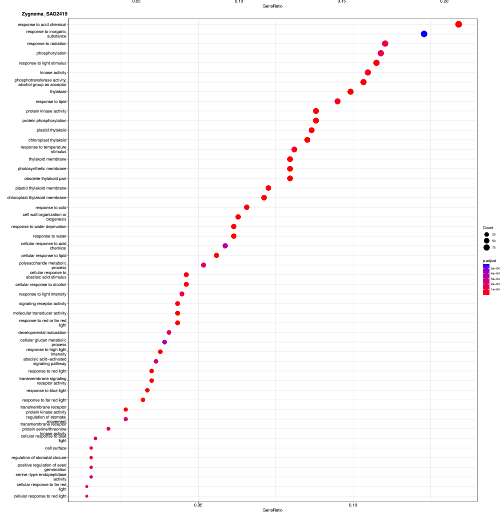

Supplementary Figure S26A: Pairwise GO enrichment analysis of HOGs that are regulated in each streptophyte alga and *Mesotaenium*. We only visualized GO terms that has adj. p-value < 0.05.The enricher function from clusterProfiler package was used to perform enrichment analysis. A hypergeometric test comparing the set of "significant" genes against the "universe" (or background) genes was performed. Statistics is based on a one-sided test and the Benjamini-Hochberg method was used for adjustment for multiple testing. The adjusted p-values are plotted as color gradient. All p-values, adjusted p-values and q-values are shown in Supplementary Table S15.

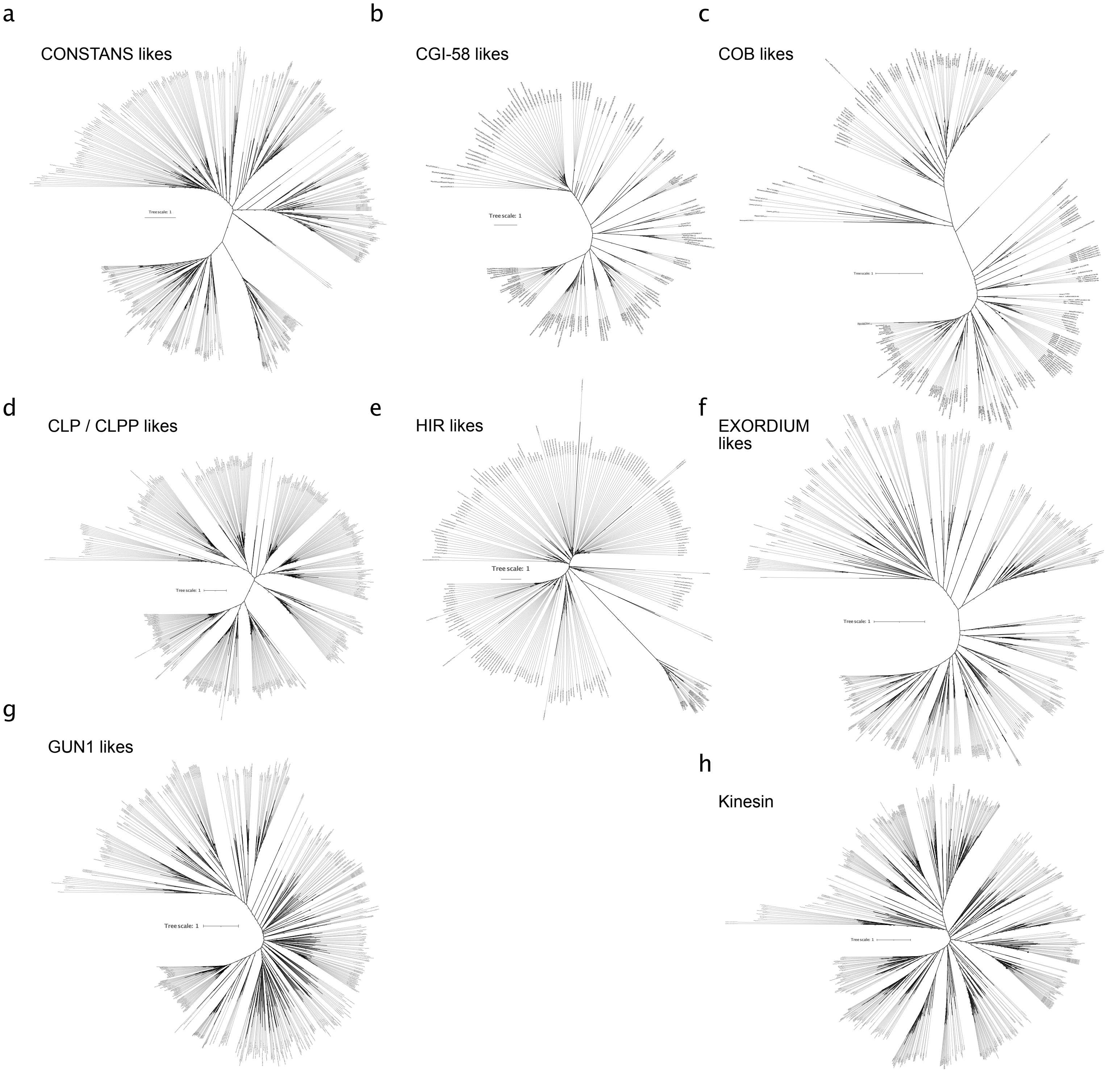

Supplementary Figure S26B: Fully-labeled phylogenies of hub genes. (a) CONSTANS like **(b)** CGI-58 likes **(c)** COB likes **(d)** CLP / CLPP likes **(e)** HIR likes **(f)** EXORDIUM likes **(g)** GUN1 likes **(h)** Kinesin. All phylogenies were computed with IQ-TREE multicore version 1.5.5, their respective best model according to Bayesian Information Criterion and 1000 ultrafast bootstrap replicates.

a

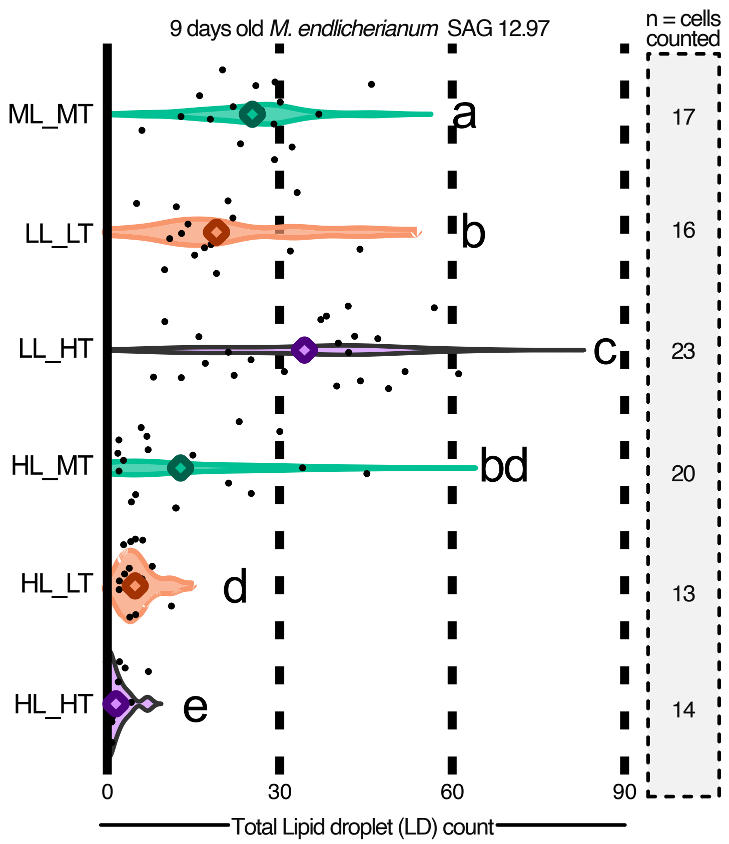

b

sample		16:0	16:1 (9Z)	18:0	18:1 (9Z)	18:1 (11Z)	18:2 (9Z,12Z)	18:3 (6Z,9Z,12Z)	18:3 (9Z,12Z,15Z)	Total lipid content
12 weeks	**TAG**	44.23	3.03	11.97	246.93	7.10	41.28	0.43	3.78	**358.75**
	FFA	3.98	0.09	2.73	2.02	0.18	0.60	0.15	0.18	9.92
	DAG	1.84	0.10	1.52	1.09	0.08	0.56	0.15	0.19	5.54
21 weeks	**TAG**	6.32	0.91	3.89	2.94	0.18	1.85	1.44	0.62	**18.14**
	FFA	3.20	0.09	3.85	0.77	0.07	0.16	0.28	0.12	8.53
	DAG	2.90	0.16	2.93	1.07	0.16	0.26	0.27	0.21	7.97
25 weeks	**TAG**	6.52	0.23	4.19	3.16	0.21	1.88	0.68	0.58	**17.46**
	FFA	4.87	0.11	6.22	0.61	0.04	0.13	0.18	0.10	12.26
	DAG	2.72	0.20	3.48	0.77	0.05	0.17	0.34	0.21	7.94

[µg/preparation]

Supplementary Figure S27: Lipid droplet count setup 2. (A) Violin plots of an additional LD quantification after 9 days of exposure to different environmental conditions including statistical analysis using Mann-Whitney U statistics (significance grouping based on p value < 0.05). (B) Quantification and profiling of triacylglycerol (TAG), free fatty acids (FFA), and diacylglycerol (DAG) determined based on gas chromatography (GC) that follwed a preparative TLC (see main Figure 6f).

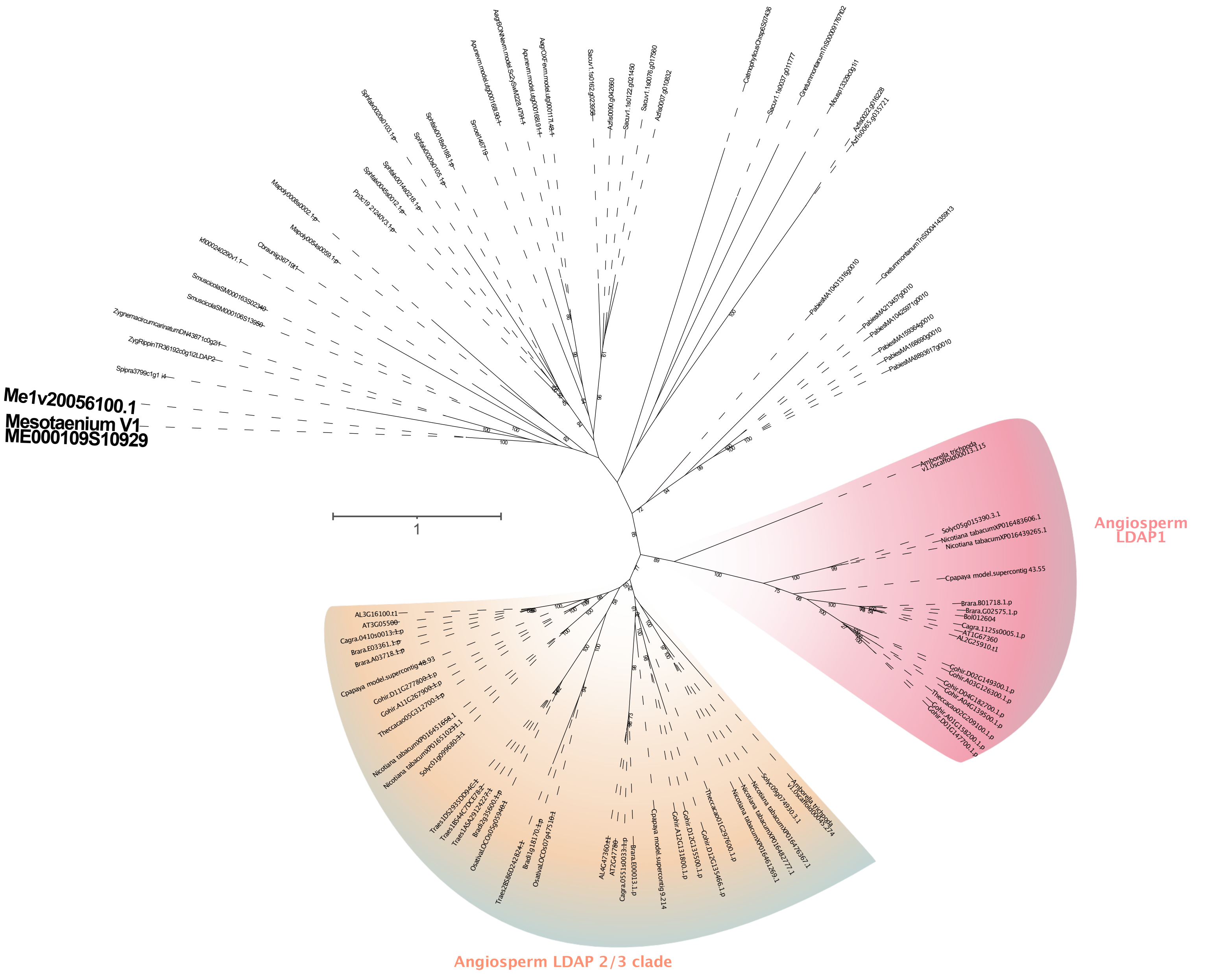

Supplementary Figure S28: LDAP phylogeny. Maximum likelihood phylogeny computed with IQ-TREE multicore version 1.5.5 based on a MAFFT L-INS-I alignment. Using ModelFinder, we determined JTT+I+G4 as best model for protein evolution based on Bayesian Information Criterion. The phylogeny was based on the alignment in de Vries and Ischebeck (2020) after adding the annotation V2 homolog of MeLDAP (Me1v20056100.1).
