# Supplementary material for: Environmental gradients reveal stress hubs pre-dating plant terrestrialization
Source: Nat Plants. 2023 Aug 28;9(9):1419–38. doi: 10.1038/s41477-023-01491-0 (PMC10505561; doi:10.1038/s41477-023-01491-0)
Supplement: Supplementary file 2 — Reporting Summary [file 41477_2023_1491_MOESM2_ESM.pdf]

nature portfolio

Corresponding author(s): Jan de Vries

Last updated by author(s): Jul 4, 2023

Reporting Summary

Nature Portfolio wishes to improve the reproducibility of the work that we publish. This form provides structure for consistency and transparency in reporting. For further information on Nature Portfolio policies, see our Editorial Policies and the Editorial Policy Checklist.

Statistics

For all statistical analyses, confirm that the following items are present in the figure legend, table legend, main text, or Methods section.

n/a	Confirmed	
☐	☒	The exact sample size (*n*) for each experimental group/condition, given as a discrete number and unit of measurement
☐	☒	A statement on whether measurements were taken from distinct samples or whether the same sample was measured repeatedly
☐	☒	The statistical test(s) used AND whether they are one- or two-sided<br>*Only common tests should be described solely by name; describe more complex techniques in the Methods section.*
☐	☒	A description of all covariates tested
☐	☒	A description of any assumptions or corrections, such as tests of normality and adjustment for multiple comparisons
☐	☒	A full description of the statistical parameters including central tendency (e.g. means) or other basic estimates (e.g. regression coefficient) AND variation (e.g. standard deviation) or associated estimates of uncertainty (e.g. confidence intervals)
☐	☒	For null hypothesis testing, the test statistic (e.g. *F*, *t*, *r*) with confidence intervals, effect sizes, degrees of freedom and *P* value noted<br>*Give P values as exact values whenever suitable.*
☒	☐	For Bayesian analysis, information on the choice of priors and Markov chain Monte Carlo settings
☒	☐	For hierarchical and complex designs, identification of the appropriate level for tests and full reporting of outcomes
☐	☒	Estimates of effect sizes (e.g. Cohen's *d*, Pearson's *r*), indicating how they were calculated

Our web collection on statistics for biologists contains articles on many of the points above.

Software and code

Policy information about availability of computer code

Data collection	Absorption were measured and averaged using software Gen5 v(2.0). Photophysiological measurements via the IMAGING PAM have been done via ImagingWinGigE (V2.32) software. Sequencing data was collected on an Illumina NovaSeq6000 platform operated by Novogene UK. wget (GNU Wget 1.14) was used to download genomic sequences from Phytozome, Novogene, DDBJ, Figshare, Fernbase, and TAIR databases. LC-MS method programming and data aquisition for raw proteome data was performed with the XCalibur 4.0 software (Thermo Fisher Scientific).
Data analysis	We installed all packages (and their dependencies) using conda if possible. Otherwise, we followed the installation guide offered by the authors of the tool. For R packages, we installed them from, in this order of priorities, from Cran repository, BioCmanager, or the tool GitHub instructions. All codes used to perform computational analyses are available in our GitHub repository:<br>https://github.com/deVries-lab/Response_to_a_gradient_of_environmental_cues_in_mesotaenium_endlicherianum<br><br>Absorption were meassured and averaged using software Gen5 v(2.0). Photophysiological measurements via the IMAGING PAM have been done via ImagingWinGigE (V2.32) software.<br><br>For statistical analyses of absoption and Fv/Fm values and tempearture/light cluster analysis, we used these set of tools:<br>Kruskal-Wallis test with post hoc test Fisher's least significant difference using R (4.1.3). P-values were Bonferroni corrected and grouped into significant groups using R packages 'agricolae' (v1.3-5) and 'dplyr' version (v1.0.9), pheatmap (v1.0.12), from the factoextra package (v1.0.7) we used eclust function with clustering function 'kmeans', with number of clusters set to six and for hierarchical clustering 'euclidean' was used as distance measure.<br><br>The quality of raw RNA-Seq reads were checked with FastQC (v0.11.9) and summarized with MultiQC (v1.11). Reads were trimmed and

filtered via Trimmomatic (v0.36) with these parameters:
("ILLUMINACLIP: novogene_adapter_sequences_Trimmomatic.fa:2:30:10:2:True
418 LEADING:26 TRAILING:26 SLIDINGWINDOW:4:20 MINLEN:36")
The quality of trimmed and filtered reads were again checked by FastQC (v0.11.9) and MultiQC (v1.11).
For genome re-annotation we used a diverse set of bioinformatic tools including:
HISAT2 (v2.2.1), StringTie(v2.1.5), Busco (v5.3.2), REAT (v0.6.1, https://reat.readthedocs.io/en/latest/), scallop (v0.10.5), Portcullis (v1.2.4), Mikado (v2.3.4), SPALN (v2.4.7), Augustus (v3.4.0), SNAP (v2006-07-28), Glimmer (v0.3.2), CodingQuarry (v2.0), EvidenceModeler (v1.1.1), Minos (v1.8.0; https://github.com/EI-CoreBioinformatics/minos), Diamond (v0.9.34), Kallisto (v0.46.2), CPC2 (v0.1), maker (v3.01.04), interproscan (v0.9.2), agat (v0.9.2), eggNOGmapper (v2.1.8)

For RNA-Seq quantification and differential gene expression analyses we used this set of software:
Snakemake((7.7.0), Kallisto (v0.45.0), R (v4.2.0), tximport (v1.24.0) with "lengthScaledTPM" option, tidyverse (v1.3.1), edgeR (v3.38.1) "calcNormFactors method=TMM", limma (v3.52.2), ggplot2 (v3.3.6), pheatmap (v1.0.12) with "clustering_distance_rows = "euclidean" and clustering_distance_cols = "euclidean"", clusterProfiler (v4.4.4) with p-value and q-value cutoff of 0.01 and all genes that passed low-expression filtering as background universe

We performed Weighted gene co-expression analyses via:
WGCNA (v1.71), tidyverse (v1.3.1), clusterProfiler (v4.4.4) with p-value and q-value cutoff of 0.05 and all genes that passed low-expression filtering as background universe, eggNOGmapper (v2.1.8), BLAST (v2.11.0+)

We performed Phylogenetic analyses using:
BLAST (v2.11.0+), MAFFT (v7.490), IQ-TREE (v1.5.5), ModelFinder (integrated in IQ-TREE multicore version 1.5.5 for Linux 64-bit built Jun 2 2017) according to Bayesian Information Criterion and 1000 ultrafast bootstrap replicates; 1000 ultrafast bootstrap replicates were carried out 100 Felsenstein bootstraps. We colored phylogeny trees via ggtree (v3.9.0)

Protein domains were predicted using Interproscan (v5.59-91.0)

For transcriptome analyses of published data from other algae, we used Trinity (v2.15.1), Transdecoder (v5.7.0), and ComBat-seq.
For orthogroup analyses we used BioNERO and Orthofinder

Proteome data were analyzed using Max Quant software version 1.6.2.10 (Cox and Mann, 2008) and further processed using Perseus (1.6.2.2) software.

Gas-chromatograms were analysed using the ChemStation software.

For manuscripts utilizing custom algorithms or software that are central to the research but not yet described in published literature, software must be made available to editors and reviewers. We strongly encourage code deposition in a community repository (e.g. GitHub). See the Nature Portfolio guidelines for submitting code & software for further information.

Data

Policy information about availability of data

All manuscripts must include a data availability statement. This statement should provide the following information, where applicable:

- Accession codes, unique identifiers, or web links for publicly available datasets
- A description of any restrictions on data availability
- For clinical datasets or third party data, please ensure that the statement adheres to our policy

All RNAseq reads have been uploaded to NCBI SRA and can be accessed under Bioproject PRJNA832564 and SRA accessions SRR18936040 to SRR18936170.
Furthermore, data can be interactively explored at https://mesotaenium.uni-goettingen.de
Proteomic data have been uploaded to EMBL-EBI PRIDE (accession PXD037847).
On Zenodo, we have deposited: (i) raw light and confocal micrographs generated, e.g. for lipid droplet assessment in Mesotaenium and pollen tubes https://doi.org/10.5281/zenodo.7921367 and (ii) Raw and visualized phylogenetic data https://doi.org/10.5281/zenodo.7950653
The additional previously published RNAseq datasets that were used for comparisons are: (i) A. thaliana: SRR2302908 to SRR2302919, ERR754084, ERR754066, ERR754077, ERR754069, ERR754087, ERR754064, ERR754059, SRR7659142, SRR7659143, SRR7659144, SRR7659145 to SRR7659150, SRR5197904, to SRR5197909; (ii) M. polymorpha: SRR12076853, SRR12076855, SRR12076857, SRR12076859, SRR12076861, SRR12076863, SRR12076865, SRR12076867, SRR12076869, SRR12076871, SRR12076873, SRR12076875, SRR12076877, SRR12076879, SRR12076917 to SRR12076925, SRR15186078 to SRR15186125, DRR093991 to DRR093996; (iii) P. patens: SRR1824306 to SRR1824320, SRR10235460 to SRR10235483, SRR787291, SRR787292, SRR787293, SRR787294, SRR787295; (iv) Z. circumcarinatum SAG698-1b: SRR24939299, SRR24940177, SRR24909175, SRR24757807, SRR24757829, SRR24757830, SRR24757831, SRR24205691 to SRR24205702, SRR24286545 to SRR24286562, SRR24576622, SRR24576623, SRR24385702, SRR24450996, SRR24450997, SRR24451196, SRR24480449, SRR24707416, SRR24707417, SRR24952091, SRR21891679 to SRR21891705; (v) C. cerffii (at the time, C. atmophyticus, see ref. 97): SRR5949009, SRR5949013 to SRR5949016, SRR5949027 to SRR5949030; (vi) C. scutata: SRR5948993, SRR5948995 to SRR5948998, SRR5949001, SRR5949004, SRR5949005, SRR5949007; (vii) K. flaccidum: SRR5949010, SRR5949011, SRR5949012, SRR5990072 to SRR5990080; (viii) M. viride: SRR5949021 to SRR5949026; (ix) Mougeotia sp. MZCH240: SRR9083681, SRR9083682, SRR9083688, SRR9083692 to SRR9083701; (x) S. pratensis MZCH10213: SRR9083685, SRR9083686, SRR9083687, SRR9083689, SRR9083690, SRR9083696; (xi) S. pratensis UTEX928: SRR4018077 to SRR4018100; (xii) Z. circumcarinatum SAG698-1a: SRR5948999, SRR5949000, SRR5949002, SRR5949003, SRR5949006, SRR5949008, SRR5949017, SRR5949018; and (xiii) Z. circumcarinatum SAG2419: SRR6047298, SRR6047299, SRR6047302 to SRR6047305.

Human research participants

Policy information about studies involving human research participants and Sex and Gender in Research.

Reporting on sex and gender	n/a
Population characteristics	n/a
Recruitment	n/a
Ethics oversight	n/a

Note that full information on the approval of the study protocol must also be provided in the manuscript.

Field-specific reporting

Please select the one below that is the best fit for your research. If you are not sure, read the appropriate sections before making your selection.

☒ Life sciences ☐ Behavioural & social sciences ☐ Ecological, evolutionary & environmental sciences

For a reference copy of the document with all sections, see nature.com/documents/nr-reporting-summary-flat.pdf

Life sciences study design

All studies must disclose on these points even when the disclosure is negative.

Sample size	Each analysis involved millions of pooled cells (cultures were set to a density of 20,300,000 cells/ml), all can be assumed to behave similarly (as they were vegetative cells from the same starting culture). For each experiment, 504 Mesotaenium endlicherianum samples were analyzed, pooled to 42 samples for RNAseq and averaged to 42 physiological/growth data points (measurements for all 504 samples are provided in the supplement). Sequencing was then performed to a depth that was chosen based on approaching saturation level (based on obtaining differential expression patterns among the given number of genes).
Data exclusions	No data were excluded.
Replication	For the physiological and morphological analysis as well as RNAseq, three independent experiments were conducted. Preexperiments for 8 days were carried out two times, 31 March 2021 to 8 April 2021 and 26 May 2021 to 4 June 2021. For the final 3 day-lasting experiments for RNAseq combined with physiological assessment the gradient table was run three times independently in November 2021 (8 Nov. 21 to 11. Nov. 21; 15. Nov. 21 to 18. Nov. 21; 23. Nov. 21 to 26. Nov. 21). In this final setup, all attempts at replication were successful. For proteomic, analysis of two samples with a successfully enriched lipid droplet fraction and the corresponding total extract fraction, both isolated from the alga Mesotaenium endlicherianum, were used. For lipid droplet counts, hundreds of cells were assessed, indicated in the figure.
Randomization	All experiments are based on a random selection of millions of cells from a liquid culture. The start culture was one homogenous culture that was equally and distributed. A random selection of millions of cells thus ended up in one plate that was exposed to a certain condition. Prior to start, all plates were thus equal.
Blinding	Blinding was not relavant for this study. It is irrelevant for the bioinformatics because we worked with all versus all comparisons, unsupervised methods and all pipelines are fully transparent on GitHub. All cell-based evaluation is quantifyable and unambigous (counts of lipid droplets). Further, the information is fully provided and re-evaluatable.

Reporting for specific materials, systems and methods

We require information from authors about some types of materials, experimental systems and methods used in many studies. Here, indicate whether each material, system or method listed is relevant to your study. If you are not sure if a list item applies to your research, read the appropriate section before selecting a response.

Materials & experimental systems

n/a	Involved in the study	
☒	☐	Antibodies
☐	☒	Eukaryotic cell lines
☒	☐	Palaeontology and archaeology
☒	☐	Animals and other organisms
☒	☐	Clinical data
☒	☐	Dual use research of concern

Methods

n/a	Involved in the study	
☒	☐	ChIP-seq
☒	☐	Flow cytometry
☒	☐	MRI-based neuroimaging

Eukaryotic cell lines

Policy information about cell lines and Sex and Gender in Research

Cell line source(s)	Mesotaenium endlicherianum (Zygnematophyceae, Streptophyta): The alga has the collection number SAG 12.97. The biomaterial provider is the Experimental Phycology and Culture Collection of Algae in Göttingen, Germany. The alga was originally isolated in Portugal, Quiaios, Lagoa das Bracas, plankton, Lat./Long.(Precision): 40.243191 / -8.80488.
Authentication	Authentication was carried out directly by the biomaterial provider, the Experimental Phycology and Culture Collection of Algae in Göttingen, Germany, via microscopy and genetic markers.
Mycoplasma contamination	n/a
Commonly misidentified lines (See ICLAC register)	n/a
