# Supplementary material for: Environmental gradients reveal stress hubs pre-dating plant terrestrialization
Source: Nat Plants. 2023 Aug 28;9(9):1419–38. doi: 10.1038/s41477-023-01491-0 (PMC10505561; doi:10.1038/s41477-023-01491-0)
Supplement: Source Data Fig. 6 — Whole scan of the TLC, shown in Fig. 6g. [file 41477_2023_1491_MOESM4_ESM.pdf]

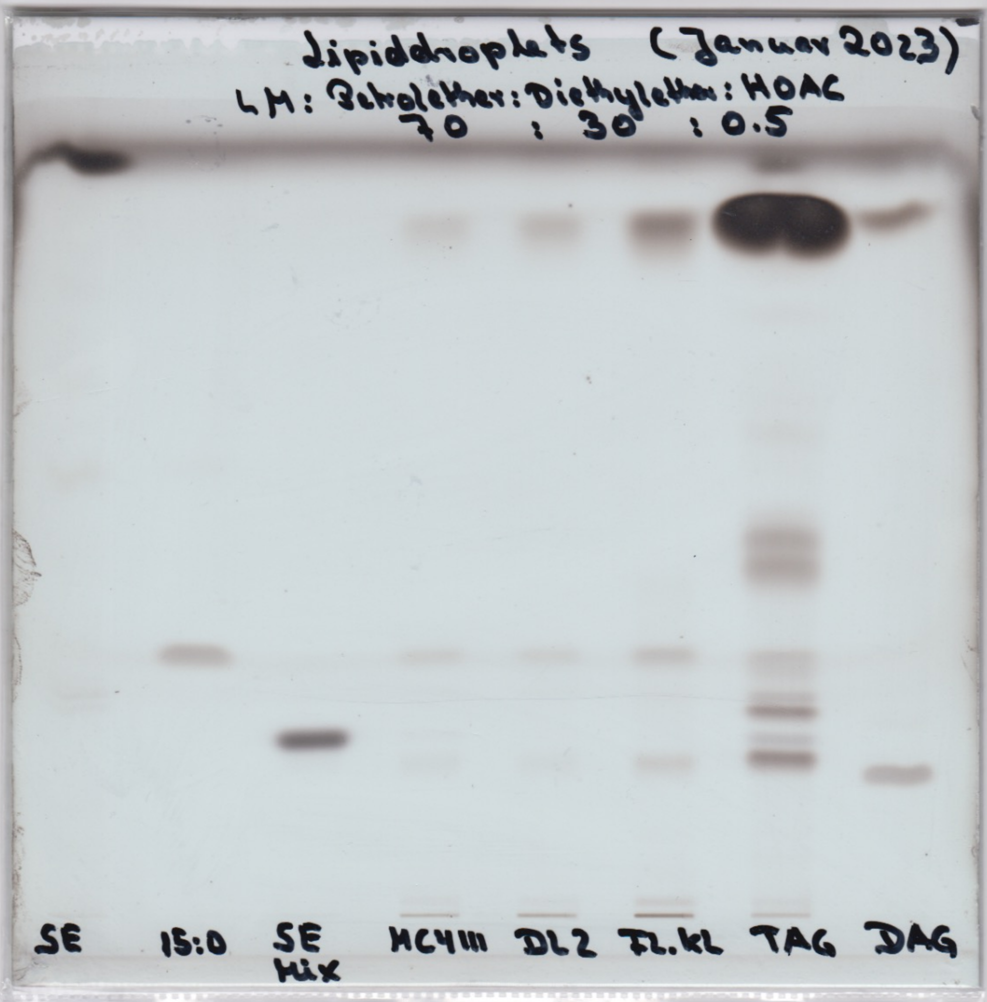
Lipiddroplets (Januar 2023)
LM: Petrolether : Diethylether : HOAC
70 : 30 : 0.5
SE
15:0
SE Mix
MC4III
DL2
Fl.kl
TAG
DAG
